# A survey of *Hebeloma* (Hymenogastraceae) in Greenland

**DOI:** 10.3897/mycokeys.79.63363

**Published:** 2021-04-19

**Authors:** Ursula Eberhardt, Henry J. Beker, Torbjørn Borgen, Henning Knudsen, Nicole Schütz, Steen A. Elborne

**Affiliations:** 1 Staatliches Museum für Naturkunde Stuttgart, Rosenstein 1, D-70191 Stuttgart, Germany; 2 Rue Pére de Deken 19, B-1040 Bruxelles, Belgium; 3 Royal Holloway College, University of London, Egham, UK; 4 Plantentuin Meise, Nieuwelaan 38, B-1860 Meise, Belgium; 5 Sensommervej 142, 8600 Silkeborg, Denmark; 6 Hauchsvej 15, 1825 Frederiksberg, Denmark; 7 Frederik VII´s Vej 29, 3450 Allerød, Denmark

**Keywords:** Arctic distribution, High Arctic, Low Arctic, mycorrhizal hosts, new species, pruned median joining networks

## Abstract

This is the first study exclusively dedicated to the study of *Hebeloma* in Greenland. It is based on almost 400 collections, the great majority of which were collected by three of the co-authors over a period of 40 years and were lodged in the fungarium of the Natural History Museum in Copenhagen. The material was identified using molecular and morphological methods. In total, 28 species were recognized, 27 belonging to three sections, *H.* sects *Hebeloma*, *Denudata* and *Velutipes*. One species sampled was new to science and is here described as *H. arcticum*. For all species, a description, a distribution map within Greenland and macro and microphotographs are presented. A key is provided for the 28 species. The distribution of species within Greenland is discussed. The findings are placed in the context of studies of arctic and alpine *Hebeloma* from other parts of the world where comparable data exist. Notably, *H. grandisporum*, *H. louiseae* and *H. islandicum*, previously only known from Romania, Svalbard, Iceland or Norway, respectively, have been found in Greenland. The latter is also the only species encountered that does not belong to any of the above sections. *Hebeloma excedens* and *H. colvinii* – for the latter we here publish the first modern description – are to date only known from continental North America and now Greenland.

## Introduction

*Hebeloma* are notoriously difficult to identify to species. The genus is very common in arctic habitats and plays an important role as a mycorrhizal symbiont in arctic scrubland for plants like *Salix*, *Betula* and *Dryas* and thus for the turnover of nutrients in these harsh environments. In spite of their frequency and abundance in arctic areas, the alpha taxonomy of the genus was confused until the work of Beker and colleagues (Eberhardt et al. 2015a, b, 2016; Beker et al. 2016; Grilli et al. 2016) who described a number of new species from Europe, including the arctic and alpine regions of Europe, and reappraised the delimitation of known taxa.

Here we provide a review of *Hebeloma* spp. collected in Greenland and verified by molecular and morphological analysis. These encompass 378 of the 405 collection from Greenland that were digitized in the fungarium C of the Natural History Museum in Copenhagen, and represent 28 species. The great majority of this material was collected by three of the authors (T.B., S.A.E. and H.K.K.) over a period of 40 years in preparation for a forthcoming “Funga Arctica & Alpina” of basidiomycetes. The material presented here is a part of the 15.000 collections for the funga.

Each of the 28 species is presented with a morphological description, a photo of the macroscopic characters, photos of spores and cystidia as well as a distribution map of the collection sites in Greenland. Characters of each species are discussed and compared to related and similar species. Species ecology and distribution in Greenland and other regions (as appropriate) are discussed. Among these species is one species that is new to science and here described as *H. arcticum*. For another species, *H. colvinii*, originally described by Peck (1875; effectively published 1876), the first modern description is provided.

In spite of the amount of study that went into the genus, species identification, also by molecular data, remains challenging. Beker and co-workers used several loci (ITS, partial *RPB2*, partial *Tef1*a, partial *MCM7*, and the variable regions V6 and V9 of the mitochondrial genes). The sections mainly encountered in Greenland (*H.* sects. *Denudata*, *Hebeloma* and *Velutipes*) include a number of species that can only be distinguished by one of these loci, or by a combination of two loci or by combining molecular results and morphology. Also, a number of species are not monophyletic, even if all loci are concatenated. Although many species cannot be distinguished by ITS alone, the combination of ITS and morphology normally allows species identification (Beker et al. 2016).

Pruned median joining networks (Ayling and Brown 2008) are calculated to analyze the ITS results. Networks rather than trees are used to visualize DNA sequence variation when evolution has not been unidirectional. No assumptions are made with regard to which evolutionary mechanisms have been responsible for the observed variation. As in Cripps et al. (2019), it is not possible to unambiguously determine haplotypes from many sequences, i.e. when PCR was only successful in two fragments or when ambiguity in more than one single base pair position existed that was not accompanied by length variation. Therefore, we used as input for the network analysis what we refer to as “ITS variants”, for each collection, a consensus sequence of the ITS, whether or not intragenomic variation occurred. In the case of length variation, indels were treated as insertions.

### *Hebeloma* in Greenland and similar arctic (and alpine) areas

Lange (Lange 1957) collected in Greenland in 1946 and recorded four species: *Hebeloma mesophaeum*, *H. longicaudum*, *H. pusillum* and *H. strophosum*. His material has not been revised for this study, which is solely based on sequenced samples. Of his four species, we confirmed the presence of *H. mesophaeum*, but the three other named species are open to interpretation. *Hebeloma longicaudum* (Pers.) P. Kumm. is considered to be impossible to typify in accordance with the diagnosis. *Hebeloma pusillum* J.E. Lange is a species forming small basidiomes and was described from *Salix* scrublands in Denmark. According to Beker et al. (2016) this species does not occur in arctic and subarctic areas of northern Europe; the symbiont is not dwarf *Salix*, but rather larger *Salix* in bogs and fens, such as *S. aurita* L., *S. atrocinerea* Brot. and *S. cinerea* L. *Hebeloma strophosum* (Fr.) Sacc. is now (Beker et al. 2016) considered to be a synonym of *H. mesophaeum*.

Petersen (1977) also reported *H. mesophaeum* and *H. pusillum*, referring to Gulden and Lange (1971), and he added *H. crustuliniforme*, material, which is more likely to have represented *H. alpinum* or *H. velutipes*. Lamoure et al. (1982) reported *H. kuehneri* (= *H. nigellum*) from Disko Island, from where we have later also recorded it.

Watling (1977) reported *H. marginatulum* and *H. versipelle*, noting that the latter was probably included in Lange’s (1957) concept of *H. mesophaeum*. According to Beker et al. (2016), *H. versipelle* is probably synonymous with *H. mesophaeum*, although synonymy with *H. dunense* cannot be excluded. Watling (1983) reported H. aff. leucosarx, *H. marginatulum* and *H. mesophaeum*, revising collections from A. Erskine (NE Greenland, in the surroundings of Mestersvig airstrip) and A. Fox (Eqalummiut Nunaat). All of these species are here confirmed for Greenland, although *H. populinum* Romagn., mentioned by Watling as similar to his H. aff. leucosarx, is not and in fact is not known to occur north of the southern part of the British Isles. According to modern taxonomy, *H. populinum* is a member of H. sect. Denudata, and not a close relative of *H. leucosarx* which is a member of H. sect. Velutipes (Beker et al. 2016).

When Vesterholt (1989) revised the veiled species of *Hebeloma* in the Nordic countries, he included material from Greenland of *H. helodes*, *H. leucosarx* and *H. marginatulum*. Later Vesterholt (2005) added *H. alpinum*, *H. nigellum* (as *H. kuehneri*), *H. subconcolor* and *H. vaccinum* in his book “The Genus *Hebeloma*” and finally he added *H. dunense* (as *H. collariatum*). Two species reported in Borgen et al. (2006), *H. sinapizans* and *H. monticola*, are not among the material in this survey, and at least *H. sinapizans* is unlikely to be found. The collections ascribed to *H. sinapizans* may have represented *H. alpinum* or *H. geminatum*, the former in at least one case. Some, at least, of the collections of *H. monticola*, are now known to represent either *H. nigellum* or *H. oreophilum*, although it is likely that *H. monticola* may indeed be present in Greenland, particularly in the southern, subarctic areas.

Given the problems with interpretation of *Hebeloma* species names, in particular from the pre-molecular era, and therefore the application of taxon names, we will not make further reference to these studies. A small number of studies of other alpine or arctic regions have been published which use names in the same manner as here. Recently, Eberhardt et al. (2015a) described new species of *Hebeloma* from the alpine belt of the Carpathians. Beker et al. (2016) dedicated part of a chapter to alpine/arctic *Hebeloma*, identifying 17 species as ‘specialists’ and 8 species as ‘opportunists’ in these habitats in Europe. Beker et al. (2018) reviewed *Hebeloma* in Svalbard and recorded 17 species, of which five were only known from Svalbard. Cripps et al. (2019) described *Hebeloma* from the alpine Rocky Mountains in USA and found 16 species, of which one was subalpine and connected to conifers. Most recently, Grilli et al. (2020) presented seven species of *Hebeloma* from the Alps. This does not mean that, for example, we consider reports of *H. alpinum*, *H. marginatulum* or *H. mesophaeum* from various parts of the Russian Arctic or Alaska (Nezdoiminogo 1994; Miller 1998; Karatygin et al. 1999; Shiryaev et al. 2018; Gorbunova 2019) as unlikely, but we prefer to be consistent in referring only to material we have been able to validate.

### Bioclimatic zones and the collecting sites in Greenland

Greenland is an island, which for the greater part is covered by an ice cap, the Inland Ice. The island is biogeographically divided into four parts. South Greenland is the area south of 62.20°N. West Greenland is the area from 62.20°N to 74°N on the western side of the island. North Greenland is the area north of 74°N, and East Greenland the area on the eastern side between 74°N and 62.20°N.

**Figure 1.**
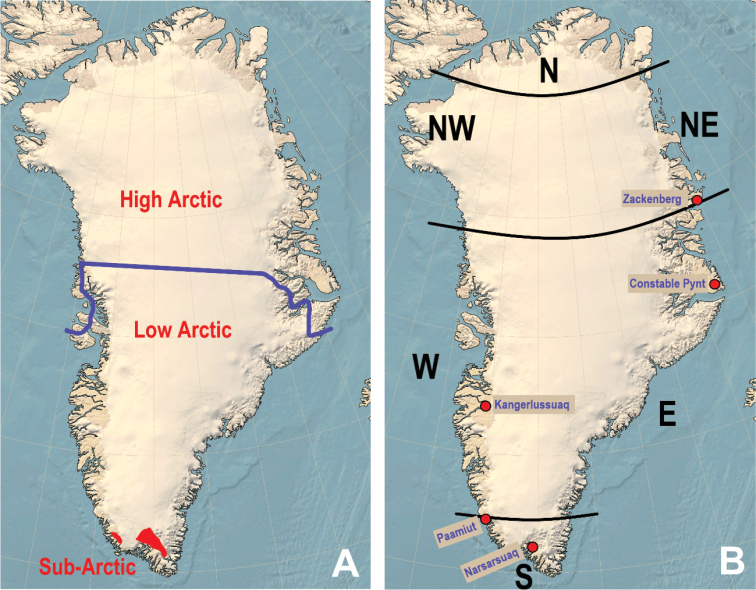
**A** Demarcation of Low and High Arctic, and **B** limits of North, East, West and South Greenland and location important collection sites.

The length of the island is ca. 2000 km, but only two bioclimatic zones are present, the Subarctic zone and the Arctic (or Polar) zone (Fredskild 1996). The Subarctic zone has a climate where the warmest month has an average temperature above 10 °C. This area is very small and restricted to the bottom of some of the fjords in South Greenland around Narsarsuaq. The outer part of the fjords is moist and cool, but in the protected inner parts, the temperature may rise during the summer to reach an average 10 °C for July. Botanically, this Subarctic zone is defined by the coincident occurrence of the two plants, *Sagina nodosa* and *Eleocharis quinqueflora*. The interest for *Hebeloma* lies in the occurrence of woodland with large specimens of Betula pubescens var. pumila (L.) Govaerts in a few valleys (Feilberg 1984), associated with a number of subarctic fungi (Elborne and Knudsen 1990). All other parts of Greenland are arctic. This is separated into the Low Arctic zone and the High Arctic zone. This separation (Fig. 1A) is important for the distribution of many organisms including fungi. The dividing line runs from the central part of Disko Island in the West across the Inland Ice to Blosseville Coast in the East, i.e. approximately along 70°N. The High Arctic zone has four months with an average temperature above 0 °C, whereas the Low Arctic zone has six months above 0 °C.

Collection sites are not distributed uniformly throughout Greenland since many areas are difficult to access. Collecting activities were concentrated on a few localities scattered over the accessible part of Greenland (Fig. 1B), taking into account the variation in the climate, soil and vegetation that defines the funga on this big island. A reflection of this is seen in the maps, where dots are always absent from some parts of Greenland, due to inaccessibility. This is especially true for NW Greenland (Melville Bay), where the Inland Ice comes out to the sea, leaving little opportunity for fungal life. The other area where records are generally missing is the southern half of the east coast, i.e. from the southernmost point in Greenland and up to Jameson Land on the East side. This is generally inaccessible, or extremely difficult to access, but a few collections were made by T.B. at Tasiilaq/Ammassalik. Finally, northernmost Greenland is an arctic desert, where the snowfall is generally too low to support fungi. Thus, our five main localities are Narsarsuaq, Paamiut, Kangerlussuaq, Zackenberg and Jameson Land.

#### Narsarsuaq at 60°N

A well-investigated area housing one of the two airports, which receives regular air traffic from Copenhagen. Generally a Low Arctic area, but at the bottom of some of the fjords the climate is Subarctic, and scrubland of Betula pubescens var. pumila occurs. The trunks attain heights up to 7–8 m and diameters to ca. 30 cm. Apart from these scrublands, occurring in a very restricted area, *Betula glandulosa* Michaux is widespread and common in the area. *Salix glauca* L. is ubiquitous, forming 1–3 m high shrubs, *S. arctophila* Cockerell and *S. herbacea* L. are common, while *S. uva-ursi* Pursh is rare and scattered. *Dryas integrifolia* M. Vahl is scattered. The soil is mixed, but generally rich in the valleys.

#### Paamiut at 62°N

This is the best studied area in Greenland for fungi as T.B. lived here for 20 years. The bedrock is acidic gneiss, in places with weathered basaltic dykes, and areas with less acid, syanitic “rotten” mountains. The climate is Low Arctic and hyper oceanic along the coast with heaths and snowbeds with *Salix herbacea*. Inland, *Betula glandulosa* heaths and copses of *Salix glauca* dominate. In addition to these widespread and common types of vegetation, a few scrubland areas of Alnus alnobetula subsp. crispa (Aiton) Raus. as well as *Dryas integrifolia* occur. *Betula pubescens* Erh. also grow here rarely, close to its northern limit.

#### Kangerlussuaq at 67°N

A well-studied area for the same reason as Narsarsuaq, in that the presence of an airport implies regular air traffic from Copenhagen. The area is Low Arctic and the bottom of the Kangerlussuaq Fjord (formerly Søndre Strømfjord) is the most continental area in Greenland, warm and dry in the summertime and even with salt lakes. The vegetation consists of low shrubs of *Betula nana* L., *Salix glauca*, *S. herbacea* and *S. arctophila*. *Dryas integrifolia* is common. The soil along the river and fjord is generally very rich due to loess coming from the Inland Ice with the rivers and with the wind, but the surrounding bedrock is acidic gneiss.

#### Zackenberg at 74.5°N

This area has been studied over the last 25 years, since the establishment of a field station in this High Arctic part of the Greenland National Park. Fungi were studied during two seasons by T.B. The shrub vegetation is *Betula nana*, *Salix arctica* Pall., *S. herbacea*, *Dryas integrifolia* and D. octopetala ssp. punctata (Juz.) Hult. The bedrock is gneissic or sedimental, mixed on the plains.

#### Constable Pynt, Jameson Land at 70.8°N

The area is situated on the border between Low Arctic and High Arctic and is accessible during summertime from Iceland. We studied the area in 1989 and in 2017. The bedrock is calcareous and the soil is generally nutrient rich. The vegetation is shrubs of *Betula nana*, *Salix arctica*, *S. glauca* and *Dryas octopetala* L.

## Materials and methods

### Collections

This study is based on 378 collections of *Hebeloma* from Greenland, which yielded (at least) ITS sequence data. Collections, which did not produce a sequence, are not considered further in this paper.

T.B., S.A.E. and H.K.K. collected 325 of the included collections over a period of 40 years. The remaining 56 collections were collected by Erik Rald (21), David Boertmann (9), Esteri Ohenoja (5, Herb. OULU), H.K.J.B. (4, HJB), Bent Fredskild (4), Egon Horak (2, Herb. ZT), Christian Bay (2), T.T. Elkington (2), Børge Lauritsen (2), Thomas Læssøe (2), Jens H. Petersen (1), Kuulo Kalamees (1) and Birger Knudsen (1). Many collections are provided with notes and photos. Unless otherwise mentioned, all material is kept at the Fungarium at the Natural History Museum of Denmark (C) in Copenhagen.

Photos were taken in the field with a camera or with an iphone, and T.B. also often took a laboratory photo of the fresh specimens. Collections were dried on an electrical heater whenever possible. In uninhabited areas without access to electricity, a system was used with three sieves fastened on aluminium “legs” and surrounded by a cylinder of wax cloth, creating a closed column. The “legs” were fastened to the ground, and beneath the sieves, a small kerosene lamp was placed to dry the material overnight.

### Molecular analyses

Dried collections were used as the source for genomic DNA. DNA extraction followed Cripps et al. (2019), with more recent material (i.e. younger than 20 years) omitting the pre-lysis incubation with lytic enzyme and omitting the lysis step overnight at 37 °C. Also, the time for DNA precipitation was shortened to 15–60 min.

PCR followed Cripps et al. (2019), but using the standard primer combination of ITS1F and ITS4 (White et al. 1990; Gardes and Bruns 1993) for more recent material. Partial *RPB2* PCR products (encoding the second largest subunit of RNA polymerase II; forward primer bRPB2-6f ‘TGG GGY ATG GTN TGY CCY GC’, Matheny 2005; and reverse fRPB2-7cr ‘CCC ATR GCT TGY TTR CCC AT’, Liu et al. 1999) were generated in 25 µl PCR reactions, using hotstart Taq polymerase, i.e. 1.25u Bioline (London, UK) MyTaq HS DNA Polymerase, 5 µl of 5× buffer, 20 pmol of each primer and 10% (2.5 µl) of 1:25 diluted DNA extract. PCRs were run with 5 min 95 °C, 40 cycles of 1 min 95 °C, 1 min 50 °C, 2 min 72 °C, and a final elongation of 5 min at 72 °C. The PCR cocktail for partial *Tef1*a, other than primers (translation elongation factor 1-α; forward primer elo31m ‘TTC ATC AAG AAC ATG ATC AC’ and reverse elo33R_A ‘GAC GTT GAA ACC RAC RTT GTC’, modified from Stielow et al. 2015) and Taq (MyTaq DNA polymerase, Bioline) was the same as for *RPB2*; PCRs were run as follows: 5 min 95 °C, 10 cycles of 45 sec 95 °C, 45 sec 58 °C, 1 min 72 °C, 35 cycles of 45 sec 95 °C, 45 sec 48 °C, 1 min 72 °C, and a final elongation of 5 min at 72 °C.

Sequencing was done by LGC Genomics (Berlin, Germany), using the PCR primers as sequencing primers. Sequences were edited using Sequencher (vs. 4.2 or 4.8). Newly generated sequences were submitted to GenBank (accession nos MW357874–MW357877, MW357892–MW357897, MW445544–MW445902, MW452577–MW452595, MW465762, MW465838 and MW465839). Other sequences used were previously published by Eberhardt et al. (2009, 2015a, b, 2016, 2018), Beker et al. (2010, 2013, 2016), Schoch et al. (2012), Grilli et al. (2016) and Cripps et al. 2019). Table 1 lists the GenBank accession numbers for the Greenland material treated in this paper.

**Table 1.** *Hebeloma* database references (see Beker et al. 2016), voucher information and Genbank accession numbers of Greenland collections considered in this study. For details, see Materials and Methods. Vouchers are from the fungal collection of the Herbarium of the University of Copenhagen (C), of the University of Oulu (OULU) or the collection of E. Horak at the herbarium of the Eidgenössische Technische Hochschule Zürich (ZT) or from the private collection of H.K.J. Beker.

*Hebeloma* Database reference	Voucher	Other Number	Genbank acc. no. ITS
***Hebeloma alpinicola***			
HJB15784	C-F-119805	TB99.238	MW445632
HJB16321	C-F-5081	TB85.071	MW445633
HJB16322	C-F-5082	TB85.099	MW445634
HJB16580	C-F-103554	TB81.111	MW445635
HJB16585	C-F-103532	TB85.218	MW445636
HJB16605	C-F-103516	TB00.049	MW445637
HJB16664	C-F-103534	TB84.063	MW445638
HJB16688	C-F-103559	TB84.028	MW445639
HJB17503	C-F-101623	TB08.039	MW445640
HJB17505	C-F-101621	TB08.037	MW445641
HJB17669	C-F-108401	HK16.165	MW445642
HJB18928	C-F-111109	HK18.010	MW445643
HJB18935	C-F-111116	HK18.322	MW445644
***Hebeloma alpinum***			
HJB11887	C-F-103458	TB86.115	MW445593
HJB12191	C-F-119742	TB99.027	MW445594
HJB12194	C-F-119744	TB99.023	KM390632, KM390633
HJB12204	C-F-104294	TB99.199	KM390685
HJB15711	C-F-119763	TB06.034	MW445544
HJB15714	C-F-119766	TB06.137	MW445545
HJB15785	C-F-119806	TB99.115	MW445548
HJB15786	C-F-119807	TB99.159	MW445549
HJB15787	C-F-119808	TB99.283	MW445550
HJB16276	C-F-101230	TB86.153	MW445551
HJB16581	C-F-103537	TB84.148	MW445552
HJB16591	C-F-103507	TB97.153a	MW445554
HJB16594	C-F-103506	TB97.152	MW445555
HJB16631	C-F-103565	TB86.141	MW445559
HJB16638	C-F-103503	TB95.004	MW445560
HJB17442	C-F-106779	TB17C.089	MW445564
HJB17445	C-F-106775	TB17C.053	MW445565
HJB17455	C-F-106784	TB17C.134	MW445566
HJB17458	C-F-105185	HK17.278	MW445567
HJB17459	C-F-104889	HK17.001	MW445568
HJB17461	C-F-105024	HK17.123	MW445569
HJB17467	C-F-104893	HK17.005	MW445570
HJB17470	C-F-105050	HK17.148	MW445571
HJB17475	C-F-104938	HK17.049	MW445595
HJB17476	C-F-104912	HK17.023	MW445572
HJB17477	C-F-104894	HK17.006	MW445573
HJB17482	C-F-104895	HK17.007	MW445575
HJB17486	C-F-104943	HK17.054	MW445576
HJB17491	C-F-106759	SAE-2017.014	MW445577
HJB17498	C-F-106758	SAE-2017.008	MW445579
HJB17509	C-F-106757	SAE-2017.006	MW445581
HJB17510	C-F-106766	SAE-2017.188	MW445582
HJB17663	C-F-104951	HK17.062	MW445586
HJB17687	C-F-5180	BF 90 loc. 6	MW445584
HJB18934	C-F-111115	HK18.308	MW445589
HJB18938	C-F-111119	HK18.390D	MW445591
***Hebeloma arcticum***			
HJB16618	C-F-103571	TB86.277A	MW445558
HJB16673	C-F-103555	TB90.083	MW445561
HJB16676	C-F-103483	TB90.071	MW445562
HJB16687	C-F-103584	TB16.095	MW445563
HJB17506	C-F-106751	TB08.153	MW445580
HJB17662	C-F-108472	SAE-2000.021-GR	MW445585
HJB17673	C-F-104149	HK16.119	MW445587
HJB17680	C-F-104080	HK16.044	MW445588
***Hebeloma aurantioumbrinum***			
HJB11884	C-F-119737	TB84.112	MW445897
HJB11885	C-F-2309	HK89.366	MW445896
HJB12189	C-F-119741	TB06.091	MW445899
HJB12205	C-F-119751	TB99.044	MW445898
HJB15716	C-F-119768	TB06.150	MW445858
HJB15719	C-F-119771	TB06.259	MW445859
HJB15740	C-F-119784	DB 85-17	MW357875†
HJB15741	C-F-119785	DB 85-28	MW357892‡
HJB15742	C-F-103459	TB85.239	MW445861
HJB15751	C-F-2327	SAE-89.121	MW445863
HJB15752	C-F-2424	SAE-89.430	MW445864
HJB15753	C-F-119787	DB GR88-22	MW445865
HJB15756	C-F-1461	SAE-88.149-GR	MW445866
HJB15757	C-F-119788	HK87.218	MW445867
HJB15758	C-F-119789	SAE-87.113-GR	MW445868
HJB15759	C-F-119790	HK87.004	MW445869
HJB15766	C-F-103462	TB84.135	MW445870
HJB15767	C-F-119792	TB84.150	MW445871
HJB15771	C-F-3637		MW445872
HJB15775	C-F-119797		MW445874
HJB16578	C-F-103461	TB84.132	MW445875
HJB16624	C-F-103502	TB93.070	MW445876
HJB16633	C-F-103570	TB86.251	MW445878
HJB16671	C-F-103484	TB90.072	MW445879
HJB16672	C-F-103546	TB90.039	MW445880
HJB16678	C-F-103481	TB90.041	MW445881
HJB16680	C-F-103545	TB90.029	MW445882
HJB16681	C-F-103482	TB90.057	MW445883
HJB16683	C-F-103547	TB90.012	MW445884
HJB16684	C-F-103548	TB90.011	MW445885
HJB16686	C-F-103485	TB90.133	MW445886
HJB16697	C-F-103526	TB85.217	MW445894
HJB17060	C-F-6992		MW445888
HJB17061	C-F-6993		MW445889
HJB17078	C-F-104315	ER93.262	MW445890
HJB17083	C-F-104321	ER93.091	MW445891
HJB17521	C-F-106745	SAE-2016.146	MW445892
HJB17674	C-F-104118	HK16.089	MW445893
HJB18933	C-F-111114	HK18.296	MW445895
HJB19596	C-F-103570	TB86.277B	MW445900
***Hebeloma clavulipes***			
HJB12316	C-F-119760	TB 90.100	MW357874†
***Hebeloma colvinii***			
HJB16630	C-F-103585	TB16.075	MW445745
HJB17502	C-F-106756	TB02.166	MW445746
HJB17684	C-F-104038	HK16.008	MW445747
HJB17685	C-F-104035	HK16.005	MW445748
HJB19653	C-F-107346	SAE-2016.188-GR	MW445749
***Hebeloma dunense***			
HJB12196	C-F-119746	TB99.114	MW445645
HJB12198	C-F-119748	TB99.411	MW445646
HJB12206	C-F-119752	TB99.219	MW445647
HJB15722	C-F-119774	TB06.159	MW445648
HJB15724	C-F-119776	TB06.263	MW445649
HJB16324	C-F-5087	TB86.159	MW445650
HJB16584	C-F-103530	TB85.200	MW445651
HJB16590	C-F-103486	TB91.045	MW445652
HJB16595	C-F-103536	TB84.114	MW445653
HJB16635	C-F-103563	TB86.177	MW445654
HJB16639	C-F-103589	TB85.183	MW445655
HJB16650	C-F-103527	TB85.186	MW445656
HJB16651	C-F-103535	TB84.090	MW445657
HJB17057	C-F-2561	JHP 89.259	MW445658
HJB17058	C-F-4216		MW445659
HJB17062	C-F-7017		MW445660
HJB17064	C-F-8231	HK15.078	MW445661
HJB17066	C-F-104293	TB16.076	MW445662
HJB17444	C-F-106782	TB17C.118	MW445663
HJB17449	C-F-106771	TB17C.010	MW445664
HJB17450	C-F-106770	TB17C.006	MW445665
HJB17452	C-F-106780	TB17C.094	MW445666
HJB17453	C-F-106773	TB17C.037	MW445667
HJB17456	C-F-106772	TB17C.030	MW445668
HJB17462	C-F-104959	HK17.070	MW445669
HJB17463	C-F-104932	HK17.043	MW445670
HJB17465	C-F-105171	HK17.265B	MW445671
HJB17466	C-F-105189	HK17.282	MW445672
HJB17471	C-F-104984	HK17.088	MW445673
HJB17474	C-F-104934	HK17.045	MW445674
HJB17481	C-F-105049	HK17.147	MW445675
HJB17483	C-F-105170	HK17.265A	MW445676
HJB17484	C-F-105108	HK17.203	MW445677
HJB17485	C-F-104941	HK17.052	MW445678
HJB17487	C-F-105028	HK17.127	MW445679
HJB17488	C-F-104945	HK17.056	MW445680
HJB17497	C-F-106765	SAE-2017.186	MW445681
HJB17507	C-F-106761	SAE-2017.103	MW445682
HJB17508	C-F-106769	SAE-2017.219	MW445683
HJB17678	C-F-104045	HK16.015	MW445684
HJB17688	C-F-6994		MW445685
HJB19155	C-F-7881	HK00-032	MW445686
***Hebeloma excedens***			
HJB13537	OULU F051033	EO19.8.00	MW445687
HJB16320	C-F-5073	TB85.238	MW445688
HJB16604	C-F-103517	TB00.086	MW445689
***Hebeloma fuscatum***			
HJB15739	C-F-119783	DB 85-21	MW445760
HJB17454	C-F-106783	TB17C.129	MW445787
HJB17473	C-F-104987	HK17.091	MW445789
HJB17478	C-F-104897	HK17.009A	MW445790
HJB17494	C-F-106768	SAE-2017.215	MW445791
HJB17517	C-F-106737	SAE-2016.072	MW445797
HJB18945	C-F-112530	TB18.243	MW445819
HJB18946	C-F-115623	DB 12.047	MW445820
***Hebeloma geminatum***			
HJB16588	C-F-103508	TB97.154a	MW445553
HJB16596	C-F-103514	TB00.065	MW445556
HJB18936	C-F-111117	HK18.379B	MW445590
***Hebeloma grandisporum***			
HJB17067	C-F-104295	TB99.376	MW445784
HJB17460	C-F-104997	HK17.101	MW445788
***Hebeloma helodes***			
HJB15747	C-F-103460	TB85.072	MW445862
HJB15748	C-F-103476	TB85.090	MW357894‡
HJB15780	C-F-4003	TB88.114	MW445873
HJB16627	C-F-103525	TB85.065	MW445877
HJB17044	C-F-104317	ER93.153	MW445887
***Hebeloma hiemale***			
HJB12193	C-F-119743	TB06.067	GQ869517
HJB12195	C-F-119745	TB99.258	GQ869515
HJB12200	C-F-104296	TB99.146	GQ869524
HJB12202	C-F-119777,	TB06.081	GQ869518
HJB12210	C-F-119756	TB99.118	GQ869516
HJB12544	Coll. E. Horak at ZT 8901		GQ869527
HJB13538	OULU F050202	EO12.8.00	MW445631
HJB15712	C-F-119764	TB06.250	MW445596
HJB15713	C-F-119765	TB06.120	MW445597
HJB15715	C-F-119767	TB06.128	MW445598
HJB15717	C-F-119769	TB06.061	MW445599
HJB15718	C-F-119770	TB06.033	MW445600
HJB15736	C-F-103465	TB91.136	MW445601
HJB15737	C-F-119782	TB91.112	MW445602
HJB15743	C-F-103466	TB85.182	MW445603
HJB15746	C-F-119786	TB85.250	MW357893‡
HJB15770	C-F-119794	TL 84.608	MW357895‡
HJB15781	C-F-119802	TB99.280	MW445604
HJB15782	C-F-119803	TB99.304	MW445605
HJB15788	C-F-119809	TB99.160	MW445606
HJB16600	C-F-103515	TB00.069	MW445607
HJB16616	C-F-103497	TB93.183	MW445608
HJB16619	C-F-103499	TB93.210	MW445609
HJB16621	C-F-103498	TB93.187	MW445610
HJB16622	C-F-103500	TB93.155	MW445611
HJB16628	C-F-103552	TB81.112	MW445612
HJB16636	C-F-103496	TB93.159	MW445613
HJB16659	C-F-103543	TB90.032	MW445614
HJB16666	C-F-103540	TB90.087	MW445615
HJB16667	C-F-103549	TB90.084	MW445616
HJB16674	C-F-103544	TB90.019	MW445617
HJB16675	C-F-103568	TB86.203	MW445618
HJB16685	C-F-103550	TB90.104a	MW445619
HJB16692	C-F-103504	TB95.114	MW445620
HJB17042	C-F-104550	ER93.330	MW445621
HJB17043	C-F-104292	TB98.201	MW445622
HJB17045	C-F-104551	ER93.302	MW445623
HJB17080	C-F-104318	ER93.152	MW445624
HJB17443	C-F-106776	TB17C.072	MW445625
HJB17446	C-F-106777	TB17C.078	MW445626
HJB17501	C-F-106752	TB08.157	MW445627
HJB18931	C-F-111112	HK18.269	MW445628
HJB18941	C-F-112771	SAE-2018.225-GR	MW445629
HJB18942	C-F-112904	SAE-2018.357-GR	MW445630
***Hebeloma hygrophilum***			
HJB16647	C-F-103511	TB98.234	MW357897‡
HJB17041	C-F-104549	ER93.425	MW445783
HJB17516	C-F-106736	SAE-2016.022	MW445796
HJB17520	C-F-106735	SAE-2016.005	MW445799
HJB17522	C-F-106741	SAE-2016.105	MW445800
HJB17523	C-F-106746	SAE-2016.168	MW445801
HJB17524	C-F-106742	SAE-2016.116	MW445802
HJB17661	C-F-108600	SAE-2000.148-GR	MW445803
HJB17667	C-F-108446	HK16.195	MW445804
HJB17681	C-F-104093	HK16.064	MW445811
HJB18937	C-F-111118	HK18.390A	MW445815
HJB18943	C-F-115622	SAE-2018.429-GR	MW445817
HJB18944	C-F-112528	TB18.236	MW445818
HJB19151	C-F-105494	ER93.519	MW445813
HJB19710	C-F-137115	TB19.052	MW445821
***Hebeloma ingratum***			
HJB10797	C-F-119732	HK87.262	KT217437
HJB13546	OULU F050503	EO18.8.00.36	MW445837
HJB16620	C-F-103501	TB93.205	MW445826
HJB17513	C-F-106748	SAE-2016.208	MW445831
***Hebeloma islandicum***			
HJB16632	C-F-103573	TB86.291	MW445901
***Hebeloma leucosarx***			
HJB16656	C-F-103513	TB98.119	MW445844
HJB16658	C-F-103551	TB81.211	MW445845
***Hebeloma louiseae***			
HJB16602	C-F-103518	TB00.061	MW445557
HJB17479	C-F-105029	HK17.128	MW445574
HJB17493	C-F-106763	SAE-2017.125A	MW445578
HJB17519	C-F-106747	SAE-2016.197	MW445583
HJB19601	C-F-106763	SAE-2017.125B	MW445592
***Hebeloma marginatulum***			
HJB10730	Priv. coll. HJB10730		MW445690
HJB10732	Priv. coll. HJB10732		MW445691
HJB10739	Priv. coll. HJB10739		MW445692
HJB10742	Priv. coll. HJB10742		MW445693
HJB12197	C-F-119747	TB06.090	MW445694
HJB15723	C-F-119775	TB06.158	MW445695
HJB15727	C-F-119779	TB06.090	MW445696
HJB15762	C-F-103467	TB86.247	MW445697
HJB16323	C-F-5085	TB85.036	MW445698
HJB16327	C-F-5090	TB86.072	MW445699
HJB16612	C-F-103489	TB91.198	MW445700
HJB16623	C-F-103495	TB93.139	MW445701
HJB16646	C-F-103566	TB86.119	MW445702
HJB16652	C-F-103493	TB92.027	MW445703
HJB16654	C-F-103492	TB92.028	MW445704
HJB16663	C-F-103553	TB81.109	MW445705
HJB16665	C-F-103538	TB84.151	MW445706
HJB16691	C-F-103505	TB95.068	MW445707
HJB17049	C-F-104552	ER93.320	MW445708
HJB17050	C-F-104300	TB86.299	MW445709
HJB17051	C-F-104306	ER92.181	MW445710
HJB17052	C-F-104314	ER93.181	MW445711
HJB17059	C-F-6922	TB86.060	MW445712
HJB17070	C-F-104304	ER92.109	MW445713
HJB17071	C-F-104305	ER92.180	MW445714
HJB17077	C-F-104312	ER93.111	MW445715
HJB17081	C-F-104319	ER93.021	MW445716
HJB17086	C-F-104554	ER93.589	MW445717
HJB17457	C-F-106421	ER93.112	MW445718
HJB17489	C-F-105051	HK17.149	MW445719
HJB17490	C-F-106738	SAE-2016.085	MW445720
HJB17499	C-F-106749	TB08.126	MW445721
HJB17500	C-F-106753	TB09K017	MW445722
HJB17525	C-F-101622	TB08.035	MW445723
HJB17526	C-F-106750	TB08.146	MW445724
HJB17658	C-F-108419	HK16.181	MW445725
HJB17666	C-F-108418	HK16.180	MW445726
HJB17677	C-F-104164	HK16.134	MW445727
HJB17683	C-F-104111	HK16.082	MW445728
HJB17686	C-F-5113	BF 90 loc. 5	MW445729
HJB17689	C-F-5147	BF 90 loc. 8	MW445730
HJB18932	C-F-111113	HK18.288	MW445731
***Hebeloma mesophaeum***			
HJB12213	C-F-104297	TB99.264	MW445735
HJB12313	C-F-119759	TB90.040	MW465762
HJB13560	OULU F050224	EO12.8.00.1	MW445736
HJB16348	C-F-76757	TB90.086	MW445732
HJB16598	C-F-103521	TB00.093	MW445737
HJB16601	C-F-103522	TB00.094	MW445738
HJB16603	C-F-103520	TB00.091	MW445739
HJB16629	C-F-103578	TB16.040G	MW445740
HJB17068	C-F-104301	TB16.038	MW445741
HJB17464	C-F-104908	HK17.020	MW445733
HJB19682	C-F-137116	TB00.088	MW445734
***Hebeloma minus***			
HJB15745	C-F-104302	TB86.085	MW445546
HJB15769	C-F-119793	TL 84.041	MW445547
***Hebeloma nigellum***			
HJB10957	C-F-119734	DB GR83-80	MW445750
HJB11874	C-F-103468	TB84.183	MW445752
HJB11888	C-F-119739	TB86.052	MW445751
HJB12545	Coll. E. Horak at ZT 9139		MW445756
HJB13559	OULU F050653	EO19.8.00.20	MW445758
HJB15761	C-F-119791	TB86.065	MW445761
HJB16592	C-F-103577	TB16.035G	MW445764
HJB16606	C-F-103490	TB91.080	MW445766
HJB16634	C-F-103572	TB86.276	MW445769
HJB16657	C-F-103529	TB85.249	MW445773
HJB16670	C-F-103479	TB90.035	MW445776
HJB16677	C-F-103541	TB90.073	MW445777
HJB16679	C-F-103542	TB90.056	MW445778
HJB16694	C-F-103583	TB16.086G	MW445781
HJB16696	C-F-103580	TB16.060G	MW445782
HJB17518	C-F-106743	SAE-2016.131	MW445798
HJB17670	C-F-108402	HK16.165A	MW445805
HJB17675	C-F-104140	HK16.110	MW445808
HJB17676	C-F-104163	HK16.133	MW445809
HJB17679	C-F-104063	HK16.033	MW445810
HJB18929	C-F-111110	HK18.199	MW445814
***Hebeloma oreophilum***			
HJB11889	C-F-119740	TB85.180	MW445755
HJB12199	C-F-119772	TB06.225	MW445757
HJB12212	C-F-119758	TB99.374	MW445753
HJB15721	C-F-119773	TB06.098	MW445759
HJB15768	C-F-104298	TB84.215	MW445762
HJB16589	C-F-103539	TB84.184	MW445763
HJB16599	C-F-103576	TB16.017G	MW445765
HJB16611	C-F-103487	TB91.233	MW445767
HJB16613	C-F-103588	TB86.292	MW445768
HJB16644	C-F-103510	TB98.120	MW445770
HJB16645	C-F-103509	TB98.073	MW445771
HJB16648	C-F-103590	TB85.061	MW445772
HJB16668	C-F-103472	TB83.035	MW445774
HJB16669	C-F-103558	TB90.010	MW445775
HJB16682	C-F-103480	TB90.036	MW445779
HJB17515	C-F-106744	SAE-2016.134	MW445795
HJB17672	C-F-104120	HK16.091	MW445807
HJB18939	C-F-111120	HK18.401	MW445816
***Hebeloma pubescens***			
HJB12203	C-F-119750	TB99.194	MW445742
HJB12207	C-F-119753	TB99.305	MW445743
HJB16326	C-F-5089	TB86.128	MW445744
***Hebeloma spetsbergense***			
HJB10869	C-F-119733	SAE-1986.135-GR	MW445822
HJB12211	C-F-119757	TB99.256	MW445754
HJB15763	C-F-103478	TB86.121	MW357896‡
HJB15779	C-F-119801	DB 83-62	MW357877†
HJB16693	C-F-103579	TB16.056G	MW445780
HJB17447	C-F-106781	TB17C.113	MW445785
HJB17448	C-F-106774	TB17C.050	MW445786
HJB17495	C-F-106764	SAE-2017.174	MW445792
HJB17496	C-F-106760	SAE-2017.043	MW445793
HJB17514	C-F-106740	SAE-2016.102	MW445794
HJB17671	C-F-108450		MW445806
HJB17682	C-F-104100	HK16.071	MW445812
***Hebeloma subconcolor***			
HJB12413	C-F-119761	TB90.018	KT218391
HJB15750	C-F-2195	HK89.302	MW445840
HJB15760	C-F-4002	TB87.117	MW445841
HJB16579	C-F-103587	TB86.122	MW445902
HJB17055	C-F-104299	TB90.033	MW445848
HJB17056	C-F-104313	ER93.168	MW445849
HJB17065	C-F-8242	HK15.089	MW445847
HJB17512	C-F-106739	SAE-2016.090	MW445852
HJB18930	C-F-111111	HK18.232	MW445855
***Hebeloma vaccinum***			
HJB12190	C-F-119780,	TB06.246	KT217493
HJB12209	C-F-119755	TB99.109	KT217494
HJB15749	C-F-103477	TB85.045	MW357876†
HJB15783	C-F-119804	TB99.008	MW445838
HJB17063	C-F-8222	HK15.069	MW445833
HJB17073	C-F-104308	ER92.038	MW445834
HJB17076	C-F-104311	ER92.323	MW445835
HJB17082	C-F-104320	ER93.022	MW445836
HJB17451	C-F-106778	TB17C.073	MW445827
HJB17469	C-F-104909	HK17.021	MW445828
HJB17472	C-F-104910	HK17.022A	MW445829
HJB17492	C-F-106767	SAE-2017.194	MW445830
HJB17664	C-F-104911	HK17.022B	MW445832
***Hebeloma velutipes***			
HJB12201	C-F-119749	TB99.336	MW445857
HJB12208	C-F-119754	TB99.309	MW445856
HJB15730	C-F-103557	TB86.179	MW445839
HJB16597	C-F-103519	TB00.073	MW445842
HJB16655	C-F-103512	TB98.158	MW445843
HJB16689	C-F-103582	TB16.087G	MW445846
HJB17468	C-F-105090	HK17.186	MW445850
HJB17511	C-F-106762	SAE-2017.110	MW445851
HJB17659	C-F-108492	SAE-2000.041-GR	MW445853
HJB17660	C-F-108502	SAE-2000.051-GR	MW445854

† ITS1 only; ‡ ITS2 only.

Sequences were aligned in Mafft v. 7 online (https://mafft.cbrc.jp/alignment/server/, Katoh et al. 2019), using the G-INS-i method for ITS sequences for networks and the E-INS-i for ITS, *RPB2* and *Tef1*a data for the tree analysis.

Following Cripps et al. (2019), pruned quasi-median joining networks (Ayling and Brown 2008) were used to visualize the biological diversity of ITS sequences generated from Greenland collections. In the networks, observed sequence variants are shown as circles and the size of each circle gives some indication of the number of times the respective sequence variant has been retrieved. Two circles connected by an unsegmented line differ in 1 bp. So-called quasimedians, a kind of placeholder for unobserved sequence variants depicted as small squares, are placed between observed sequence variants that each differ from the quasi-median by 1 bp. The number of segments to a line represents the number of base pair changes between two sequence variants or a sequence variant and a quasi-median. A pruning mechanism is applied to reduce the complexity of the networks while depicting at least one shortest path between all pairs of sequence variants (Ayling and Brown 2008).

Only complete ITS sequences were considered. The Greenland sequences were incorporated in alignments previously published by Cripps et al. (2019). *Hebeloma ingratum* was not encountered by Cripps et al. (2019), but is present in the Greenland sample. Thus the *H. cavipes*/*H. vaccinum* dataset was extended by 10 randomly selected *H. ingratum* sequences from Europe. Because of the large number of sequences, and because there was no overlap between *H. marginatulum* and the other members of the species group around *H. mesophaeum*, *H. marginatulum* sequences were analyzed in a separate analysis. *Hebeloma incarnatulum*, a boreal species included by Cripps et al. (2019) in the H. sect. Velutipes network, was excluded from the analysis as it had no corresponding data in the Greenland sample. Alignments are published in TreeBase (Study ID TB2:S27569), where GenBank acc. numbers for all sequences used are given.

Networks were calculated in SplitsTree (version 4.15.1, Huson and Bryant 2006) with the default options other than activating the ‘scale nodes by taxa’ and ‘subdivide edges’ options. Nodes representing different classes of sequences (differentiated by species and origin, ‘Greenland’ versus ‘outside Greenland’) were replaced in Adobe Illustrator CS6 by pie charts of corresponding diameters, showing the relative numbers of sequences for each class.

For showing the phylogenetic placement of the newly described species *H. arcticum*, a tree analysis of members of H. subsect. Crustuliniformia was included, based on concatenated data of ITS, *RPB2* and *Tef1*a. Table 2 lists the respective GenBank asscion numbers. Tree analyses were done locally in RAxML (Stamatakis 2014) using raxmlGUI v. 2.0 (Edler et al. 2020), using the “ML + rapid bootstrap” option, determining the number of bootstrap replicates by the autoMRE method. Prior to concatenation, single locus trees (see Treebase submission) were generated. No conflicts were detected using the principle by Kauff and Lutzoni (2002), assuming a conflict to be significant if two different relationships for the same set of taxa, one being monophyletic and the other non-monophyletic, are supported by bootstrap with more than 70% in ML analyses. Based on the results of Eberhardt et al. (2015b), the data were analyzed unpartitioned. *Hebeloma louiseae* was used for rooting following Beker et al. (2016).

**Table 2.** *Hebeloma* database references (see Beker et al. 2016), country, voucher and GenBank accession numbers of sequences used for the ML analysis (Fig. 5B). BR-MYCO, C-F and L refer to the fungal collections of the Meise Botanic Garden (BR), the Herbarium of the University of Copenhagen (C), or Naturalis, Leiden (L), respectively. Vouchers with numbers starting with AdH, AT, or HJB are from the private collections of A.F.S. Taylor, A. de Haan or H.K.J. Beker.

*Hebeloma* database reference	Country	Voucher	ITS GenBank acc. no.	*Tef1a* GenBank acc. no.	*Rpb2* GenBank acc. no.
***Hebeloma aanenii***					
HJB10282	Belgium	HJB10282	JN943877	KT216789	KF309471
HJB10670	Sweden	AT2003063	KM390550	KT216808	KM390126
HJB12630 holotype	Poland	BR-MYCO 173987-66	KM390723	KT216852	KM390169
***Hebeloma alpinum***					
HJB11051	Iceland	HJB11051	JN943865	MW452578	KF309496
HJB11094	Switzerland	HJB11051	KM390594 KM390595	KT216817	KM390142
HJB11117	Switzerland	HJB11051	KM390593	KT216819	KM390141
***Hebeloma arcticum***					
HJB16687	Greenland	C-F-103584	MW445563	MW452581	MW452590
HJB17506	Greenland	C-F-106751	MW445580	MW452583	MW452592
HJB17673 holotype	Greenland	C-F-104149	MW445587	MW452584	MW452593
HJB17680	Greenland	C-F-104080	MW445588	MW452585	MW452594
***Hebeloma aurantioumbrinum***					
HJB12058 holotype	Norway, Svalbard	BR-MYCO 173985-64	KM390686 KM390687	KT216845	KM390158
HJB12445	U.S.A.	HJB12445	KM390714 KM390715	KT216851	KM390166
HJB12451	U.S.A.	HJB12451	KM390720 KM390721	MW452577	KM390168
***Hebeloma crustuliniforme***					
HJB11237	Spain		JN943870	KT216824	KF309480
HJB12807	Netherlands	L WBS 9581	KF309415	KT216854	KF309492
HJB13713 epitype	France	BR-MYCO 173989-68	KF309424	KT216860	KF309495
***Hebeloma eburneum***					
HJB9267	U.K., England	HJB9267	JN943880	KT216777	KF309468
HJB10290	Belgium	HJB10290	KM390533 KM390534	KT216792	KM390113
HJB12670	Poland	HJB12670	KM390727 KM390728	KT216853	KM390171
***Hebeloma geminatum***					
HJB8633	Belgium	HJB8633	KM390526	KT216769	KM390109
HJB10384	U.K., England	HJB10384	KF309400	KT216794	KF309472
HJB10833 holotype	Denmark	C-F-90152	KF309405	KT216815	KF309478
***Hebeloma helodes***					
HJB8115	U.K., Wales	HJB8115	KM390527	KT216768	KM390110
HJB10680	U.K., England	HJB10680	KM390548	KT216810	KM390124
HJB11698	France	HJB11698	KM390622	KT216831	KM390151
***Hebeloma louiseae***					
HJB11984	Norway, Svalbard	HJB11984	KM390705	KT216839	KM390164
HJB16602	Greenland	C-F-103518	MW445557	MW452580	MW452589
HJB19601	Greenland	C-F-106763	MW445592	MW452586	MW452595
***Hebeloma luteicystidiatum***					
HJB11837 holotype	Belgium	BR-MYCO 166233-72	KM390624	KT216837	KM390152
HJB12174	Belgium	HJB12174	KM390710		KT216978
HJB16715	France	HJB EG-151028.02	MW465839	MW452582	MW452591
***Hebeloma lutense***					
HJB 9819	U.K., Scotland		JN943871	KT216783	KF309479
HJB 10523	Belgium	AdH04059	KM390541	KT216798	KM390119
HJB11328	France	HJB11328	JN943864	KT216825	KF309486
***Hebeloma matritense***					
HJB9485 holotype	Spain	BR-MYCO 174910-19	KT217364	KT216781	KT216879
HJB9486	Spain	HJB9486	KT217365	KT216782	KT216880
***Hebeloma minus***					
HJB11079	Iceland	HJB11079	JN943866	KT216816	KF309481
HJB11107	Switzerland	HJB11107	JN943868	KT216818	KF309483
HJB12007	Norway, Svalbard	HJB12007	JN943857	KT216842	KF309488
***Hebeloma pallidolabiatum***					
HJB11992 holotype	Norway, Svalbard	BR-MYCO 174908-17	KM390702 KM390703	KT216840	KM390163
HJB12059	Norway, Svalbard	HJB12059	KM390713	KT216846	MW452587
***Hebeloma perexiguum***					
HJB12038 holotype	Norway, Svalbard	BR-MYCO 173979-58	KM390689	KT216844	KM390160
***Hebeloma pusillum***					
HJB9494	U.K., Wales	HJB9494	KM390530		KM390111
HJB11728	Belgium	HJB11728	JN943862	KT216835	KF309497
HJB14747	U.K., Isle of Man	HJB14747	MW465838	MW452579	MW452588
***Hebeloma salicicola***					
HJB10260	Belgium	HJB10260	KF309403	KT216788	KF309470
HJB10422	Belgium	HJB10422	JN943878	KT216795	KF309473
HJB 13302 holotype	Belgium	BR-MYCO 173977-56	KM390683	KT216857	KM390177

### Morphological methods

All microscopical analysis was carried out on dried material, using a Leica DMRZA2 microscope with a Leica DFC495 camera connected to a computer running Leica Application Suite (LAS) V4 software. The spores were first studied in Melzer’s reagent to assess the shape, degree of dextrinoidity, ornamentation and the degree of loosening of the perispore. For the assessment of the degrees of ornamentation (O0, O1, O2, O3, O4), of the loosening perispore (P0, P1, P2, P3) and for the dextrinoidity (D0, D1, D2, D3, D4), we used Beker et al. (2016), where more details can be found.

A number of photographs were taken of the spores at x500 and x1600, which were then measured using the LAS software. Wherever possible, for each collection at least 50 spores were measured in Melzer’s reagent, excluding the apiculi. The maximum length and width of each spore was measured, and its Q value (ratio of length to width) calculated. Average length, width, and Q value were calculated and recorded, as well as the median, standard deviation, and 5% and 95% percentiles. Photographs were also taken of the cheilocystidia on the lamella edge at x500 and of individual cystidia and basidia at x1000. The material was then examined in 5% KOH. Again, photographs were taken of the spores at x500 and x1600 and of the cheilocystidia (and pleurocystidia if any were present) and basidia at x500 and x1000.

For the cheilocystidia, the average width of the widest part of the cheilocystidium in the vicinity of the apex appears to be an important character in the separation of species within *Hebeloma* (Vesterholt 2005). It is also important, when determining this average width near the apex, not to be selective with regard to the cystidia chosen for measurement. To determine the average width at the apex about 100 cheilocystidia were measured on the lamellae edge. For other measurements, at least 20 cheilocystidia, wherever possible, separated from the lamella edge, were measured from each collection. Because of the complex shapes of the cheilocystidia four measurements were made: length, width at apex (A), width at narrowest point in central region (M), and maximum width in lower half (B). The measurements were given in this order, and an average value was calculated for each of these measurements. For each cheilocystidium the ratios A/M, A/B, and B/M were calculated and averaged across all cheilocystidia measured. Separate mountings of the material were used, where possible, to examine the caulocystidia and pileipellis.

Morphological terms, including colors, are as in Beker et al. (2016), which we have used as the basis for the descriptions, emending some of them where new data from new collections became available.

## Results

**Figure 2.**
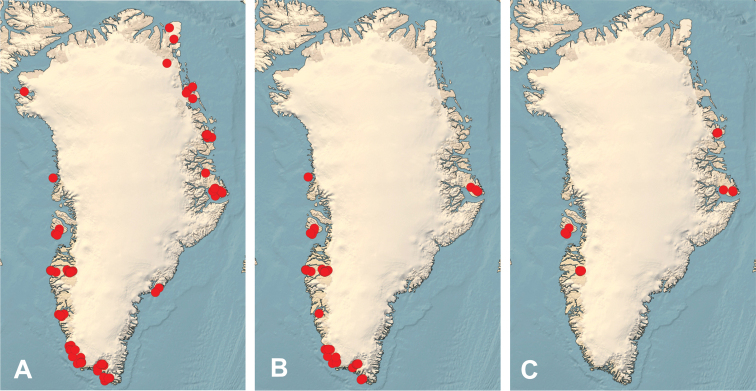
*Hebeloma* species collecting sites in Greenland **A** all **B** collection sites in the Low Arctic, encompassing groups 1–3, and **C** in the High Arctic, encompassing group 5.

Of the 405 collections studied, sequence data was generated from 378, which formed the basis of this study. The overall distribution of the sample is shown in Fig. 2A, but the main collection sites starting with the most southerly were Narsarsuaq, Paamiut, Kangerlussuaq, Zackenberg and Jameson Land.

The combined morphological and molecular analysis determined the presence of 28 species, of which one, *H. arcticum*, is described as new to science. For another, *H. colvinii*, the first modern description is provided. The others are *H. alpinicola*, *H. alpinum*, *H. aurantioumbrinum*, *H. clavulipes*, *H. dunense*, *H. excedens*, *H. fuscatum*, *H. geminatum*, *H. grandisporum*, *H. helodes*, *H. hiemale*, *H. hygrophilum*, *H. ingratum*, *H. islandicum*, *H. leucosarx*, *H. louiseae*, *H. marginatulum*, *H. mesophaeum*, *H. minus*, *H. nigellum*, *H. oreophilum*, *H. pubescens*, *H. spetsbergense*, *H. subconcolor*, *H. vaccinum* and *H. velutipes*. Nineteen of these taxa are new records for Greenland and six are new records for the North American continent: *H. clavulipes*, *H. grandisporum*, *H. islandicum*, *H. louiseae*, *H. minus* and *H. pubescens*. Three species, *H. arcticum*, *H. colvinii* and *H. excedens* have not been recorded from Europe.

### Geographical distribution of *Hebeloma* species in the sample

The 28 species all occur in the Arctic zone. In the small Subarctic zone in southernmost Greenland (< 61.25°N), 13 species were recorded, but they also occurred in the Arctic zone. Below, the species are grouped according to the regions within which they occur; Table 3 lists the species in each group.

**Table 3.** *Hebeloma* species composition of the Greenland sample by species distribution groups (see main text). Classification into “specialist” and “opportunist” taxa follows Beker et al. (2016).

Species by region	No. of collections	Specialist (S) or opportunist (O)
**Group 1 – South Greenland (60.9–62°N)**		
*H. clavulipes*	1	O
*H. helodes*	5	O
*H. islandicum*	1	S
*H. leucosarx*	2	O
**Group 2 – South & West Greenland (60.2–69.3°N)**		
*H. arcticum*	8	S
*H. colvinii*	5	O
*H. excedens*	3	O
*H. geminatum*	3	O
*H. hygrophilum*	15	O
*H. ingratum*	4	O
*H. minus*	2	S
**Group 3 – South, West & East Greenland (60.1–72.8°N)**		
*H. fuscatum*	8	S
*H. nigellum*	21	S
*H. subconcolor*	9	S
**Group 4 – All of Greenland (60.1–81.6°N)**		
*H. alpinicola*	13	S
*H. alpinum*	36	S
*H. aurantioumbrinum*	40	S
*H. dunense*	42	S
*H. hiemale*	44	O
*H. marginatulum*	42	S
*H. mesophaeum*	11	S
*H. oreophilum*	18	S
*H. vaccinum*	13	O
*H. velutipes*	10	O
**Group 5 – Never collected in South Greenland (67–74.5°N)**		
*H. grandisporum*	2	S
*H. louiseae*	5	S
*H. pubescens*	3	S
*H. spetsbergense*	12	S
**Total**	**378**

- Group 1. Four species appear to have a southerly distribution and are only known from South Greenland (< 62.2°N): H. clavulipes, H. helodes, H. islandicum and H. leucosarx.
- Group 2. Seven species appear to have a southwesterly distribution, i.e. are known from just southern and western Greenland (< 69.25°N): H. arcticum, H. colvinii, H. excedens, H. ingratum, H. geminatum, H. hygrophilum and H. minus.
- Group 3. Only three species have been collected in southwestern and south-eastern Greenland but not northern Greenland (< 67°N): H. fuscatum, H. nigellum and H. subconcolor.
- Group 4. Ten species have been collected all over Greenland in Subarctic, Low Arctic and High Arctic zones: H. alpinicola, H. alpinum, H. aurantioumbrinum, H. dunense, H. hiemale, H. marginatulum, H. mesophaeum, H. oreophilum (not collected in east Greenland), H. vaccinum (not collected in west Greenland), and H. velutipes.
- Group 5. Four species are northern and have never been collected in south Greenland (> 67°N). They have only been verified for the High Arctic zone: H. grandisporum, H. louiseae, H. pubescens and H. spetsbergense.

Fourteen species are only found in the southern half of Greenland (groups 1, 2 and 3; Table 3 and Fig. 2B, the Subarctic and Low Arctic zones defined by at least six months with an average temperature above zero: *H. arcticum*, *H. clavulipes*, *H. colvinii*, *H. excedens*, *H. fuscatum*, *H. geminatum*, *H. helodes*, *H. hygrophilum*, *H. ingratum*, *H. islandicum*, *H. leucosarx*, *H. minus*, *H. nigellum* and *H. subconcolor.* Four species have never been recorded from south Greenland. This is Group 5, above and see also Table 3 and Fig. 2C.

### Molecular results

Full ITS sequence data was obtained from 367 Greenland collections; 10 collections, indicated in Table 1, only yielded either ITS1 or ITS2 sequences. These sequences were compared to existing sequence data and supported the morphological identifications. They are not included in the molecular analyses.

**Figure 3.**
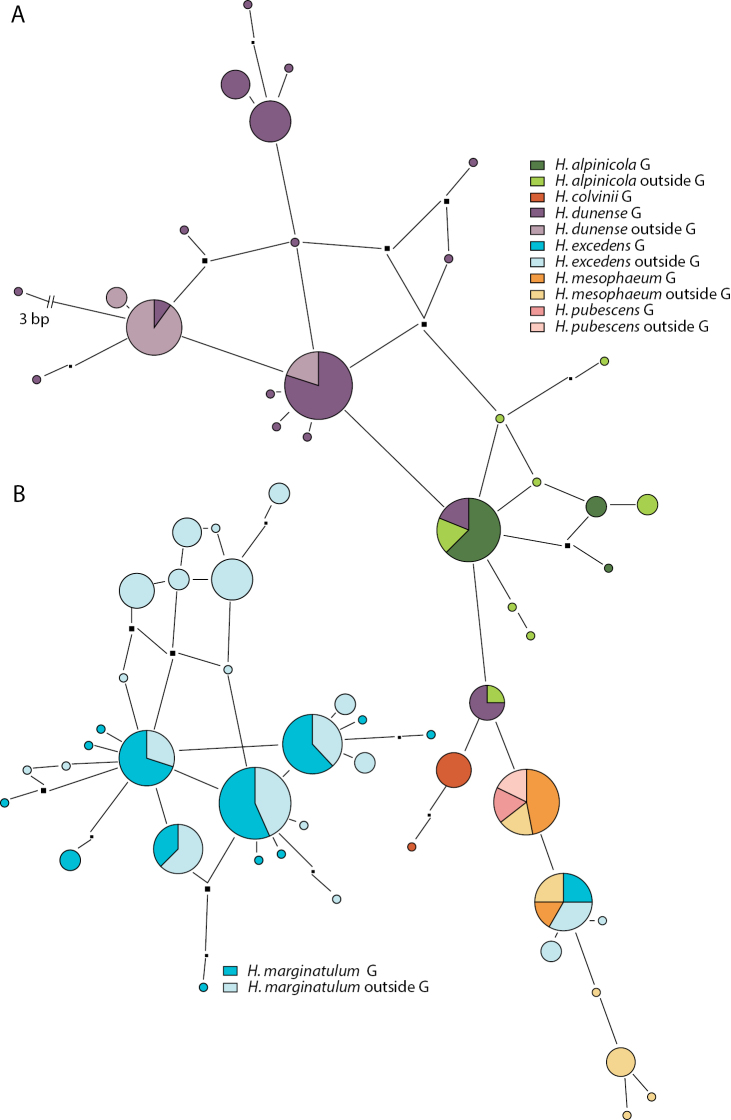
Pruned quasi-median networks of **A** the *Hebeloma mesophaeum* complex and **B***Hebeloma marginatulum*. Circles shared by two or more taxa or collections from two regions, from Greenland (G) or collected elsewhere, are divided according to the number of representatives for each class.

**Figure 4.**
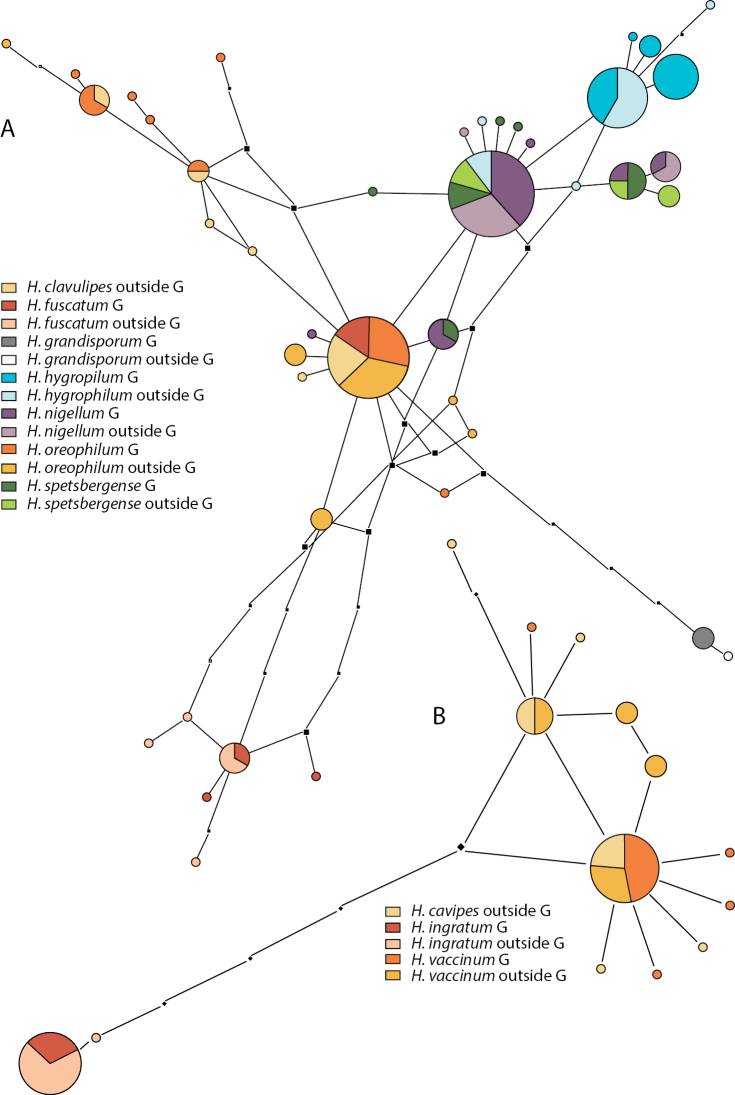
Pruned quasi-median network of **A** the *Hebeloma nigellum* complex and *H. grandisporum* and **B***Hebeloma cavipes*, *H. ingratum* and *H. vaccinum*. Circles shared by two or more taxa or collections from two regions, from Greenland (G) or collected elsewhere, are divided according to the number of representatives for each class.

**Figure 5.**
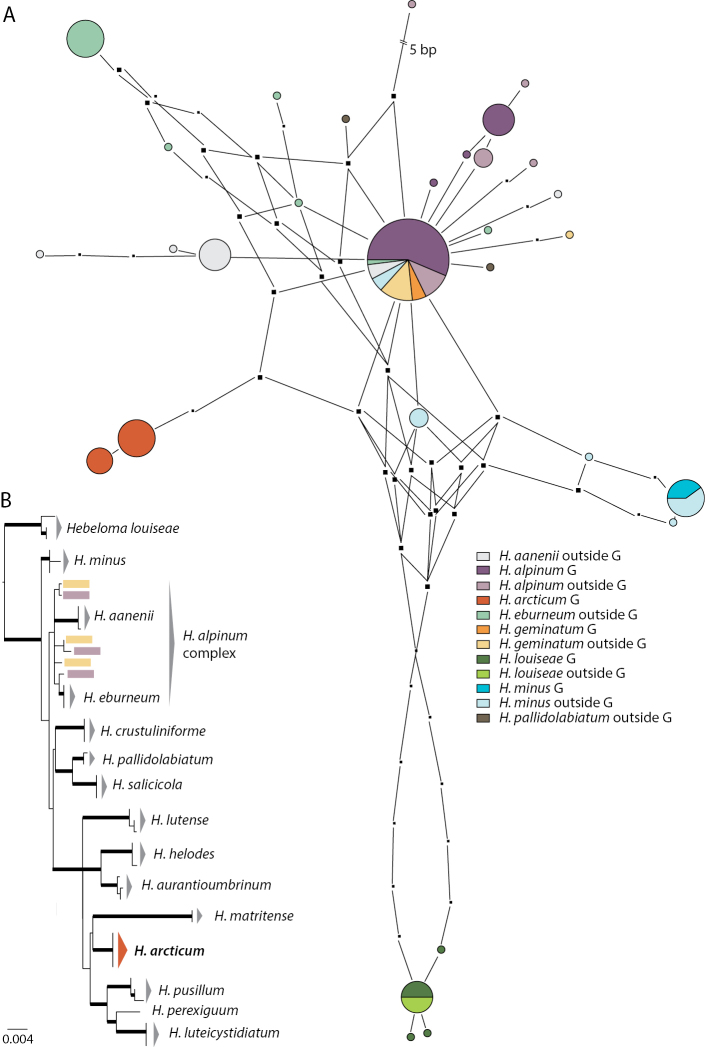
**A** Pruned quasi-median network of the *Hebeloma alpinum* complex, *H. arcticum* and *H. louiseae.* Circles shared by two or more taxa or collections from two regions, from Greenland (G) or collected elsewhere, are divided according to the number of representatives for each class **B** ML result for Hebeloma subsect. Crustuliniforme, based on concatenated ITS, *RPB2* and *Tef1*a data, rooted with *H. louiseae*. Thick branches indicate bootstrap support of at least 80% (450 bootstrap replicates). Species are represented by three collections (if available); *Hebeloma arcticum* by four collections including the holotype.

**Figure 6.**
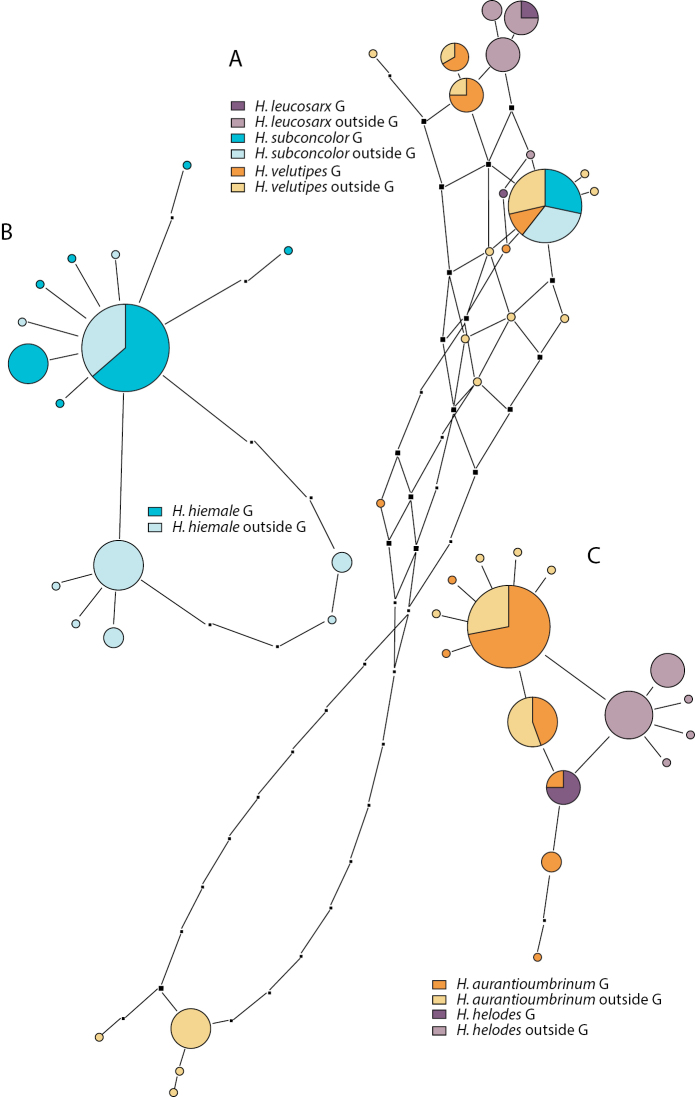
Pruned quasi-median networks of **A** the *Hebeloma velutipes* complex **B***H. hiemale* and **C***H. aurantioumbrinum* and *H. helodes*. Circles shared by two or more taxa or collections from two regions, from Greenland (G) or collected elsewhere, are divided according to the number of representatives for each class.

**Figure 7.**
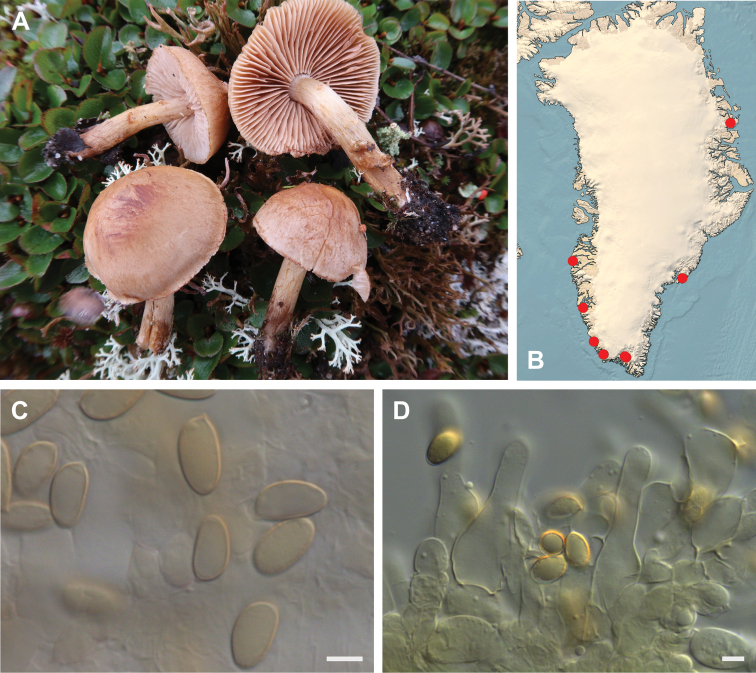
*Hebeloma alpinicola***A** HK18.322, photograph H. Knudsen **B** distribution of cited collections **C** spores ×1600 and **D** cheilocystidia ×1000 of HK18.322 in Melzer’s reagent. Scale bars: 5 µm; microphotographs H.K.J. Beker.

The data was divided into seven datasets for the network analyses and are depicted in Figs 3–7: Three networks for H. sect. Hebeloma, three networks for H. sect. Denudata and one for H. sect. Velutipes. The molecular result for the Greenland *H. islandicum* collection is treated in the Taxonomy part where the species is discussed.

The *H. mesophaeum* complex network (Fig. 3A) includes 124 sequences of which 76 are from Greenland material. The only species which has ‘private’ ITS variants, i.e. not mixing with other species’ sequences, is *H. colvinii*. Fourteen collections of *H. alpinicola* and six of *H. dunense* have ITS variants that occur in both species. The overlap concerns only Greenland collections of *H. alpinicola*, although Greenland collections do not generally appear to be segregated from non-Greenland collections. Some (5) *H. mesophaeum* ITS variants coincide with *H. excedens* (7) and others (11) with *H. pubescens* (6), irrespective of geographical origin.

No overlap was observed between *H. marginatulum* ITS variants and those of other species. The network (Fig. 3B) gives evidence of the ITS sequence diversity of the species. The network includes 92 sequences, of which 42 were from Greenland. The circles representing only non-Greenland collections in the upper part of the network only include North American sequence variants, but other North American collections are also represented by circles in the lower part of the diagram. The ITS of the Greenland collections do not appear to stand out in this circumpolar sample.

Fig. 4A shows the network of the *H. nigellum* complex and *H. grandisporum.* The analysis included 144 sequences of which 73 stemmed from Greenland material. Only a partial ITS sequence of the single collection of *H. clavulipes* from Greenland could be generated and was thus not included in the analysis. *Hebeloma grandisporum* is the only species in this network that is distinct; it is separated by at least 5 alignment positions from the sequences of the members of the *H. nigellum* complex. The ITS of *H. fuscatum* is split between one group of sequences that differs in at least 3 alignment positions from all other sequences in the analyses and a number (5) of Greenland collections that group together with sequences of *H. oreophilum* (20) and *H. clavulipes* (7). The latter two species are not separable by ITS. Similarly, *H. nigellum* cannot be separated from *H. spetsbergense*; 29 collections of *H. nigellum* and 12 of *H. spetsbergense* are represented in the shared circles. Some collections of *H. hygrophilum* (4) mix with *H. nigellum* and *H. spetsbergense* in the network.

Fig. 5A shows the ITS network of the *H. alpinum* complex in a broad sense, including *H. minus* and *H. pallidolabiatum*, as well as *H. louiseae* and *H. arcticum*. The latter species is described as new in this paper. The network includes 109 sequences, of which 54 are from Greenland material. Eight species are included in the network; five of these have been collected in Greenland and *H. arcticum* is represented exclusively by Greenland material. *Hebeloma louiseae* and *H. arcticum* are clearly distinct and do not share any ITS variants with other species. All member species of the *H. alpinum* complex, in this broad sense, either have some members that are represented in the central circle of the network or are linked through it (*H. pallidolabiatum*). All members of *H. geminatum* (12) and the majority of collections of *H. alpinum* (35), irrespective of origin, are represented in the central circle. Additional information on the results referring to *H. arcticum* are given in the context of the species description below.

In Fig. 5B the position of *H. arcticum* within H. subsect. Crustuliniformia is shown in the result of the ML analysis. The species clade of *Hebeloma arcticum* received full bootstrap support (100%) and formed a highly supported clade (99%) with the species clades of *H. aurantioumbrinum*, *H. helodes*, *H. luteicystidiatum*, *H. lutense*, *H. matritense*, *H. perexiguum* and *H. pusillum* all of which are also fully supported, apart from *H. perexiguum* which is only known from the type.

The networks relating to other members of H. sect. Denudata are depicted in Figs 4B, 6A and 6C. The network of members of H. subsect. Clepsydroida (Fig. 4B) includes 47 sequences (16 from Greenland) from three species. *Hebeloma ingratum* is distinct from the other two species in the network, separated by at least 5 alignment positions.

Sequences from Europe and Greenland are represented in the same circle. *Hebeloma cavipes* (4 sequences), not present in Greenland and *H. vaccinum* (13 sequences) share one ITS variant; there is no taxonomic or geographical structure apparent in the *H. cavipes*/*H. vaccinum* part of the network.

*Hebeloma hiemale* (Fig. 6A), not unlike *H. marginatulum*, is a species that is distinct from its relatives based on ITS, but with a high intraspecific variation in the ITS. Greenland collections (42 out of a total of 86 sequences in the analysis) are concentrated in the upper part of the network, but the circles that only include non-Greenland collections include European as well as North American collections.

The network of *H. aurantioumbrinum* and *H. helodes* (Fig. 6C) shows that in Greenland *H. helodes* cannot be distinguished based on ITS (the mixed circle represents three sequences of *H. helodes* and one of *H. aurantioumbrinum*, all from Greenland), although outside Greenland *H. helodes* appears to be better distinct from *H. aurantioumbrinum*. The network is based on 80 sequences, of which 43 are of Greenland origin.

Three species of H. sect. Velutipes have been collected in Greenland. The respective network is depicted in Fig. 6B and is based on 65 sequences of which 20 were from Greenland collections. *Hebeloma leucosarx* differs by at least one alignment position from the other two species; *H. subconcolor* and *H. velutipes* sequences are represented by the biggest circle (28 sequences) of the network. Neither in the Greenland sample nor in the US sample, did *H. velutipes* ITS variants occur that were more similar to *H. incarnatulum* (at the bottom of the network; see figure 5 of Cripps et al. (2019) for the placement of *H. incarnatulum*) than to *H. leucosarx*. The circle representing *H. subconcolor* and *H. velutipes* includes 17 sequences from the former and 11 from the latter.

## Taxonomic treatment

### Hebeloma

Taxon classificationFungiAgaricalesHymenogastraceae

(Fr.) P. Kumm.

C28B5C15-CF7E-5AEA-8C15-B25B938CD71A

#### General description.

Cap 0.9–21 cm, convex or more rarely campanulate or applanate, rarely depressed at center, dry, in wet weather viscid in some species, or tacky, smooth or rarely scaly, margin even or undulate, smooth, rarely hygrophanous, rarely striate, several shades of brown, from whitish to pale beige to dark brown, occasionally orange-brown, rarely reddish brown, along margin with or without remnants of universal veil, floccose or velutinate. Lamellae emarginate to adnate, thin, unicolored, at first pale, at maturity browner, in some groups with hyaline or brown droplets along margin. Stem 2.0–14.0 × 0.1–2.0 cm, cylindrical or bulbous at base, occasionally rooting, at first white, whitish or cream, becoming pale ochraceous to brown or even black, white tomentose, floccose or pruinose, rarely velutinate, some groups with partial veil, rarely with a membranous ring. Flesh color resembling the color of the stem, often discoloring brownish from stem base when bruised or old. Smell often radish-like, in one section sweetish, in some species insignificant. Taste mild to bitter, rarely significant. Spore deposit umber to dark cinnamon or dark brick red.

Spores most often amygdaloid, also ellipsoid or limoniform, rarely fusoid or navicular, in some species distinctly papillate, from pale yellow to dark brown, almost smooth to distinctly verrucose and ornamented with separate or coherent irregular warts, in some groups with ± loosening perispore, in Melzers solution from indextrinoid to distinctly and strongly dextrinoid. Cheilocystidia present in all species, variable but distinctive, cylindrical, lageniform, slenderly clavate, spheropedunculate, ventricose or balloon-shaped or variations thereof, rarely forked, in some species partially thick-walled, straight, sinuate or geniculate, hyaline or pale brown. Pleurocystidia rarely present, when present usually similar to cheilocystidia. Caulocystidia present in all species, similar to cheilocystidia, often fasciculate. Basidia cylindrical to slenderly clavate, 4-spored, rarely also 2-spored, in one arctic species only 2-spored. Pileipellis an ixocutis, a thin layer of narrow, hyaline hyphae, smooth or encrusted, 30–250 µm thick.

#### Comments.

*Hebeloma* is a genus of ectomycorrhizal fungi. The symbionts belong to a wide variety of families of dicotyledons. In Greenland, according to the observations of T.B., S.A.E. and H.K.K. symbionts appear to belong to the families: Salicaceae, Betulaceae, Polygonaceae and Rosaceae. The total list consists of only 11 species: *Salix glauca*, *S. herbacea*, *S. arctica* Pall., *S. arctophila*, Betula pubescens var. pumila, *B. nana.*, *B. glandulosa*, Alnus alnobetula subsp. crispa, *Bistorta vivipara*, *Dryas octopetala* and *D. integrifolia*.

*Bistorta vivipara* (L.) Delarbre (≡ *Polygonum viviparum* L., Polygonaceae) has often been observed close to species of *Hebeloma* and Brevik et al. (2010) isolated sequences of *Hebeloma* spp. from its roots in Spitsbergen, which is normally considered as a strong indication that this plant associates with the respective fungus. In a few cases, T.B. found rhizoids from *Hebeloma* attached to the roots of *Bistorta*, but found it difficult to rate the importance of these observations, since there have often been other possible hosts observed nearby. For *Alnus*, there is no evidence that it is a host; for the few records from *Alnus*-shrubs, it is not possible to rule out nearby willows or birches. The remaining perennial shrubs in Greenland, *Sorbus groenlandica* (C.K. Schneid.) Á. Löve & D. Löve and *Juniperus communis* L. do not form ectomycorrhiza. This is also the case for members of the heather family (Ericaceae) that occur in Greenland, with the rare exception of *Arctostaphylos alpina*.

### Key to sections and subsections of *Hebeloma* in Greenland, (including *H. islandicum*)

Spore character measures (O1–O4; P0–P3 and D0–D4) are explained in Beker et al. (2016: 22ff and figure 6A–G (color)).

**Table:** 

1	Veil present, either on the cap margin or from the margin to the stem or both; margin of lamellae rarely exuding small drops; most cheilocystidia distinctly ventricose	**H. sect. Hebeloma**
–	Veil absent (except in primordia); margin of lamellae when fresh usually exuding droplets; most cheilocystidia distinctly swollen at apex	**2**
2	Cheilocystidia at apex and base enlarged (hourglass-shaped), apically or in the middle sometimes with thickened walls	**3**
–	Cheilocystidia distinctly enlarged at apex, below ± cylindrical, walls rarely thickened	**5**
3	Spores O2 and not O3 and many spores D3, found in alpine-arctic habitats	***H. islandicum***
–	Above conditions not satisfied	**4**
4	Many spores O1 or O2 and many spores D0 or D1 and spores not P2 or if spores are P2 then pileus is not yellow or cream in the center but with brown or buff tones	***H. hiemale* (H. subsect. Hiemalia)**
–	The above conditions not satisfied	**H. subsect. Clepsydroida**
5	Cheilocystidia distinctly restricted below apex, hardly swollen in lower third	**H. subsect. Crustuliniformia**
–	Cheilocystidia more gently tapering downwards, some ventricose cystidia present	**H. sect. Velutipes**

### Key to species of Hebeloma section Hebeloma in Greenland

**Table:** 

1	Spores mainly ellipsoid, indextrinoid to weakly dextrinoid	**2**
–	Spores mainly amygdaloid, usually rather strongly to strongly dextrinoid	**8**
2	Number of full-length lamellae (L) < 32 and pileus with a matting of short soft hairs	***H. pubescens***
–	Number of full-length lamellae ≥ 32 or if < 32 then pileus with at most a few fibrils at margin	**3**
3	Spores on ave. at least 12.5 × 7.5 µm	***H. colvinii***
–	Spores smaller	**4**
4	Cap distinctly bicolored, at least in mature specimens	***H. mesophaeum***
–	Cap mainly unicolored, at most indistinctly bicolored	**5**
5	Spores clearly ellipsoid to ovoid, very rarely amygdaloid, ave. size rarely exceeding 10 × 6 µm	**6**
–	Ave. spore length > 10 μm and ave. spore width ≥ 6 μm or if spores smaller, then most spores ellipsoid, but many amygdaloid, almost always with Salicaceae	**7**
6	Stem relatively narrow, usually 0.2–0.6 cm thick, cap often overhanging lamellae	***H. excedens***
–	Stem relatively robust, usually 0.5–1 cm thick, cap not overhanging lamellae	***H. alpinicola***
7	Spores with some clear ornamentation (O1,O2) and an indistinct but clear reaction in Melzer’s reagent (D1)	***H. dunense***
–	Spores showing almost no ornamentation, even under immersion (O1) or completely indextrinoid (D0)	***H. marginatulum***
8	Number of full-length lamellae (L) ≥ 40	**9**
–	Number of full-length lamellae (L) < 40	**10**
9	Spores, on ave. ≥ 6.7 µm wide	***H. oreophilum***
–	Spores, on ave. < 6.7 µm wide	***H. clavulipes***
10	Ave. spore length ≥ 15 μm	***H. grandisporum***
–	Ave. spore length < 15 μm	**11**
11	Spore papilla distinctly present and many spores limoniform	***H. fuscatum***
–	Spore papilla at most indistinctly present and spores rarely limoniform	**12**
12	Ave. spore width > 7.5 μm	***H. spetsbergense***
–	Spores amygdaloid	**13**
13	Spores < 7 µm wide	***H. hygrophilum***
–	Spores ≥ 7 µm wide	***H. nigellum***


### Key to species of Hebeloma section Denudata in Greenland

**Table:** 

1	Cheilocystidia at apex and base enlarged (hourglass-shaped), apically or in the middle sometimes with thickened wall	**2**
–	Cheilocystidia distinctly enlarged at apex, below ± cylindrical, walls rarely thickened	**4**
2	Spores on ave. 12–14.5 µm long, rather strongly dextrinoid (D3); cap at center rust brown to reddish brown	***H. vaccinum***
–	Spores on ave. 10–12.5 µm long, less dextrinoid (D2); cap at center paler	**3**
3	Spores on ave. 10–11 µm long, O2 to O3, D1 to D2	***H. ingratum***
–	Spores on ave. 10–12.5 µm long, O1 to O2, Do to D1	***H. hiemale***
4	Number of full-length lamellae (L) ≥ 60 and ave. spore length < 11 μm	***H. geminatum***
–	Number of full-length lamellae (L) < 60 or ave. spore length ≥ 11 μm	**5**
5	Spores O2 to O3 and D2 to D3, many D3	***H. arcticum***
–	Spores at most D2	**6**
6	Number of full-length lamellae (L) ≥ 40, spore length ≥ 11 μm, spores with distinct papilla	***H. alpinum***
–	Any of the above conditions not satisfied	**7**
7	Ave. spore length < 11 μm	**8**
–	Ave. spore length ≥ 11 μm	**9**
8	Cheilocystidia without consistent and distinct apical thickening	***H. aurantioumbrinum***
–	Cheilocystidia with consistent and distinct apical thickening	***H. helodes***
9	Spores O1 or O2, few if any spores O3	***H. louiseae***
–	A large number of spores O3	***H. minus***

### Key to species of Hebeloma section Velutipes in Greenland

**Table:** 

1	No. of full-length lamellae < 35	***H. subconcolor***
–	No. of full-length lamellae > 35; stem base ± bulbose	**2**
2	Cap distinctly colored, often quite dark with reddish tones, umbonate	***H. leucosarx***
–	Cap pale colored, whitish to beige or buff, convex, sometimes umbonate	***H. velutipes***

### Hebeloma sect. Hebeloma

Veil present, often along cap margin. Cap uniformly colored or bicolored, with a paler margin. Lamellae without droplets. Stem fibrillose, often browning from base. Spores ellipsoid, ovoid, amygdaloid or limoniform, ornamentation weak, dextrinoid or non-dextrinoid, perispore not or only very slightly loosening.

### Hebeloma alpinicola

Taxon classificationFungiAgaricalesHymenogastraceae

A.H. Sm., V. S. Evenson & Mitchel; Veiled species of Hebeloma in the western United States (Ann Arbor): 48, 1983.

DE4B1B20-4F86-5248-8C73-85C839D4C477

Fig. 7

#### Macroscopic description.

Cap 1.2–4.5 cm in diameter, robust, fleshy, irregularly hemispherical to convex, somewhat domed or not, almost unicolored, center cinnamon to dark pinkish buff, dark olive buff or rarely umber, sometimes with grayish tones, outwards ocher and lighter buff not white towards margin, sometimes with hoary canescent coating that dries shiny, with weakly hygrophanous spots, dry, margin inrolled at first, and then turned down, partial veil present. Lamellae narrowly attached, slightly emarginate, or with a tooth, or pulling away, somewhat broad (2‒5 mm), pale gray brown to milky coffee, number of lamellae {L} 30–44, edges white floccose, without watery droplets. Stem 1.5–7.0 × 0.2–0.8 cm, cylindrical, whitish pruinose at apex, often with a whitish ring zone, dingy ocher to sordid yellowish or fairly dark brown, longitudinally strongly fibrillose and striate in lower part, often darkening when bruised, base sometimes encrusted with sand or earth, solid or slightly hollow. Context dingy whitish, darker below, unchanging or flesh staining brown. Smell weakly raphanoid. Taste almost insipid to slightly bitter. Spore deposit near fulvous.

#### Microscopic description.

Spores ellipsoid or some slightly amygdaliform or ovoid, with rounded end, apiculus small, on ave. 8–10.5 × 5–6 µm, ave. Q = 1.55–1.85, smooth to slightly rough (O1), not guttulate, not or very slightly dextrinoid (D0D1), perispore not loosening (P0). Basidia clavate, mostly four-spored, 20–35 × 6–8 µm. Cheilocystidia lageniform or ventricose, on ave. 35–55 × 5–6.5 (apex) × 4.5–6.5 (middle) × 6.5–10 (base) µm, occasionally geniculate or bifurcate, sometimes septate. Ratios A/M = 0.95‒1.24, A/B = 0.54‒0.88, B/M = 1.43‒2.17. Epicutis an ixocutis, up to 200 µm thick, maximum hyphae width to 8 µm, encrusted hyphae not seen, shape of trama elements beneath subcutis cylindrical. Caulocystidia similar to cheilocystidia, but generally larger.

#### Collections examined.

**S-Greenland**: Kangilinnguit, 61.23°N, 48.07°W, 12 Aug 1985, T. Borgen (TB85.099, C-F-5082), 95 m, with *Salix glauca* and *Chamaenerion latifolium* in heathland. Kangilinnguit, 61.23°N, 48.10°W, 6 Aug 1984, T. Borgen (TB84.063, C-F-103534), 25 m, with *Salix glauca* in scrubland. Kangilinnguit, 61.23°N, 48.07°W, 17 Aug 1985, T. Borgen (TB85.071, C-F-5081), 95 m, with *Alnus alnobetulae*. Narsarsuaq, 61.15°N, 45.42°W, 9 Aug 2018, H. Knudsen (HK18.010, C-F-111109), 185 m, with *Salix glauca* and *Betula pubescens* in scrubland. Narsarsuaq, airport area, 61.08°N, 45.26°W, 3 Aug 1984, T. Borgen (TB84.028, C-F-103559), 20 m, with *Salix glauca* and *Betula glandulosa* in scrubland. Paamiut, 62.01°N, 49.4°W, 1 Aug 2000, T. Borgen (TB00.049, C-F-103516), 10 m, with *Salix herbacea* in snowbed. Paamiut, 62.01°N, 49.4°W, 8 Aug 1981, T. Borgen TB81.111, C-F-103554), 25 m. Paamiut, 62.01°N, 49.4°W, 20 Aug 1985, T. Borgen (TB85.218, C-F-103532), 30 m, with *Salix herbacea* and *Bistorta vivipara* in snowbed. **W-Greenland**: Kobbefjord, NuukBasic Station, 64.078°N, 51.23°W, 25 Aug 2018, H. Knudsen (HK18.322, C-F-111116), 5 m, with *Salix herbacea* in tundra. Sisimiut, Kællingehætten, 66.93°N, 53.59°W, 16 Aug 2016, H. Knudsen (HK16.165, C-F-108401), 400 m. **N-Greenland**: Zackenberg, Ulvehøj, 74.47°N, 21°W, 7 Aug 1999, T. Borgen (TB99.238, C-F-119805), 40 m, with *Salix arctica* and *Bistorta vivipara*. **E-Greenland**: Kuummiut, Torsukattak, 65.87°N, 37.01°W, 2 Aug 2008, T. Borgen (TB08.039, C-F-101623), 35 m; with *Salix glauca* in heathland. Kuummiut, Torsukattak, 65.87°N, 37.01°W, 2 Aug 2008, T. Borgen (TB08.037, C-F-101621), 35 m.

#### Distribution.

Widely distributed in Greenland, only missing in Constable Pynt. Originally described from the Seven Devils Mountains in Idaho, North America by Smith et al. (1983), more recently from the Rocky Mountains by Cripps et al. (2019) and from the European Alps by Grilli et al. (2020). This species appears to favor both boreal and arctic or alpine habitats. Most collections from Rocky Mountains were from subalpine areas. In Greenland, it occurs from the Subarctic zone to the High Arctic zone, and the Greenland collections from Zackenberg are the northernmost known (74.47°N). Circumpolar, arctic-alpine.

#### Habitat and ecology.

Thirteen collections in all, mainly with *Salix glauca* in scrubs and heathland, three collections in snowbeds with *S. herbacea*, one collection with *S. arctica*, and one collection with Alnus alnobetulae ssp. crispa, but the presence of other possible associates should not be ruled out. In the Rocky Mountains, Cripps et al. (2019) found two collections, one with *Dryas*, *Salix planifolia* Pursh. and *S. reticulata* L., the other subalpine record with dwarf willows only. *Hebeloma alpinicola* seems not to be specific regarding soil conditions.

### Hebeloma clavulipes

Taxon classificationFungiAgaricalesHymenogastraceae

Romagn.; Bull. trimest. Soc. mycol. Fr. 81(3): 326, 1965.

ED38D480-E301-55D7-81D7-0D0F47BC270E

Fig. 8

#### Macroscopic description.

Cap 1.0‒4.1 cm in diameter, convex, sometimes umbonate, margin involute, rarely sulcate, tacky when moist, sometimes hygrophanous, uniformly colored or variably bicolored, at center umber to sepia (6E6, 6E7, 6F6, 6F7), margin cream to pinkish-buff to ochraceous or dark olive buff (4A2, 4A3, 5A3, 5B3, 5B5, 5C5); remains of universal veil sometimes present; partial veil present. Lamellae adnate or adnexed to emarginate, pale brown, maximum depth 3‒5 mm, number of lamellae {L} 40‒48, droplets usually absent, but occasionally visible with × 10 lens, white fimbriate edge usually present, but sometimes indistinctly. Stem 1.3‒7.0 × 0.3‒0.6 cm, 4.5‒7.3(–8) cm at base, stem Q 3.3‒15.5(–18), at first whitish fibrillose becoming pale brown, base cylindrical to clavate or bulbous, usually pruinose or floccose at apex. Context firm, stem inside stuffed, later hollow, stem flesh discoloring from base, sometimes weakly. Smell raphanoid, sometimes with hints of cocoa. Taste mild to bitter, usually raphanoid. Spore deposit brownish olive (5E5).

**Figure 8.**
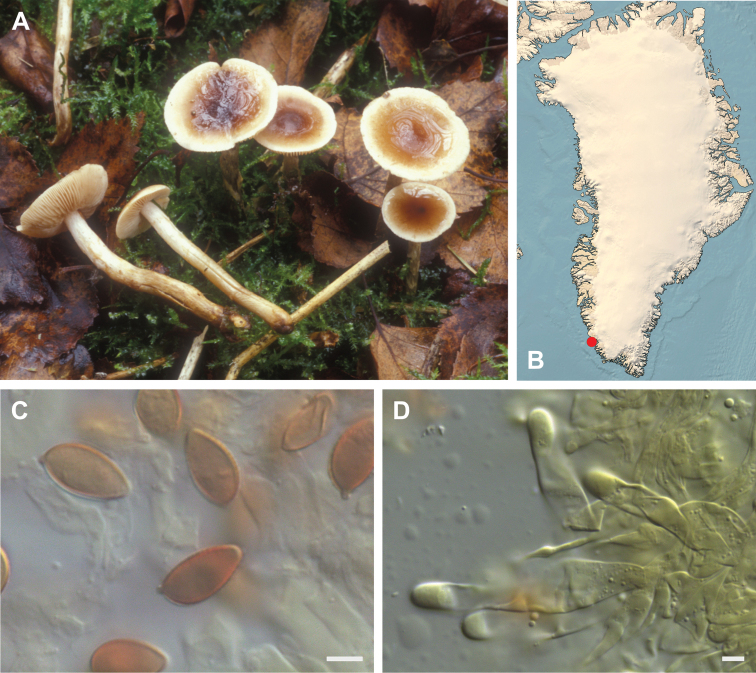
*Hebeloma clavulipes***A** JV02-517 (from Denmark), photo J. Vesterholt, reproduced by kind permission from Beker et al. (2016)**B** distribution of cited collections **C** spores ×1600 and **D** cheilocystidia ×1000 of TB90.100 in Melzer’s reagent. Scale bars: 5 µm; microphotographs H.K.J. Beker.

#### Microscopic description.

Spores amygdaloid or limoniform, occasionally appearing ovoid or fusoid, on ave. 10.5‒12.5 × 6.0‒6.5 µm, ave. Q 1.6‒2.1, very weakly ornamented (O1O2), perispore not or somewhat loosening (P0P1), rather strongly dextrinoid (D2D3), yellow to yellow brown, ± guttulate, papillate. Basidia 23‒32(‒36) × 7‒10 µm, ave. Q = 2.6‒4.4, mostly four-spored. Cheilocystidia lageniform to ventricose, occasionally cylindrical, occasionally with a characteristic thickening, apically, basically or medially, sometimes geniculate or septate, on ave. 41‒60 × 4.5‒6 (apex) × 4‒5.5 (middle) × 7.5‒12 (base) µm, ratios A/M = 0.93‒1.25, A/B = 0.45‒0.76, B/M = 1.58‒2.31. Epicutis an ixocutis, 125–170 µm thick (measured from exsiccata), maximum hyphae width 3.5‒7 µm, sometimes encrusted, shape of trama elements beneath subcutis sausage-shaped, up to 14 µm wide. Caulocystidia similar to cheilocystidia, but usually less ventricose, up to 120 µm long.

#### Collections examined.

**S-Greenland**: Paamiut, W of the Navigation School area, 62°N, 49.67°W, 7 Sep 1990, T. Borgen (TB90.100, C-F-119760), 20 m, with *Salix herbacea*.

#### Distribution.

Only one record and apparently a very rare species in Greenland recorded only once during the 20 years when Borgen regularly collected in Paamiut. The general distribution in Europe (Beker et al. 2016) is from the Temperate zone to southern Boreal and Subalpine zone up to 1700 m. The Greenland record is the northernmost known (62.00°N). Temperate, boreal, subarctic, and now also from Low Arctic zone.

#### Habitat and ecology.

Only one collection, with *Salix herbacea*. In Europe, the hosts are *Betula*, *Picea* and *Salix* (Beker et al. 2016), but the associated *Salix* species are of the shrub-like kind, like *S. aurita*.

### Hebeloma colvinii

Taxon classificationFungiAgaricalesHymenogastraceae

(Peck) Sacc.; Syll. fung. (Abellini) 5: 805, 1887.

4ADC6302-33D0-5F2E-9BB5-68DD7ECEC548

Fig. 9

#### Macroscopic description.

Cap 2.5–7.5 cm, convex to irregularly gibbous, sometimes broadly umbonate, with incurved later straight, opaque margin, occasionally appendiculate, dry and dull, unicolored mainly sepia, sometimes with light grayish adpressed covering. Lamellae almost free to emarginate gray (olive) brown, edge entire, slightly tomentose, number of lamellae {L} 45–58. Stem whitish pale fibrillose, sordid gray-brown, often more or less buried in sand with a sand bulb around the base, cortina pale. Context initially fairly thick, with cavity, firm watery grayish in pileus, pale brownish in stipe to near buff, towards base dark, sordid brown. Smell weakly and indistinctly raphanoid. Taste similar, mild. Spore deposit color not recorded.

**Figure 9.**
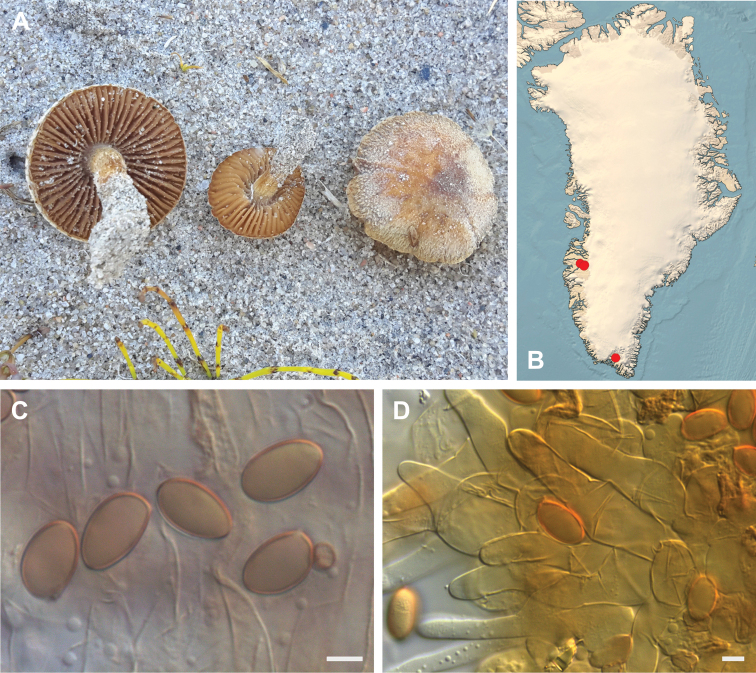
*Hebeloma colvinii***A** HK16.005, photograph H. Knudsen **B** distribution of cited collections; **C** spores ×1600 and **D** cheilocystidia ×1000 of HK16.005 in Melzer’s reagent. Scale bars: 5 µm; microphotographs H.K.J. Beker.

#### Microscopic description.

Spores ellipsoid, often ovoid or amygdaloid on ave. 12.0‒14.5 × 7.5‒8.5 µm, ave. Q 1.5‒1.9, very weakly ornamented (O1O2), perispore not noticeably loosening (P0), indistinctly dextrinoid (D0D1), yellow brown, ± guttulate, not papillate. Basidia 26‒42 × 7‒11 µm, ave. Q = 3.0‒4.2, mostly four-spored. Cheilocystidia lageniform to ventricose, occasionally cylindrical or clavate-ventricose, occasionally with a characteristic wall thickening, apically, basically, sometimes septate, on ave. 40‒50 × 5.5‒8 (apex) × 5‒9 (middle) × 10‒13 (base) µm, ratios A/M = 0.90‒1.26, A/B = 0.46‒0.77, B/M = 1.40‒2.45. Epicutis an ixocutis, up to 120 µm thick (measured from exsiccata), maximum hyphae width 10 µm, sometimes encrusted, shape of trama elements beneath subcutis sausage-shaped, cylindrical or ellipsoid. Caulocystidia similar to cheilocystidia, but usually less ventricose, up to 130 µm long.

#### Collections examined.

**S-Greenland**: Narsarsuaq, 61.16°N, 45.43°W, 31 Aug 2002, T. Borgen (TB02.166, C-F-106756), 30 m, with *Salix glauca* in riverbed. **W-Greenland**: Kangerlussuaq, Sandflugtsdalen, 67.13°N, 51.16°W, 7 Aug 2016, T. Borgen (TB16.075, C-F-103585), 50 m, with *Salix glauca* at riverside dune. Kangerlussuaq, Sandflugtsdalen, 67.06°N, 50.46°W, 7 Aug 2016, H. Knudsen (HK16.008, C-F-104038), 200 m, with *Salix glauca*. Kangerlussuaq, Sandflugtsdalen, 67.06°N, 50.46°W, 7 Aug 2016, H. Knudsen (HK16.005, C-F-104035), 200 m, with *Salix glauca*. Kangerlussuaq, southeast of Sugar Loaf, 66.989438°N, 50.548760°W, 25 Aug 2015, S.A. Elborne (SAE-2016.188GR, C-F-107346), 50 m with *Salix glauca* at riverside dune.

#### Distribution.

Known from a few collections from three localities in southwestern Greenland. *H. colvinii* was described by Peck (1875; effectively published 1876) from West Albany in North America. He found it in drifting sand in West Albany near Albany, N.Y. State. Later Kauffman (1918) reported this or a very similar species from New Richmond in Michigan, as did Ritchie (1946) from Lewes, Delaware Bay. Neither of these two collections has been examined as part of this study.

#### Habitat and ecology.

Five collections, all with *Salix glauca* in pure sand along a river. In one of the localities, Sandflugtsdalen (“valley of driftsand”) at Kangerlussuaq, it was very common and scattered over a large area. The sand is mineral-rich due to mixing with loess.

#### Note.

*Hebeloma colvinii* was originally described as “Agaricuscolvini” (Peck 1875). The name is currently not in use, e.g. there are no records on Mushroom Observer (https://mushroomobserver.org/, accessed 23 Sept 2020) and while Mycoportal (https://mycoportal.org/portal/collections/list.php, accessed 2 Dec 2020) has 63 records, the most recent is from 1972. The identification of the Greenland collections is supported by unpublished studies of type material. It appears that exposed expanses of sandy soil are the characteristic habitat for the species, which is morphologically similar to a number of species of the difficult group around *H. dunense* and *H. mesophaeum*. *Hebeloma colvinii* can be recognized by its large ellipsoid spores, reminiscent of *Hebeloma psammophilum*, which is currently known only from western Europe (Beker et al. 2016).

### Hebeloma dunense

Taxon classificationFungiAgaricalesHymenogastraceae

L. Corb. & R. Heim; Mém. Soc. natn. Sci. nat. Cherbourg 40 (2): 166, 1929.

79C783AB-418D-5DC3-8EA9-A61F445F1660

Fig. 10

#### Macroscopic description.

Cap 1.0–7.0 cm in diameter, convex to umbonate, sometimes papillate, sometimes umbilicate, margin often involute when young, sometimes scalloped or serrate, becoming wavy or upturned with age, sometimes fibrillose along margin, tacky when moist, sometimes but not consistently hygrophanous, uniformly colored or variably bicolored, at center clay buff, dark olive buff, ochraceous or yellowish brown to brownish olive or umber or sepia to dark brick or orange brown or fuscous, at margin cream to buff or honey to pinkish buff to dark olive buff or yellowish brown to umber or sepia, sometimes with remains of universal veil, partial veil present. Lamellae light gray brown or very pale clay, later brownish clay, adnexed to adnate, usually emarginate, sometimes with decurrent tooth and occasionally decurrent, maximum depth 2.5–9 mm, number of lamellae {L} 20–48, droplets usually absent, but occasionally visible with ×10 lens, white fimbriate edge usually present, but often weak. Stem (1.0–)1.4–8.0 × (0.2–)0.3–1.2 cm, at base 0.2–1.0(–1.2) cm, stem Q = 3.5–15, base cylindrical, sometimes slenderly clavate, occasionally with sand bulb, fibrillose, pruinose or floccose at apex. Context firm, stem interior stuffed, later hollow, occasionally with superior wick, flesh usually discoloring from base, sometimes very strongly. Smell raphanoid, rarely without smell, occasionally hints of cocoa; taste mild to raphanoid to bitter. Spore deposit dark olive buff to brownish olive to umber to fuscous or sepia.

**Figure 10.**
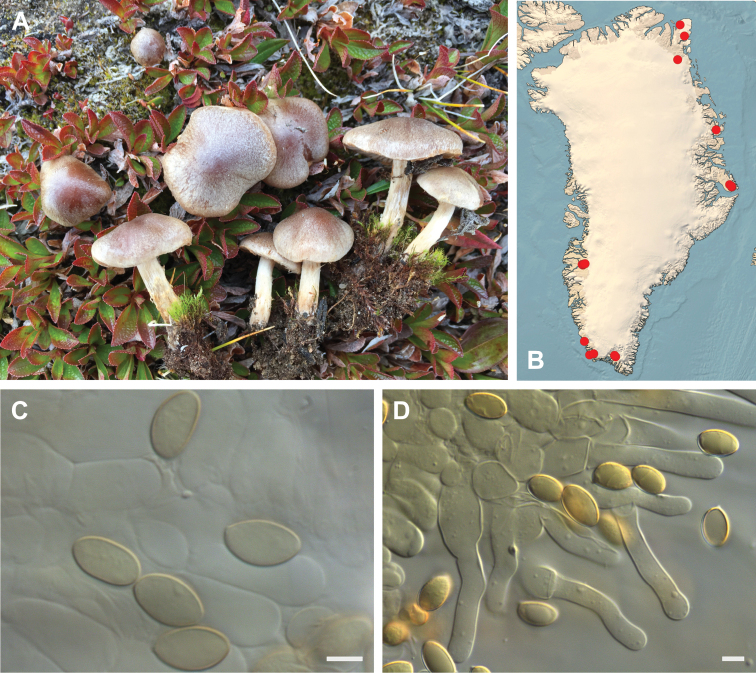
*Hebeloma dunense***A** SAE-2017.103, photograph S.A. Elborne **B** distribution of cited collections **C** spores ×1600 and **D** cheilocystidia ×1000 of SAE-2017.103 in Melzer’s reagent. Scale bars: 5 µm; microphotographs H.K.J. Beker.

#### Microscopic description.

Spores mainly ellipsoid, some amygdaloid or ovoid, not papillate, 10.0–12.5 × 6.0–7.5 µm, ave. Q 1.5–1.9, very weakly ornamented (O1O2), perispore not or somewhat loosening (P0P1), indistinctly dextrinoid (D0D1), yellow through yellow brown to brown, ± guttulate. Basidia 20–33 × 7–9 µm, ave. Q = 2.8–4, mostly four-spored. Cheilocystidia usually lageniform or ventricose, sometimes cylindrical, occasionally with characteristic wall thickenings, apically, medially or basically, occasionally bifurcate, geniculate, septate (sometimes clamped), subcapitate, 34–57 × 4.5–8 (apex) × 4–7 (middle) × 7–12 (base) µm, with yellow contents, ratios A/M = 0.82–1.43, A/B = 0.42–0.89, B/M = 1.4–2.14. Epicutis an ixocutis, 25–75 µm thick (measured from exsiccata), ixocutis maximum hyphae width 4–8 µm, hyphae occasionally encrusted, shape of trama elements beneath subcutis angular, ellipsoid, isodiametric, spherical or sausage-shaped up to 20 µm wide. Caulocystidia similar to cheilocystidia, but usually more irregular and often multi-septate.

#### Collections examined.

**S-Greenland**: Kangilinnguit, 61.14°N, 48.6°W, 15 Aug 1985, T. Borgen (TB85.200, C-F-103530), 400 m, with *Salix herbacea* and *Dryas integrifolia* in snowbed. Kangilinnguit, 61.23°N, 48.10°W, 8 Aug 1984, T. Borgen (TB84.090, C-F-103535), 25 m, with *Salix herbacea* along streamside. Kangilinnguit, at Grønnedal Hut, 61.23°N, 48.08°W, 15 Aug 1985, T. Borgen (TB85.183, C-F-103589), 180 m. Kangilinnguit, near Grønnedal Hut, 61.23°N, 48.08°W, 15 Aug 1985, T. Borgen (TB85.186, C-F-103527), 350 m, with *Salix arctophila* in streamside. Narsarsuaq, 61.08°N, 45.26°W, 1 Aug 1991, T. Borgen (TB91.045, C-F-103486), 100 m, with *Salix glauca*. Narsarsuaq, 61.17°N, 45.41°W, 17 Aug 2015, H. Knudsen (HK15.078, C-F-8231), 60 m, with *Dryas* sp. along pathside. Paamiut, 62.01°N, 49.4°W, 12 Aug 1984, T. Borgen (TB84.114, C-F-103536), 10 m, with *Salix glauca* and *Salix herbacea*. **W-Greenland**: Kangerlussuaq, airport area, 67.02°N, 50.72°W, 10 Aug 1986, T. Borgen (TB86.177, C-F-103563), 30 m, with *Salix glauca*. Kangerlussuaq, near the inland ice, 67.09°N, 50.25°W, 12 Aug 2000, K. Kalamees (HK00.032, C-F-7881), 200 m, in tundra. Kangerlussuaq, Sandflugtsdalen, 67.06°N, 50.46°W, 7 Aug 2016, H. Knudsen (HK16.015, C-F-104045), 200 m, with *Salix glauca*. Kangerlussuaq, Sandflugtsdalen, c. 15 km E of Base, 67.04°N, 50.53°W, 7 Aug 2016, T. Borgen (TB16.076, C-F-104293), 50 m, with *Salix glauca*. Kangerlussuaq, Store Saltsø, 66.98°N, 50.6°W, 8 Aug 1986, T. Borgen (TB86.159, C-F-5087), 200 m, with *Salix glauca*. **N-Greenland**: Amdrup Land, 80.81°N, 17.32°W, 19 Jul 1993, B. Fredskild (s.n., C-F-7017), 225 m, with *Salix arctica* in tundra. Blåsø, Kronprins Christians Land, 79.62°N, 23.33°W, 4 Aug 1987, C. Bay (s.n., C-F-6994), 100 m. Prinsesse Dagmars Halvø, Knuths Fjeld, 81.58°N, 16.77°W, 6 Aug 1986, C. Bay (s.n., C-F-4216), 15 m. Zackenberg, at the S bank of Kærelv, 74.5°N, 21°W, 27 Jul 1999, T. Borgen (TB99.114, C-F-119746), 50 m, with *Dryas* sp. and *Salix arctica* in scrubland. Zackenberg, in the new delta, 74.5°N, 21°W, 22 Aug 1999, T. Borgen (TB99.411, C-F-119748), 20 m, with *Salix arctica* in scrubland. Zackenberg, just N of Gadekæret, 74.5°N, 21°W, 5 Aug 1999, T. Borgen (TB99.219, C-F-119752), 20 m, with *Dryas* sp. and *Salix arctica* in scrubland. Zackenberg, S of E part of airstrip, 74.5°N, 21°W, 12 Aug 2006, T. Borgen (TB06.159, C-F-119774), 30 m, with *Salix arctica* in snowbed. Zackenberg, Zackenberg River, 74.5°N, 21°W, 23 Aug 2006, T. Borgen (TB06.263, C-F-119776), 20 m, with *Salix arctica* in riverbed. **E-Greenland**: Jameson Land, Constable Pynt, camp N of Katedralen, S of Ugleelv, 70.9°N, 22.92°W, 25 Jul 1989, J.H. Petersen (JHP 89.259, C-F-2561), 170 m. Jameson Land, Nerlerit Inaat/Constable Pynt, delta of Gåseelv valley, 70.76°N, 22.65°W, 12 Aug 2017, T. Borgen (TB17C.118, C-F-106782), 40 m, with *Bistorta vivipara* and *Salix arctica*. Jameson Land, Nerlerit Inaat/Constable Pynt, delta of Gåseelv valley, 70.76 22.65°W, 4 Aug 2017, T. Borgen (TB17C.037, C-F-106773), 40 m, with *Salix* sp. Jameson Land, Nerlerit Inaat/Constable Pynt, delta of Gåseelv valley, 70.76°N, 22.65°W, 12 Aug 2017, H. Knudsen (HK17.265B, C-F-105171), 40 m. Jameson Land, Nerlerit Inaat/Constable Pynt, delta of Gåseelv valley, 70.76°N, 22.65°W, 7 Aug 2017, H. Knudsen (HK17.147, C-F-105049), 40 m. Jameson Land, Nerlerit Inaat/Constable Pynt, delta of Gåseelv valley, 70.76°N, 22.65°W, 12 Aug 2017, H. Knudsen (HK17.265A, C-F-105170), 40 m. Jameson Land, Nerlerit Inaat/Constable Pynt, delta of Gåseelv valley, 70.76°N, 22.65°W, 6 Aug 2017, H. Knudsen (HK17.127, C-F-105028), 40 m. Jameson Land, Nerlerit Inaat/Constable Pynt, delta of Gåseelv valley, 70.77°N, 22.67°W, 5 Aug 2017, S.A. Elborne (SAE-2017.103-GR, C-F-106761), 40 m, with *Arctostaphylos alpina*. Jameson Land, Nerlerit Inaat/Constable Pynt, delta of Gåseelv valley, 70.77°N, 22.73°W, 11 Aug 2017, S.A. Elborne (SAE-2017.219-GR, C-F-106769), 40 m, with *Salix arctica* at riverside. Jameson Land, Nerlerit Inaat/Constable Pynt, delta of Gåseelv, Harris fjeld, 70.75°N, 22.68°W, 31 Jul 2017, T. Borgen (TB17C.006, C-F-106770), 100 m, with *Dryas* sp. in heathland. Jameson Land, Nerlerit Inaat/Constable Pynt, delta of Gåseelv, Harris fjeld, 70.75°N, 22.68°W, 3 Aug 2017, H. Knudsen (HK17.070, C-F-1049599, 100 m. Jameson Land, Nerlerit Inaat/Constable Pynt, Gåseelv valley, north side, 70.76°N, 22.69°W, 4 Aug 2017, H. Knudsen (HK17.088, C-F-104984), 160 m. Jameson Land, Nerlerit Inaat/Constable Pynt, Hareelv, 70.7°N, 22.68°W, 10 Aug 2017, T. Borgen (TB17C.094, C-F-106780), 200 m, with *Salix arctica* in snowbed. Jameson Land, Nerlerit Inaat/Constable Pynt, Hareelv, 70.7°N, 22.68°W, 2 Aug 2017, T. Borgen (TB17C.030, C-F-106772), 200 m, with *Salix arctica*. Jameson Land, Nerlerit Inaat/Constable Pynt, Hareelv, 70.7°N, 22.68°W, 2 Aug 2017, H. Knudsen (HK17.043, C-F-104932), 200 m. Jameson Land, Nerlerit Inaat/Constable Pynt, Hareelv, 70.7°N, 22.68°W, 2 Aug 2017, H. Knudsen (HK17.045, C-F-104934), 200 m. Jameson Land, Nerlerit Inaat/Constable Pynt, Hareelv, 70.7°N, 22.68°W, 2 Aug 2017, H. Knudsen (HK17.052, C-F-104941), 200 m. Jameson Land, Nerlerit Inaat/Constable Pynt, Hareelv, 70.7°N, 22.68°W, 2 Aug 2017, H. Knudsen (HK17.056, C-F-104945), 200 m. Jameson Land, Nerlerit Inaat/Constable Pynt, Hareelv, 70.71°N, 22.68°W, 10 Aug 2017, S.A. Elborne (SAE-2017.186-GR, C-F-106765), 200 m, with *Salix arctica* along streamside. Jameson Land, Nerlerit Inaat/Constable Pynt, middle of Hareelv valley, N side, 70.71°N, 22.73°W, 10 Aug 2017, H. Knudsen (HK17.203, C-F-105108), 320 m. Jameson Land, Nerlerit Inaat/Constable Pynt, Primulaelv, 70.74°N, 22.67°W, 1 Aug 2017, T. Borgen (TB17C.010, C-F-106771), 180 m, with *Salix arctica* and *Bistorta vivipara*. Jameson Land, Nerlerit Inaat/Constable Pynt, Primulaelv, 70.74°N, 22.67°W, 13 Aug 2017, H. Knudsen (HK17.282, C-F-105189), 180 m.

#### Distribution.

*Hebeloma dunense* is a common and widespread *Hebeloma* distributed throughout Mediterranean, Temperate, Boreal, Arctic and Alpine areas. It has been recorded in arctic and alpine areas of North America, Europe and Asia (Russia) (Beker et al. 2016). It has one of the most northern records for a *Hebeloma* at 81.58° in Greenland (Beker et al. 2016). It appears to be one of the most common and widespread of all *Hebeloma* species in arctic or alpine regions. Circumpolar, Temperate, Boreal, Subarctic and Arctic-Alpine.

#### Habitat and ecology.

Forty-two collections and one of the five most often collected species in Greenland with 11% of the collections. Most are with *Salix arctica* (10), *S. glauca* (6), *S. herbacea* (2), *S. arctophila* (1) or unspecified *Salix* in more or less calcareous habitats. The collection from Paamiut was from a man-made area, in this otherwise acid soil locality. Four collections were recorded with *Dryas integrifolia* and *D. octopetala* and one with *Arctostaphylos alpina* (L.) Spreng., although it is possible that some *Salix* was present. *Arctostaphylos alpina* is known as a likely mycorrhizal partner for *Hebeloma*, and has been recorded as the only possible mycorrhizal partner on several occasions (see for example Grilli et al. 2020). In the Rocky Mountains, *Hebeloma dunense* was recorded as mycorrhizal with *S. arctica*, *S. planifolia* and *S. reticulata* (Cripps et al. 2019). Beker et al. (2016) found the same pattern for hosts; practically all collections were connected to *Salix* or *Populus* (Salicaceae), and a few with *Dryas* in arctic areas. In Beker et al. (2018), reporting on collections from Svalbard, *H. dunense* was recorded with *Salix* 91% of the time, with *Dryas* 16%, with *Bistorta* 7% (note that more than one possible host was often recorded).

### Hebeloma excedens

Taxon classificationFungiAgaricalesHymenogastraceae

(Peck) Sacc.; Syll. fung. (Abellini) 5: 806, 1887.

DAA74C41-0F9B-5B2E-A8DB-AEE3CF283E75

Fig. 11

#### Macroscopic description.

Cap 1.0–4.0 cm in diameter, shallowly convex, campanulate, then almost applanate, broadly umbonate or not, viscid or greasy, with hygrophanous spots, cinnamon to orange-brown or dark brick in center and pale brown on most of the cap, almost unicolored, with or without velar remnants at margin, or with whitish rim, margin originally described as extending beyond the lamellae, thin-fleshed, universal veil sometimes visible on margin edge, partial veil present. Lamellae sinuate, subdecurrent, narrow, becoming broader and eroded, 3.5–5.5 mm broad, without droplets, very pale, cream with pinkish buff tint, number of lamellae {L} 36–48. Stem 1.5–5.0 × 0.2–0.4 cm, equal, slightly curved, pale cream, silky, pruinose above ring zone, dingier to dark brown below, but still pale with golden brown fibrils in zones, blackening towards base, tough, rubbery. Context whitish in cap and stem apex and yellowish brown in lower stem down to blackish at base. Smell raphanoid, rarely odorless. Spore deposit brownish olive.

**Figure 11.**
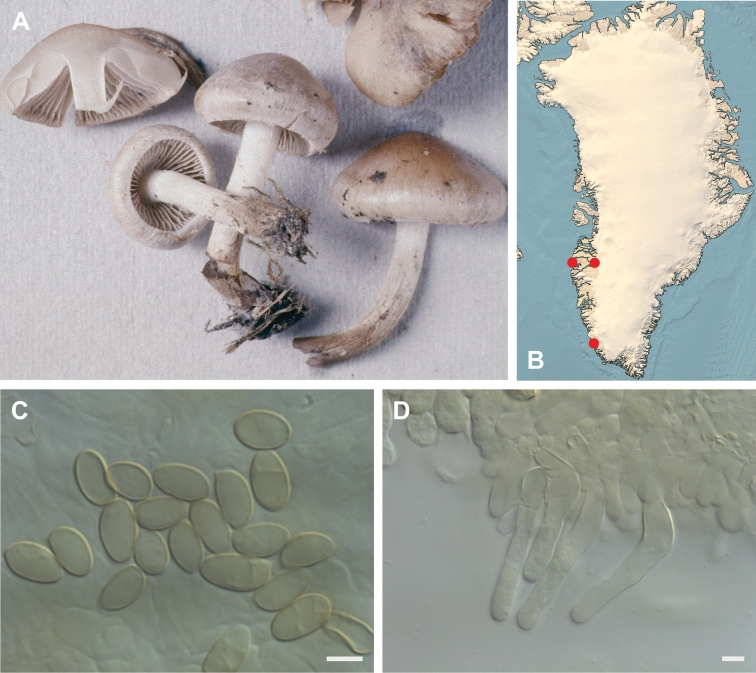
*Hebeloma excedens***A** TB85.238, photograph T. Borgen **B** distribution of cited collections **C** spores ×1600 and **D** cheilocystidia ×1000 of TB85.238 in Melzer’s reagent. Scale bars: 5 µm; microphotographs H.K.J. Beker.

#### Microscopic description.

Spores ellipsoid, a few slightly ovoid, not papillate, 8.5–10.5 × 5–6 µm, ave. Q 1.5–2.0, very weakly ornamented (O1O2), perispore not noticeably loosening (P0), indistinctly dextrinoid (D0D1), pale yellow, rarely yellow brown, ± guttulate. Basidia 20–33 × 6–8 µm, ave. Q = 3.2–4, clavate, mostly four-spored. Cheilocystidia usually lageniform or ventricose, sometimes cylindrical, occasionally geniculate or septate, subcapitate, on ave. 30–55 × 4–6 (apex) × 4–5 (middle) × 7–9 (base) µm, ratios A/M = 0.96–1.23, A/B = 0.60–0.71, B/M = 1.60–1.82. Epicutis an ixocutis, up to 90 µm thick (measured from exsiccata), ixocutis maximum hyphae width up to 6 µm, hyphae rarely encrusted, shape of trama elements beneath subcutis ellipsoid. Caulocystidia similar to cheilocystidia, but usually larger.

#### Collections examined.

**S-Greenland**: Paamiut, 62.01°N, 49.4°W, 23 Aug 1985, T. Borgen (TB85.238, C-F-5073), 10 m, with *Salix glauca*. **W-Greenland**: Kangerlussuaq near the Ice cap, 67.10°N, 50.23°W, 12 Aug 2000, A-M. Larsen, T. Borgen (TB00.086, C-F-103517), 40 m, with *Salix glauca* in a copse. Sisimiut, 1 km N of the village, 66.94°N, 53.67°W, 19 Aug 2000, E. Ohenoja (EO19.8.00.20, OULU F050653), 0 m, in heathland.

#### Distribution.

Known from three localities in southwestern Greenland. Originally described from New York State under *Pinus* and widespread across North America (Cripps et al. 2019). Similar to *H. mesophaeum* and therefore likely to be confused and overlooked. Cripps et al. also describe recent records from alpine Rocky Mountains in Colorado and Montana.

#### Habitat and ecology.

Three collections, two of them with *Salix glauca*. In the Rocky Mountains, *S. planifolia* was the dominant host (Cripps et al. 2019). Two of the localities are on acid soil.

### Hebeloma fuscatum

Taxon classificationFungiAgaricalesHymenogastraceae

Beker & U. Eberh.; Beker, Eberhardt & Vesterholt, Fungi Europ. (Alassio) 14: 133, 2016.

5EF738B4-950A-50B5-BDC5-244B6496A6C3

Fig. 12

#### Macroscopic description.

Cap 0.7–2.5 cm in diameter, convex to umbonate, margin smooth, tacky when moist, not hygrophanous, uniformly colored or more often bicolored, at center sepia to fuscous or dark brick, sometimes with a thin tomentum, at margin dark olive buff to cinnamon or umber; sometimes with remains of universal veil, partial veil present. Lamellae yellowish ochre then brown, emarginate, maximum depth 4 mm, number of lamellae {L} 24–32, droplets absent, white fimbriate edge usually present, but weak. Stem 0.8–7.0 × 0.15–0.5 {median} cm, whitish or pale grayish then browner to gray brown, not darker towards the base, cylindrical, stem Q 4–13, fibrillose, pruinose at apex. Context firm, stem interior stuffed, later hollow, flesh usually discoloring from base. Smell usually raphanoid but sometimes without smell; taste not recorded. Spore deposit color not recorded.

**Figure 12.**
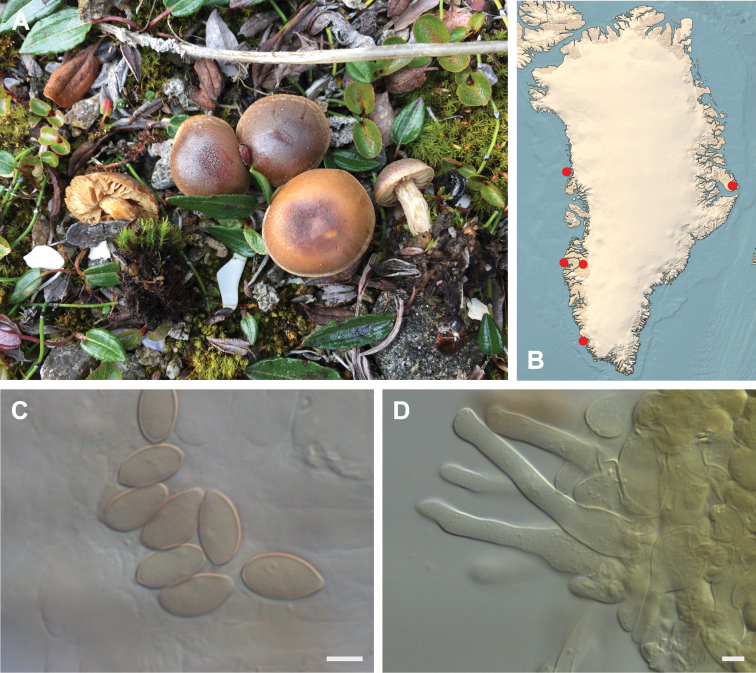
*Hebeloma fuscatum***A** SAE-2016.072, photograph S.A. Elborne **B** distribution of cited collections **C** spores ×1600 and **D** cheilocystidia ×1000 of SAE-2016.072 in Melzer’s reagent. Scale bars: 5 µm; microphotographs H.K.J. Beker.

#### Microscopic description.

Spores amygdaloid, limoniform, occasionally ovoid, papillate, on ave. 12.5–13.5 × 7.0–7.5 µm, ave. Q = 1.6–1.9, brown, guttulate, almost smooth to weakly ornamented (O1O2), perispore not or hardly loosening (P0 (P1)), weakly to rather strongly dextrinoid (D2D3). Basidia 27–33(-36) × 8–9 µm, ave. Q = 3.0–4.1, mostly four-spored. Cheilocystidia usually lageniform or ventricose, occasionally cylindrical, occasionally characteristically with an apical, basal or median thickening, on ave. 41–51 × 4–7 (apex) × 4.5–6 (middle) × 8–13 (base) µm, ratios A/M = 0.9–1.13, A/B = 0.48–0.67, B/M = 1.75–1.97. Epicutis an ixocutis of 30 µm thickness (measured from exsiccata), maximum hyphae width 5–6 µm, sometimes encrusted, shape of trama elements beneath subcutis sausage-shaped and up to 19 µm wide. Caulocystidia similar to cheilocystidia, but less ventricose and very fragile, up to 75 µm long.

#### Collections examined.

**S-Greenland**: Paamiut, Churchyard, 62°N, 49.67°W, 26 Jul 1985, D. Boertmann (DB 85-21, C-F-119783), 25 m, with *Salix glauca*. **W-Greenland**: Kangerlussuaq, SE of Ravneklippen, 67.00°N, 50.67°W, 29 Aug 2018, T. Borgen (TB18.243, C-F-112530), 200 m, with *Salix* sp. in fen. Sisimiut, Præstefjeld, 66.96°N, 53.74°W, 17 Aug 2016, S.A. Elborne (SAE-2016.072-GR, C-F-106737), 300 m, with *Salix herbacea* and *Bistorta vivipara*. Upernavik, 72.79°N, 56.14°W, 18 Aug 2012, D. Boertmann (DB 12.047, C-F-115623), 0 m, in churchyard. **E-Greenland**: Jameson Land, Nerlerit Inaat/Constable Pynt, around the airport, 70.74°N, 22.64°W, 31 Jul 2017, H. Knudsen (HK17.009A, C-F-104897), 50 m. Jameson Land, Nerlerit Inaat/Constable Pynt, delta of Gåseelv valley, 70.77°N, 22.72°W, 11 Aug 2017, S.A. Elborne (SAE-2017.215-GR, C-F-106768), 40 m, with *Salix arctica* along riverside. Jameson Land, Nerlerit Inaat/Constable Pynt, Gåseelv valley, north side, 70.76°N, 22.69°W, 4 Aug 2017, H. Knudsen (HK17.091, C-F-104987), 160 m, with *Salix* sp. in tundra. Jameson Land, Nerlerit Inaat/Constable Pynt, Primulaelv, 70.74°N, 22.67°W, 13 Aug 2017, T. Borgen (TB17C.129, C-F-106783), 180 m, with *Betula* and *Salix* in heathland.

#### Distribution.

Recently described and still only recorded from a few alpine and arctic areas in Europe and arctic Canada (Beker et al. 2016). The Greenland collections are from the Low Arctic zone and High Arctic zone, but the species is rare and scattered. Circumpolar, arctic-alpine.

#### Habitat and ecology.

Eight collections, six with *Salix glauca*, *S. arctica* and *S. herbacea*, two with undetermined *Salix* sp. The presence of *Bistorta* and *Betula* are mentioned, each on one occasion. Soil conditions are variable, but mostly calcareous. *Salix* was the only host mentioned by Beker et al. (2016). Some recent records from subalpine woodlands in central Europe suggest it may also associate with conifers (Grilli et al. 2020).

### Hebeloma grandisporum

Taxon classificationFungiAgaricalesHymenogastraceae

Beker, U. Eberh. & A. Ronikier; Eberhardt, Ronikier, Schütz & Beker, Mycologia 107(6): 1293, 2015.

14615E2C-C095-5AB7-967B-246FAB3F8AF3

Fig. 13

#### Macroscopic description.

Cap up to 1.0 cm in diameter, convex to umbonate, margin usually smooth, but can be crenulate, becoming upturned with age, tacky when moist, with slight spotting, not hygrophanous, uniformly colored or bicolored, at center clay buff or pale yellowish brown or cinnamon, at margin paler, universal veil absent, partial veil present. Lamellae clay color, emarginate to adnate, maximum depth not recorded, number of lamellae {L}18–28; droplets absent, white fimbriate edge indistinct. Stem 1.0–2.0 × 0.2–0.3 cm, ochre-brown, paler in apex, cylindrical, stem Q 6.0–8.5, fibrillose, pruinose at apex. Context firm, stem interior stuffed, stem flesh not discoloring from base. Smell weakly raphanoid or insignificant. Taste not recorded. Spore deposit not recorded.

**Figure 13.**
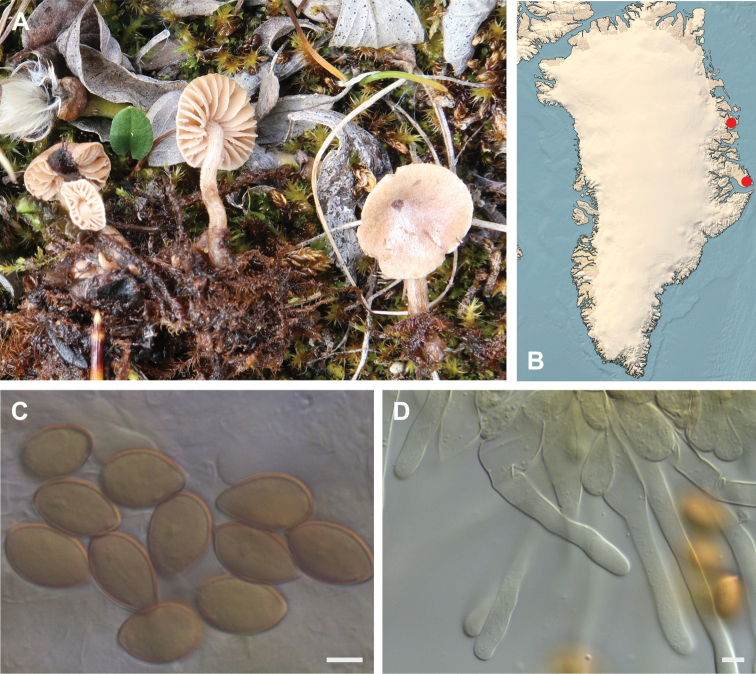
*Hebeloma grandisporum***A** HK17.101, photograph H. Knudsen **B** distribution of cited collections **C** spores ×1600 and **D** cheilocystidia ×1000 of HK17.101 in Melzer’s reagent. Scale bars: 5 µm; microphotographs H.K.J. Beker.

#### Microscopic description.

Spores amygdaloid or limoniform, very strongly papillate, on ave. 14–16 × 8.5–9.5 µm, ave. Q = 1.6–1.8, pale brown to yellow brown, weakly guttulate, almost smooth to weakly ornamented (O1O2), perispore not loosening (P0), distinctly to rather strongly dextrinoid (D2D3). Basidia 23–44 × 6–10 µm, ave. Q = 3.1–4.3, characteristically 2-spored. Cheilocystidia lageniform or ventricose, occasionally clavate-ventricose, characteristically geniculate and septate, on ave. 40–70 × 5.5–6.5 (apex) × 4.5–5.5 (middle) × 8–10.5 (base) µm, ratios A/M = 1.02–1.44, A/B = 0.53–0.75, B/M = 1.48–2.03. Epicutis an ixocutis, up to 130 µm thick (measured from exsiccata), maximum hyphae width 6 µm, ixocutis hyphae sometimes encrusted, shape of trama elements beneath subcutis isodiametric. Caulocystidia similar to cheilocystidia, up to 120 µm long.

#### Collections examined.

**N-Greenland**: Zackenberg, Sydkæret, 74.50°N, 20.75°W, 19 Aug 1999, T. Borgen (TB99.376, C-F-104295), 30 m, with *Salix arctica*. **E-Greenland**: Jameson Land, Nerlerit Inaat/Constable Pynt, west side of Nathorst Fjeld, 70.76°N, 22.64°W, 5 Aug 2017, H. Knudsen (HK17.101, C-F-104997), 40 m, with *Salix* sp. in tundra.

#### Distribution.

Recently described from the southern Carpathians in Romania from a single collection. The two Greenland collections are the first recorded since the type has been published. Both are from the High Arctic zone in Greenland at 70° and 74°N, and it is thus in the northernmost group among the 28 Greenland species. The records are the first from North America and the first outside Europe.

#### Habitat and ecology.

Only two records, both most likely with *Salix arctica* and both on calcareous soil. The only previous collection, the type, grew with *S. retusa* L. at 2270 m a.s.l. in the Carpathians (Eberhardt et al. 2015a). Shiryaev et al. (2018) reported from Zmitrovich and Ezhov (2015) the presence of *Hebeloma gigaspermum* Gröger & Zschiesch. from Franz Josefs Land (Russia). According to Beker et al. (2016), *H. gigaspermum* is most likely a synonym of *H. nauseosum* Sacc. Since neither *H. nauseosum* nor any other species of H. sect. Sacchariolentia occurs in arctic areas, it may be that the large spores and the occurrence in the High Arctic zone rather point to *H. grandisporum*.

#### Notes.

*Hebeloma grandisporum* is one of the most easily recognized among the arctic-alpine *Hebeloma* species by the small basidiomes, the large spores, the relatively few, distant lamellae and large, 2-spored basidia. To date, it is the only *Hebeloma*, of which we are aware, that has consistently 2-spored basidia.

### Hebeloma hygrophilum

Taxon classificationFungiAgaricalesHymenogastraceae

Poumarat & Corriol; Beker, Eberhardt, & Vesterholt, Fungi Europ. (Alassio) 14: 138, 2016.

3355B2B8-0057-52BD-BC96-EB59D9ADB12E

Fig. 14

#### Macroscopic description.

Cap 0.6–3.0 cm in diameter, convex, occasionally umbonate, margin smooth, tacky when moist, not hygrophanous, uniformly colored or bicolored, at center red brown, dark brick, at margin clay-buff, sometimes with whitish fibrils, remains of universal veil variably present, partial veil present. Lamellae at first whitish or pale orange brown, becoming pale cocoa brown, attachment adnate to emarginate, maximum depth not recorded, number of lamellae {L} 23–32; droplets absent, white fimbriate edge usually present, but weak. Stem 2.0–4.3(–5.0) × 0.1–0.45(–0.7) cm, at first whitish, becoming bright orange brown to dirty brown, darker brown towards the base, cylindrical, stem Q (7.1–)10.1–25.8(–35), fibrillose, pruinose to floccose at apex. Context firm, stem interior stuffed, stem flesh discoloring from base. Smell raphanoid, sometimes weakly. Taste mild, raphanoid, later bitter. Spore deposit not recorded.

**Figure 14.**
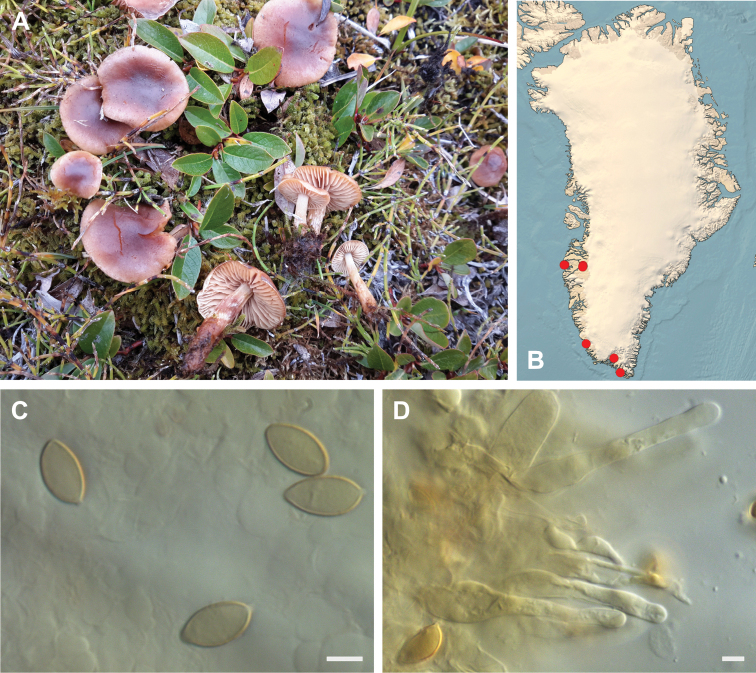
*Hebeloma hygrophilum***A** HK16.195, photograph H. Knudsen **B** distribution of cited collections **C** spores ×1600 and **D** cheilocystidia ×1000 of HK16.195 in Melzer’s reagent. Scale bars: 5 µm; microphotographs H.K.J. Beker.

#### Microscopic description.

Spores shape amygdaloid, fusoid or limoniform, papillate, on ave. 11–13 × 6.0–7.0 µm, ave. Q = 1.7–2.0, brown, guttulate, sometimes weakly, weakly to distinctly ornamented ((O1) O2 (O3)), perispore not or somewhat loosening, (P0P1), distinctly to rather strongly dextrinoid (D2D3). Basidia 27–32 × 7–9 µm, ave. Q = 3.2–4.2, mostly four-spored. Cheilocystidia lageniform or ventricose, occasionally cylindrical, occasionally with characteristic apical or median wall thickening, geniculate or septate (sometimes clamped), 42–64 × 4.5–5.5 (apex) × 4–5 (middle) × 7–11.5 µm, ratios A/M = 0.89–1.24, A/B = 0.36–0.71, B/M = 1.57–2.52. Epicutis an ixocutis, thickness up to 100–130 µm (measured from exsiccata), maximum hyphae width up to 6–7 µm, ixocutis hyphae sometimes encrusted, trama elements beneath subcutis ellipsoid, sausage-shaped, sometimes cylindrical up to 20 µm wide. Caulocystidia cylindrical to ventricose, multi-septate up to 90 µm long.

#### Collections examined.

**S-Greenland**: E of Tasiusaq, 61.15°N, 45.60°W, 20 Aug 1993, E. Rald (ER 93.519, C-F-105494), 10 m, in meadow. S of Tasiusaq, 61.13°N, 45.62°W, 23 Aug 1993, E. Rald (ER 93.425, C-F-104549), 0 m. Paamiut, Taartoq/Mørke Fiord, 62.01°N, 49.26°W, 29 Aug 1998, T. Borgen (TB98.234, C-F-103511), 5 m, with *Salix glauca* in copse. Narsarsuaq, north of Tasiusaq, 60.199186°N, 44.80696°W, 15 Aug 2019, T. Borgen (TB19.052, C-F-137115), 100 m, with *Salix glauca* in moss on streamside. **W-Greenland**: Kangerlussuaq, east of Ravneklippen, 67.01°N, 50.66°W, 24 Aug 2016, S.A. Elborne (SAE-2016.168-GR, C-F-106746), 120 m, with *Salix glauca* along lakeside copse. Kangerlussuaq, Hassells Fjeld, Kløftsøerne, 67.01°N, 50.71°W, 28 Aug 2018, H. Knudsen (HK18.390A, C-F-111118), 300 m, with *Salix glauca* in bog. Kangerlussuaq, Kløftsøerne, 67°N, 50.71°W, 20 Aug 2016, S.A. Elborne (SAE-2016.105-GR, C-F-106741), 300 m, with *Betula nana* and *Bistorta vivipara* along lakeside. Kangerlussuaq, Kløftsøerne, 67.03°N, 50.68°W, 20 Aug 2016, S.A. Elborne (SAE-2016.116-GR, C-F-106742), 300 m, with *Salix glauca* in copse. Kangerlussuaq, Kløftsøerne, 67.03°N, 50.67°W, 10 Aug 2016, H. Knudsen (HK16.064, C-F-104093), 500 m, in bog. Kangerlussuaq, Kløftsøerne, 67.01°N, 50.71°W, 28 Aug 2018, T. Borgen (TB18.236, C-F-112528), 300 m, with *Salix glauca* in copse. Kangerlussuaq, N slope towards Lake Ferguson, 66.95°N, 50.72°W, 29 Aug 2018, S.A. Elborne (SAE-2018.429-GR, C-F-115622), 548 m, with *Salix arctophila* along lakeside. Sisimiut, camping area north of town, 66.94°N, 53.67°W, 20 Aug 2000, S.A. Elborne (SAE-2000.148-GR, C-F-108600), 0 m, with *Salix arctophila* in bog. Sisimiut, near airport, 66.95°N, 53.67°W, 14 Aug 2016, S.A. Elborne (SAE-2016.005-GR, C-F-106735), 10 m, with *Salix* sp. in bog. Sisimiut, north of Alanngorsuaq, 66.95°N, 53.55°W, 15 Aug 2016, S.A. Elborne (SAE-2016.022-GR, C-F-106736), 30 m, with *Salix* sp. in bog. In valley S of Sisimiut behind the dump, 66.93°N, 53.67°W, 18 Aug 2016, H. Knudsen (HK16.195, C-F-108446), 25 m.

#### Distribution.

Found a number of times in southwestern Greenland. Recently described from subalpine areas in the Pyrenees (France) and known from scattered lowland and montane localities in much of Europe. The northernmost locality is from the Boreal zone in Finland (Rovaniemi) at 66.50°N (Beker et al. 2016), which corresponds well in altitude with Kangerlussuaq and Sisimiut (67°N), but in Greenland these areas are Low Arctic. *Hebeloma hygrophilum* was recently recorded from alpine North America (Rocky Mountains, Cripps et al. 2019). Circumpolar, arctic-alpine, boreal zone.

#### Habitat and ecology.

Fifteen collections, ten with *Salix glauca*, *S. arctophila* or unknown *Salix*. One collection with *Betula nana*. Most collections grew in moist habitats often among *Sphagnum* or the moss *Paludella squarrosa* (Hedw.) Brid. The same type of boggy localities with *Salix planifolia* were reported by Cripps et al. (2019) in the Rocky Mountains and by Beker et al. (2016) from Europe, where *Salix aurita*, *S. atrocinerea* and possibly more species of *Salix* act as hosts.

### Hebeloma marginatulum

Taxon classificationFungiAgaricalesHymenogastraceae

(J. Favre) Bruchet; Bull. mens. Soc. linn. Lyon 39(6(Suppl.)): 43, 1970.

E0ABFCD2-FD59-504B-9C4F-6E013C8BBF94

Fig. 15

#### Macroscopic description.

Cap 1.1–4.7 cm in diameter, convex to umbonate, occasionally papillate, margin smooth, tacky when moist, sometimes hygrophanous, uniformly colored or variably bicolored, at center ochraceous or dark olive buff to brownish olive or umber to sepia to fuscous or dark brick, at margin cream to grayish or pinkish buff to ochraceous or dark olive buff to umber, sometimes very thin; with remains of universal veil, partial veil present. Lamellae initially very pale clay, later clay, emarginate, maximum depth 3–8 mm, number of lamellae {L} 32–45, droplets absent, white fimbriate edge usually weakly present. Stem 1.4–4.5(–6.5) × 0.3–0.7(–0.8) cm, pale sordid ochraceous downwards, cylindrical to slightly clavate, fibrillose, whitish pruinose to floccose at apex. Context watery gray brown in cap, in stem lighter, firm, stem interior stuffed, later hollow, occasionally with superior wick, stem flesh usually discoloring from base. Smell usually raphanoid, but sometimes no smell detected. Taste bitter. Spore deposit clay buff to dark olive buff to grayish brown to brownish olive or umber.

**Figure 15.**
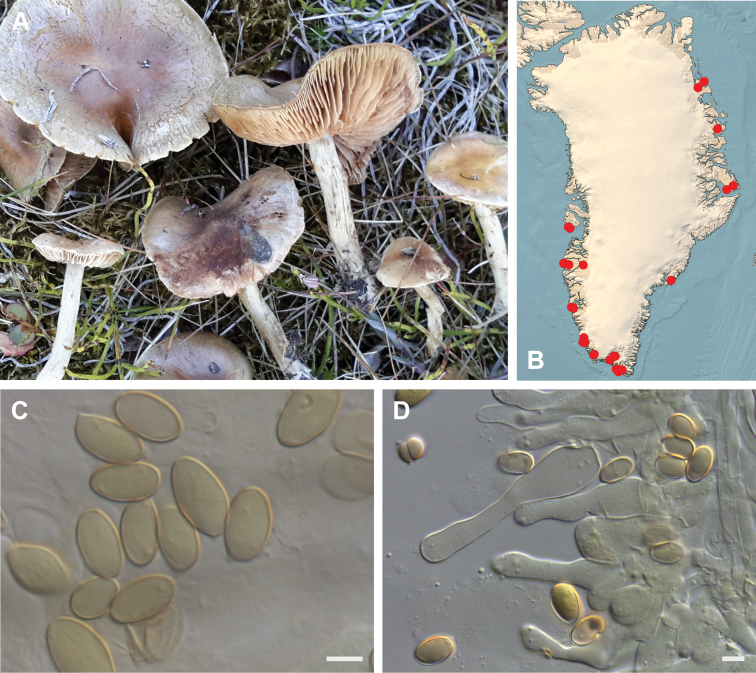
*Hebeloma marginatulum***A** HK16.082, photograph H. Knudsen **B** distribution of cited collections **C** spores ×1600 and **D** cheilocystidia ×1000 of HK16.082 in Melzer’s reagent. Scale bars: 5 µm; microphotographs H.K.J. Beker.

#### Microscopic description.

Spores shape ellipsoid, occasionally amygdaloid or ovoid, on ave. 10.0–13.0 × 5.5–7.5 µm, ave. Q = 1.6–1.9, grayish yellow, rarely guttulate, almost smooth (O1 (O2)), perispore not loosening (P0), not or indistinctly dextrinoid (D0D1). Basidia (28–)30–34(–37) × 7–9 µm, ave. Q = 4–4.5, mostly four-spored. Cheilocystidia lageniform, ventricose or occasionally cylindrical or clavate, occasionally with characteristic apical thickening, bifurcate, geniculate and septate (sometimes clamped), on ave. 44–69 × 5–7 (apex) × 4.5–5 (middle) × 7.5–10.5 (base) µm, ratios A/M = 1.1–1.4, A/B = 0.53–0.81, B/M = 1.55–2.33. Epicutis an ixocutis, 40–100 µm thick (measured from exsiccata), maximum hyphae width 5–6 µm, variably encrusted, trama elements beneath subcutis ellipsoid, sausage-shaped, occasionally pyriform up to 20 µm wide. Caulocystidia similar to cheilocystidia, but many multi-septate up to 150 µm long.

#### Collections examined.

**S-Greenland**: Alluitsoq, 60.32°N, 45.27°W, 21 Jul 2003, H.K.J. Beker (HJB10739), 0 m, with *Betula* sp. and *Salix* sp. in scrubland. Alluitsoq, 60.32°N, 45.27°W, 21 Jul 2003, H.K.J. Beker (HJB 10742), 0 m, with *Betula* sp. and *Salix* sp. in scrubland. Ivittuut, 61.2°N, 48.17°W, 16 Aug 1991, T. Borgen (TB91.198, C-F-103489), 75 m, with *Salix glauca* in copse. Kangilinnguit, bottom of Laksebund, 61.25°N, 48.08°W, 21 Aug 2018, H. Knudsen (HK18.288, C-F-111113), 100 m, in tundra. Nanortalik municipality, Qinngua valley, 60.14°N, 45°W, 22 Jul 2003, H.K.J. Beker (HJB 10730), 100 m, with *Betula pubescens* and *Salix glauca* in woodland. Nanortalik municipality, Qinngua valley, 60.14°N, 45°W, 22 Jul 2003, H.K.J. Beker (HJB 10732), 100 m, with *Betula pubescens* and *Salix glauca* in woodland. Narsaq, 60.91°N, 46.05°W, 13 Aug 1993, E. Rald (ER 93.320, C-F-104552), 20 m, with *Salix glauca* in heathland. Narsaq, 60.91°N, 46.05°W, 2 Aug 1993, E. Rald (ER 93.111, C-F-104312), 20 m, with *Salix glauca* in heathland. Narsaq, 60.91°N, 46.05°W, 2 Aug 1993, E. Rald (ER 93.112, C-F-106421), 20 m. Narsarsuaq, 61.17°N, 45.42°W, 7 Aug 1985, T. Borgen (TB85.036, C-F-5085), 600 m, with *Salix herbacea* in snowbed. NE of Nunatoq, 60.27°N, 44.54°W, 22 Aug 1993, E. Rald (ER 93.589, C-F-104554), 0 m. N of Tasiusaq, across the fjord from Narsarsuaq, 61.17°N, 45.61°W, 5 Aug 1992, E. Rald (ER 92.109, C-F-104304), 150 m. Paamiut, 62.01°N, 49.4°W, 20 Aug 1993, T. Borgen (TB93.139, C-F-103495), 50 m, with *Salix herbacea* in snowbed. Paamiut, 62.01°N, 49.4°W, 23 Aug 1992, T. Borgen (TB92.027, C-F-103493), 10 m, with *Salix arctophila* and *Salix herbacea* in fens. Paamiut, 62.01°N, 49.4°W, 23 Aug 1992, T. Borgen (TB92.028, C-F-103492), 10 m, with *Salix arctophila* and *Salix herbacea* in tundra. Paamiut, 62.01°N, 49.4°W, 8 Aug 1981, T. Borgen (TB81.109, C-F-103553), 10 m, with *Salix herbacea*. Paamiut, 61.99°N, 49.66°W, 5 Sep 1986, T. Borgen (TB86.299, C-F-104300), 25 m. Paamiut, 61.99°N, 49.66°W, 4 Aug 1993, E. Rald (ER 93.181, C-F-104314), 25 m, with *Salix glauca* in heathland. Paamiut, 62°N, 49.4°W, 22 Aug 1986, T. Borgen (TB86.247, C-F-103467), 20 m, with *Salix herbacea* and *Bistorta vivipara*. Paamiut Cemetery, 62.01°N, 49.4°W, 19 Aug 1984, T. Borgen (TB84.151, C-F-103538), 10 m, with *Salix arctophila*. Paamiut, Kangerluarsuk S, 62.28°N, 49.58°W, 31 Aug 1995, B. Knudsen (TB95.068, C-F-103505), 150 m. Paamiut, Kangilineq/Kvaneøen, 61.95°N, 49.47°W, 20 Aug 2008, T. Borgen (TB08.146, C-F-106750), 30 m, with *Salix glauca*. Qassiarsuk, Tasiusaq, 61.15°N, 45.52°W, 9 Aug 1992, E. Rald (ER 92.181, C-F-104306), 25 m. Qassiarsuk, Tasiusaq, 61.15°N, 45.52°W, 9 Aug 1992, E. Rald (ER 92.180, C-F-104305), 25 m. Tasiusaq, 61.14°N, 45.63°W, 28 Jul 1993, E. Rald (ER 93.021, C-F-104319), 20 m, with *Salix glauca* in heathland. **W-Greenland**: Disko, Fortune Bay, 69.31°N, 53.88°W, 2 Aug 1986, T. Borgen (TB86.119, C-F-103566), 20 m. Qeqertarsuaq/Disko, Godhavn, 69.24°N, 53.54°W, 28 Jul 1986, T. Borgen (TB86.060, C-F-6922), 0 m. Disko, Qeqertarsuaq, 69.25°N, 53.55°W, 27 Jul 1986, T. Borgen (TB86.072, C-F-5090), 0 m. Kangerluarsunguaq/Kobbefjord, just south of the field station, 64.14°N, 51.39°W, 11 Aug 2009, T. Borgen (TB09K017, C-F-106753), 20 m, with *Salix herbacea* in snowbed. Kangerlussuaq, NE facing slopes along Lake Ferguson, 66.10°N, 50.61°W, 12 Aug 2016, H. Knudsen (HK16.082, C-F-104111), 300 m. Nuuk, airport area, 64.19°N, 51.67°W, 14 Aug 2008, T. Borgen (TB08.126, C-F-106749), 100 m, with *Salix herbacea* in snowbed. Sisimiut, from airport and round the mountain, 66.95°N, 53.72°W, 17 Aug 2016, H. Knudsen (HK16.181, C-F-108419), 30 m. Sisimiut, from airport and round the mountain, 66.95°N, 53.72°W, 17 Aug 2016, H. Knudsen (HK16.180, C-F-108418), 30 m. Sisimiut, into the E valley, 66.90°N, 52.86°W, 15 Aug 2016, H. Knudsen (HK16.134, C-F-104164), 0 m, with *Salix herbacea* and *Salix arctica*. Sisimiut, south of town, 66.93°N, 53.65°W, 18 Aug 2016, S.A. Elborne (SAE-2016.085-GR, C-F-106738), 20 m, with *Salix herbacea* in snowbed. **N-Greenland**: NE of Annekssø, 77.42°N, 21.33°W, 20 Jul 1990, B. Fredskild (BF 90 loc. 5, C-F-5113), 150 m. Stormlandet, N of Depotnæs, 77.6°N, 18.95°W, 20 Aug 1990, B. Fredskild (BF 90 loc. 8, C-F-5147), 20 m. Zackenberg, 74.5°N, 21°W, 14 Aug 2006, T. Borgen (TB06.170, C-F-119778) ,35 m, with *Dryas* sp. and *Salix* sp. in scrubland. Zackenberg, 74.5°N, 21°W, 5 Aug 2006, T. Borgen (TB06.090, C-F-119779), 30 m, with *Dryas* sp. and *Salix arctica* in grassland. Zackenberg, near Teltdammen, 74.5°N, 21°W, 5 Aug 2006, T. Borgen (TB06.090, C-F-119747), 30 m, with *Dryas* sp. and *Salix arctica* in scrubland. Zackenberg, shortly SW of Kamelen, 74.5°N, 21°W, 11 Aug 2006, T. Borgen (TB06.158, C-F-119775), 40 m, with *Salix arctica* and *Bistorta vivipara* in grassland. **E-Greenland**: Jameson Land, Constable Pynt, 70.75°N, 22.67°W, 30 Jul 1988, D. Boertmann (DB GR 88-40, C-F-3453), 2 m, with *Salix arctica*. Jameson Land, Constable Pynt, delta of Jyllandselv, 70.68°N, 24.08°W, 28 Jul 1988, D. Boertmann (DB GR 88-33, C-F-3448), 10 m, with *Salix herbacea*. Jameson Land, Nerlerit Inaat/Constable Pynt, delta of Gåseelv valley, 70.76°N, 22.65°W, 7 Aug 2017, H. Knudsen (HK17.149, C-F-105051), 40 m. Kuummiut, Torsukattak, 65.87°N, 37.01°W, 2 Aug 2008, T. Borgen (TB08.035, C-F-101622), 35 m.

#### Distribution.

*Hebeloma marginatulum* is widespread and, apparently, one of the most common *Hebeloma* species in Greenland (11.1% of the collections, see Table 3), also common in other parts of North America and present in parts of Europe (Beker et al. 2016; Cripps et al. 2019). It is a truly arctic-alpine species hardly found outside these areas. Arctic-alpine.

#### Habitat and ecology.

Forty-five collections with many hosts. Twenty collections with *Salix* (*S. herbacea* 9, *S. glauca* 6, *S. arctophila* 3 and *S. arctica* 2), two with Betula pubescens var. pumila, two with *Betula* sp. and three with *Dryas octopetala* and *D. integrifolia* (as always, other potential hosts may have been nearby). In the Rocky Mountains, it is also common, found with *Salix arctica*, *S. reticulata* and *S. planifolia* (Cripps et al. 2019). Beker et al. (2016) have *S. herbacea* and *S. polaris* as the main hosts. *Hebeloma marginatulum* occurs on a variety of soil types seemingly without specific preferences.

### Hebeloma mesophaeum

Taxon classificationFungiAgaricalesHymenogastraceae

(Pers.) Quél.; Mém. Soc. Émul. Montbéliard, Sér. 2, 5: 128, 1872.

98DD92A8-6BFC-5DB1-8F04-51A90BF40021

Fig. 16

#### Macroscopic description.

Cap 0.7–6.5 cm in diameter, convex when young, usually becoming umbonate, sometimes papillate, margin sometimes involute when young, occasionally crenulate, often eroded, upturned or radially split when older, tacky when moist, not hygrophanous, usually bicolored, rarely unicolored, at center dark pinkish buff to dark olive buff to grayish brown to umber or brownish olive to sepia to clay-buff or cinnamon or yellowish brown to orange brown or dark brick, at margin cream to pinkish buff to grayish buff or clay-buff to dark olive buff to grayish brown or brownish olive to grayish pink, with remains of universal veil, partial veil present. Lamellae when young pale brown, when older ochre brown to fairly dark brown, adnate to emarginate, sometimes with decurrent tooth, rarely decurrent, maximum depth up to 6.5 mm, number of lamellae {L} 30–48, droplets absent, but occasionally visible with × 10 lens or rarely visible with naked eye, white fimbriate edge usually present, sometimes weak. Stem 1.4–8.0(–9.0) × (0.2–)0.3–0.6(–0.8) cm, whitish then pale sordid gray brown, already when young dark sordid brown at base, when young with brownish tomentose velar remnants downwards, cylindrical, rarely tapering or clavate, stem Q (4.3–)4.8–16.3(–18.8), fibrillose, usually pruinose to floccose at apex. Context firm, stem interior stuffed, later hollow, occasionally with superior wick, stem flesh discoloring from base, sometimes very strongly, rarely absent. Smell usually raphanoid, rarely absent. Taste mild to bitter, sometimes raphanoid. Spore deposit dark olive buff to brownish olive to umber.

**Figure 16.**
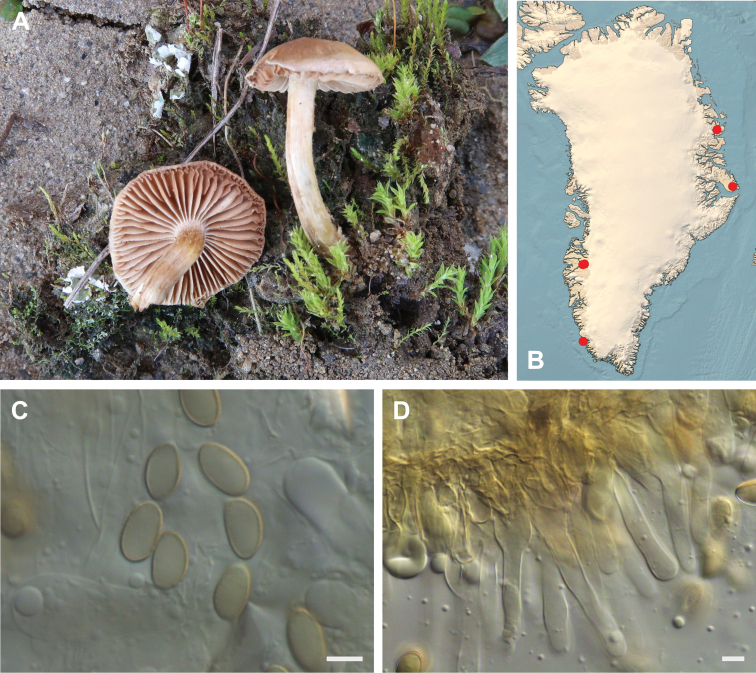
*Hebeloma mesophaeum***A** HK17.020, photograph H. Knudsen **B** distribution of cited collections **C** spores ×1600 and **D** cheilocystidia ×1000 of HK17.020 in Melzer’s reagent. Scale bars: 5 µm; microphotographs H.K.J. Beker.

#### Microscopic description.

Spores ellipsoid, occasionally ovoid, on ave. 8.0–10.5 × 4.5–6.0 µm, Q = 1.3–1.9, (but see the notes below) yellow through yellow brown to brown, guttulation variable, almost smooth to weakly ornamented (O1O2), perispore not or somewhat loosening (P0P1), indistinctly dextrinoid (D0D1). Basidia 24–31(–32) × 7–9 µm; ave. Q = 3.2–4, mostly four-spored. Cheilocystidia lageniform, ventricose, occasionally cylindrical or rarely clavate, occasionally with characteristic apical or basal wall thickening, sometimes bifurcate, geniculate or septate (sometimes clamped), on ave. 26–62 × 4–6 (apex) × 3.5–6 (middle) × 6–11 (base) µm, ratios A/M = 0.94–1.25, A/B = 0.45–0.98, B/M = 1.36–2.19. Epicutis an ixocutis, 60–350 µm thick (measured from exsiccata), maximum hyphae width 3–10 µm, encrustation variable, trama elements beneath subcutis angular or isodiametric to ellipsoid or cylindrical to sausage-shaped up to 22 µm wide. Caulocystidia similar to cheilocystidia, but less ventricose, up to 130 µm long.

#### Collections examined.

**S-Greenland**: Paamiut, 62°N, 49.67°W, 6 Sep 1990, T. Borgen (TB90.086, C-F-76757), 20 m, with *Salix glauca*. Paamiut, 62.01°N, 49.67°W, 17 Aug 1990, T. Borgen (TB90.040, C-F-119759), 30 m, with *Bistorta vivipara*. **W-Greenland**: Kangerlussuaq, 67.01°N, 50.72°W, 2 Aug 2016, T. Borgen (TB16.038, C-F-104301), 50 m, with *Salix glauca* in tundra. Kangerlussuaq NE, near a glacier, 67.02°N, 50.66°W, 12 Aug 2000, E. Ohenoja (EO12.8.00.1, OULU F050224), 40 m, in heathland. Kangerlussuaq, Sandflugtsdalen, near the ice cap, 67.0578°N, 50.4571°W, 12 Aug 2000, T. Borgen (TB00.088, C-F-137116), 50 m. Kangerlussuaq, Sandflugtsdalen, 67.06°N, 50.46°W, 12 Aug 2000, T. Borgen (TB00.093, C-F-103521), 50 m, with *Salix glauca* in dunes. Kangerlussuaq, Sandflugtsdalen, 67.06°N, 50.46°W, 12 Aug 2000, T. Borgen (TB00.094, C-F-103522), 200 m, with *Salix glauca* in dunes. Kangerlussuaq, Sandflugtsdalen, 67.06°N, 50.46°W, 12 Aug 2000, A-M. Larsen, T. Borgen (TB00.091, C-F-103520), 50 m, with *Salix glauca* in dunes. Kangerlussuaq, Sandflugtsdalen, c. 15 km E of airport, 67.06°N, 50.46°W, 2 Aug 2016, T. Borgen (TB16.040G, C-F-103578), 50 m, with *Salix glauca* in dunes. **N-Greenland**: Zackenberg, S of the Station, towards Zackenberg River, 74.5°N, 21°W, 10 Aug 1999, T. Borgen (TB99.264, C-F-104297), 50 m, with *Dryas* sp. in scrubland. **E-Greenland**: Jameson Land, Nerlerit Inaat/Constable Pynt, Primulaelv, 70.74°N, 22.67°W, 1 Aug 2017, H. Knudsen (HK17.020, C-F-104908), 180 m.

#### Distribution.

*Hebeloma mesophaeum* is widely distributed, but apparently uncommon in Greenland with only 11 records (2.9%). This is in contrast to the frequency in Europe, where it is very common and widely distributed (Beker et al. 2016). From alpine and arctic Europe, it is known from the Alps, Southern Carpathians, Svalbard and Iceland. Outside Europe and Greenland, there are a few records from the Rocky Mountains (Colorado, Cripps et al. 2019) and arctic Canada (Ohenoja and Ohenoja 2010). It is distributed from the Mediterranean area through the Temperate and Boreal zone to the Arctic and Alpine zones, being less common in the colder areas. Circumpolar, arctic-alpine, temperate, boreal and subarctic.

#### Habitat and ecology.

Eleven collections, six of them with *Salix glauca*, one with *Dryas octopetala* and *D. integrifolia*, one with *Bistorta*, three not reported. One record from the Rocky Mountains was also with *S. glauca*. The soil conditions were variable. *Hebeloma mesophaeum* is widespread and very versatile when it comes to hosts, including many deciduous as well as coniferous hosts (Beker et al. 2016).

#### Notes.

In Beker et al. (2016), it was reported that in arctic and alpine areas there is a chance of confusion with *H. marginatulum*, due to the fact that sometimes *H. mesophaeum*, growing in such habitats, tends to have larger spores. They reported that all the ‘large-spored’ collections were from Iceland and Svalbard. In Greenland, we have also found collections with larger spores; indeed, nine of the eleven collections discussed here exhibited larger spores in a range from 10.3–11.7 × 6.4–7.1 µm, a similar range to that reported for Svalbard and Iceland. At the time, Beker et al. (2016) did consider creating a variety to address these collections but decided such a course of action was premature and that further study of this variation was required. With regard to the confusion with *H. marginatulum*, the strongly bicolored cap of *H. mesophaeum* should normally aid identification.

### Hebeloma nigellum

Taxon classificationFungiAgaricalesHymenogastraceae

Bruchet; Bull. mens. Soc. linn. Lyon 39(6(Suppl.)): 126, 1970.

CD279D1E-BC2E-561E-A952-F07898D07E26

Fig. 17

#### Macroscopic description.

Cap 0.7–3.4 cm in diameter, convex to umbonate, occasionally papillate, margin involute, tacky when moist, unicolored or usually bicolored, at center umber to dark brick or snuff brown, at margin cream to pinkish buff, occasionally with remains of universal veil, partial veil present. Lamellae at maturity brownish clay, adnate to emarginate, maximum depth 2.5–6 mm, number of lamellae {L} 22–36, droplets absent, white fimbriate edge present, sometimes weakly. Stem 1.4–5.0 × 0.15–0.4 cm, whitish pruinose at apex, initially whitish lengthily fibrillose, downward, sordid brown, cylindrical, stem Q (6.5–)7.5–12.5(–18.7), fibrillose, pruinose to floccose at apex. Context firm, stem interior stuffed later hollow, flesh discoloring from base, sometimes very strongly. Smell usually raphanoid, sometimes none. Taste bitter, raphanoid. Spore deposit umber.

**Figure 17.**
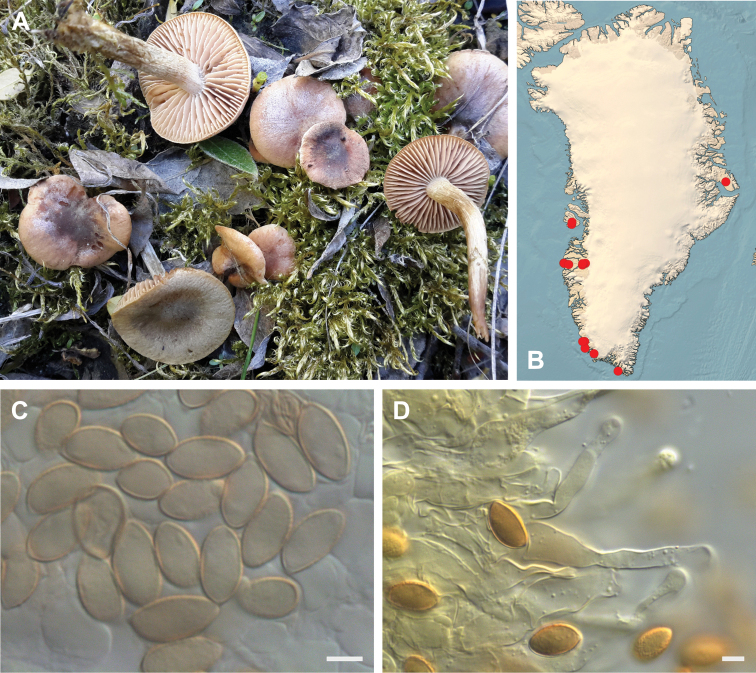
*Hebeloma nigellum***A** HK16.033, photograph H. Knudsen **B** distribution of cited collections **C** spores ×1600 and **D** cheilocystidia ×1000 of TB85.249 in Melzer’s reagent. Scale bars: 5 µm; microphotographs H.K.J. Beker.

#### Microscopic description.

Spores mainly amygdaloid, sometimes ellipsoid to ovoid, on ave. 11.0–13.5 × 7.0–7.5 µm, ave. Q = 1.5–1.9, yellow through yellow brown to brown, guttulation variable, almost smooth to slightly ornamented (O1O2), perispore not or very slightly loosening (P0 (P1)), rather strongly dextrinoid ((D2) D3). Basidia 25–39(–45) × 7–10(–11) µm, ave. Q = 3.1–3.9, mostly four-spored. Cheilocystidia usually lageniform, ventricose, occasionally cylindrical, occasionally with characteristic apical wall thickening, geniculate or septate (sometimes clamped), 44–65 × 4.5–6.5 (apex) × 4–5.5 (middle) × 6.5–11 (base) µm, ratios A/M = 0.92–1.41, A/B = 0.45–0.89, B/M = 1.45–2.3. Epicutis an ixocutis, 40–75 µm thick (measured from exsiccata), maximum hyphae width 5–6.5 µm, sometimes encrusted, trama elements beneath subcutis ellipsoid, polygonal, sausage-shaped up to 15 µm wide. Caulocystidia rather irregular, similar to cheilocystidia, up to 140 µm long.

#### Collections examined.

**S-Greenland**: Kangilinnguit, bottom of Laksebund, 61.25°N, 48.08°W, 16 Aug 2018, H. Knudsen (HK18.199, C-F-111110), 100 m, with *Alnus alnobetulae* in scrubland. Nanortalik municipality, Qinngua valley, 60.14°N, 45°W, 6 Aug 1991, T. Borgen (TB91.080, C-F-103490), 250 m, with *Salix glauca* in copse. Paamiut, 62.01°N, 49.4°W, 31 Aug 1986, T. Borgen (TB86.276, C-F-103572), 10 m. Paamiut, 62.01°N, 49.4°W, 14 Aug 1990, T. Borgen (TB90.035, C-F-103479), 10 m. Paamiut, 62.01°N, 49.4°W, 1 Sep 1990, T. Borgen (TB90.073, C-F-103541), 10 m, with *Salix arctophila* along riverside. Paamiut, Kangilineq, Kvaneøen, 61.95°N, 49.47°W, 26 Aug 1984, T. Borgen (TB84.183, C-F-103468), 10 m, with *Salix arctophila*. Paamiut, Kangilineq/Kvaneøen, 61.57°N, 49.28°W, 25 Aug 1985, T. Borgen (TB85.249, C-F-103529), with *Salix*. Paamiut, Kangilineq/Kvaneøen, 61.99°N, 49.66°W, 26 Aug 1990, T. Borgen (TB90.056, C-F-103542), 15 m, with *Salix arctophila* in fenland. **W-Greenland**: Disko, N end of Blæsedalen, 69.5°N, 53.32°W, 25 Jul 1986, T. Borgen (TB86.052, C-F-119739), 300 m, with *Salix herbacea* in snowbed. Kangerlussuaq, Ammaloortup Nunaa W of Lake Ferguson, 66.99°N, 50.61°W, 8 Aug 2016, H. Knudsen (TB16.086G, C-F-103583), 275 m, with *Salix glauca*, *Betula nana* and *Sphagnum* along streamside. Kangerlussuaq, Kløftsøerne, 67.06°N, 50.68°W, 13 Aug 2016, H. Knudsen (HK16.110, C-F-104140), 500 m, with *Salix glauca*. Kangerlussuaq, Lake Ferguson, Tasersuatsiaq, 66.97°N, 50.70°W, 22 Aug 2016, S.A. Elborne (SAE-2016.131-GR, C-F-106743), 100 m, with *Salix glauca* in copse. Kangerlussuaq, NE facing slopes along Lake Ferguson, 66.99°N, 50.61°W, 8 Aug 2016, H. Knudsen (HK16.033, C-F-104063), 300 m, with *Salix glauca* in riverbed. Kangerlussuaq, outlet of Lake Ferguson, 66.99°N, 50.61°W, 6 Aug 2016, T. Borgen (TB16.060G, C-F-103580), 275 m, with *Salix glauca* in scrubland. Kangerlussuaq, W Bridge near Ice cap, 67.09°N, 50.23°W, 2 Aug 2016, T. Borgen (TB16.035G, C-F-103577), 50 m, with *Salix glauca* at lakeside. NW of Nasaassaq, E-valley, E of Sisimiut, 66.93°N, 53.61°W, 18 Aug 2000, E. Horak (ZT9139, ZT9139), 50 m, with *Betula nana*, *Salix glauca* and *Salix herbacea*. Qeqertarsuaq/Disko, Godhavn area, 69.65°N, 53.32°W, 26 Jul 1986, T. Borgen (TB86.065, C-F-119791), 450 m. Sisimiut, 1 km north of the village, 66.94°N, 53.67°W, 19 Aug 2000, E. Ohenoja (EO19.8.00.20, OULU F050653), 0 m, in heathland. Sisimiut, in the E valley, 66.89°N, 52.86°W, 15 Aug 2016, H. Knudsen (HK16.133, C-F-104163), 0 m, with *Salix herbacea*. Sisimiut, Kællingehætten, 66.93°N, 53.59°W, 16 Aug 2016, H. Knudsen (HK16.165A, C-F-108402), 400 m. **E-Greenland**: Jameson Land, Constable Pynt, Draba Sibirica Elv, 71.15°N, 23.57°W, 28 Jul 1983, D. Boertmann (DB GR 83-80, C-F-119734), 0 m, with *Salix arctica*.

#### Distribution.

*Hebeloma nigellum* is common and widespread in Greenland and present in other parts of North America (Cripps et al. 2019). It is widespread in temperate Europe, but more commonly in arctic-alpine areas (Beker et al. 2016). Circumpolar.

#### Habitat and ecology.

Twenty-one collections, and where the host was specified, 15 were recorded with *Salix* (*S. glauca* 8, *S. arctophila* 3, *S. herbacea* 3, *S. arctica* 1), and one recorded with Alnus alnobetulae ssp. crispa. The majority are from riversides, lakesides, fens and snowbeds, but also in humid places with *S. arctophila*. Most localities are on neutral ground, but it is found in acid localities as well. In the Rocky Mountains, it was recorded with *Salix planifolia* (Cripps et al. 2019). From lowland Europe Beker et al. 2016 noted *S. aurita* and *S. cinerea* as hosts.

### Hebeloma oreophilum

Taxon classificationFungiAgaricalesHymenogastraceae

Beker & U. Eberh.; Eberhardt, Ronikier, Schütz & Beker, Mycologia 107(6): 1295, 2016 (“2015”).

1A27847C-BE7C-5F14-9F8F-7DF06B1123AA

Fig. 18

#### Macroscopic description.

Cap 1.0–3.8 cm in diameter, convex to umbonate, sometimes strongly, margin smooth, often involute when young, tacky when moist, usually not hygrophanous, but we have occasionally seen collections that are, unicolored or variably two-colored, at center dark olive buff to brownish olive to umber to cinnamon or sepia to brick to dark brick to fuscous, sometimes cracked, at margin cream to pinkish buff to clay-pink, sometimes margin very thin, usually pruinose to tomentose, usually with remains of universal veil, partial veil present. Lamellae whitish then pale clay brown, emarginate to broadly adnexed, maximum depth 3–8 mm, number of lamellae {L} 40–48, droplets absent or very small (lens), white fimbriate edge sometimes present. Stem 1.5–7.0 × 0.3–0.65 {median} × (0.3–1.0) {base} cm, at first whitish later brown downwards, apically with whitish fibrillose ring zone, sometimes with indistinct belts, downwards with suggestion of a fibrillose ring, pale sordid brown, pale watery brown, downwards dark brown when rubbed, cylindrical, rarely clavate, stem Q (4.2–)5.5–8.7(–15), pruinose at apex. Context firm, stem interior stuffed, later hollow, stem flesh discoloring from base usually. Smell raphanoid. Taste raphanoid, slightly bitter. Spore deposit brownish olive.

**Figure 18.**
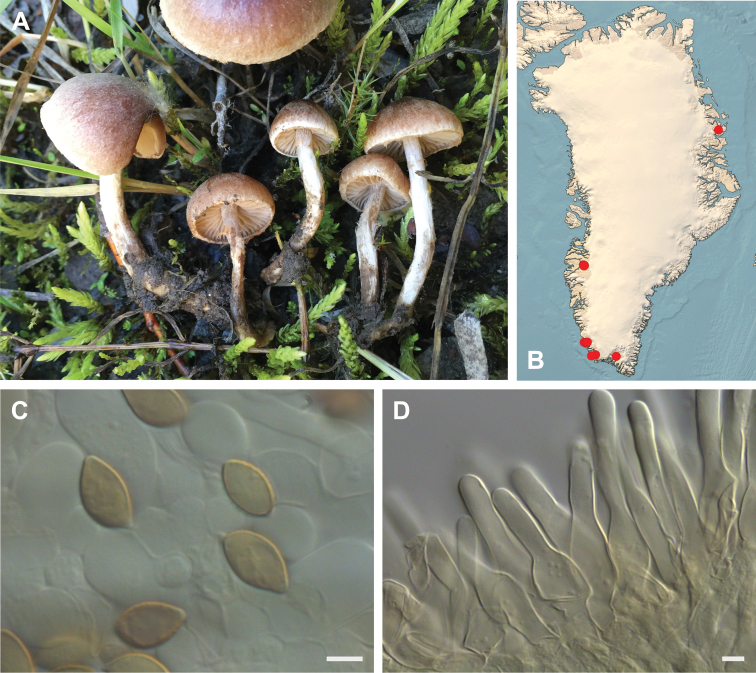
*Hebeloma oreophilum***A** SAE-2016.134, photograph S.A. Elborne **B** distribution of cited collections **C** spores ×1600 and **D** cheilocystidia ×1000 of HK18.401 in Melzer’s reagent. Scale bars: 5 µm; microphotographs H.K.J. Beker.

#### Microscopic description.

Spores amygdaloid, occasionally ovoid or limoniform, usually weakly papillate, on ave. 11.0–14.0 × 6.5–7.5 µm, ave. Q = 1.6–2.1, yellow brown through brown, guttulation variable, almost smooth to weakly ornamented (O1O2), perispore not or somewhat loosening (P0P1), distinctly to rather strongly dextrinoid (D2D3). Basidia (25–)26–33(–36) × 7–9(–11) µm, ave. Q = 3.3–4.9, mostly four-spored. Cheilocystidia lageniform, ventricose, occasionally cylindrical, occasionally with characteristic apical, median or basal wall thickening, geniculate or septate, 42–57 × 4–6 (apex) × 4–6 (middle) × 8–10 (base) µm, sometimes with yellowish contents, ratios A/M 1.03–1.24, A/B = 0.44–0.67, B/M = 1.57–2.4. Epicutis an ixocutis, 40–70 µm thick (measured from exsiccata), maximum hyphae width 5–7 µm, without encrustations, trama elements beneath subcutis cylindrical to sausage-shaped up to 18 µm wide. Caulocystidia similar to cheilocystidia but less ventricose more cylindrical, often multi-septate, up to 120 µm long.

#### Collections examined.

**S-Greenland**: Kangilinnguit, 61.14°N, 48.6°W, 20 Aug 1991, T. Borgen (TB91.233, C-F-103487), 25 m, with *Alnus alnobetulae*. Kangilinnguit at Grønnedal Hut, 61.23°N, 48.08°W, 15 Aug 1985, T. Borgen (TB85.180, C-F-119740), 350 m, with *Salix arctophila* in *Sphagnum*. Narsarsuaq, outer part of Hospital Valley, 61.17°N, 45.43°W, 9 Aug 1985, T. Borgen (TB85.061, C-F-103590), 50 m, with *Betula pubescens* and *Salix glauca*. Paamiut, 62.01°N, 49.4°W, 12 Aug 1990, T. Borgen (TB90.010, C-F-103558), 25 m, with *Salix arctophila* and *Salix herbacea* in snowbed. Paamiut, 62.01°N, 49.4°W, 14 Aug 1990, T. Borgen (TB90.036, C-F-103480), 10 m, with *Salix glauca*. Paamiut, 62.01°N, 49.4°W, 1 Sep 1986, T. Borgen (TB86.292, C-F-103588), 100 m. Paamiut, 62.01°N, 49.4°W, 5 Aug 1998, T. Borgen (TB98.073, C-F-103509), 35 m, with *Salix herbacea* in fen. Paamiut, “Peters Fjeld”, 62.01°N, 49.4°W, 31 Aug 1983, T. Borgen (TB83.035, C-F-103472), 10 m, with *Salix arctophila* in *Sphagnum*. Paamiut, head of Eqaluit, median part, 62.03°N, 49.25°W, 15 Aug 1998, T. Borgen (TB98.120, C-F-103510), 300 m, with *Betula glandulosa* and *Salix glauca* in heathland. Paamiut, Kangilineq /Kvaneøen, 61.95°N, 49.47°W, 6 Sep 1984, T. Borgen (TB84.215, C-F-104298), 20 m, with *Salix herbacea* and *Bistorta vivipara* in snowbed. Paamiut, Kangilineq/Kvaneøen, 61.99°N, 49.66°W, 27 Aug 1984, T. Borgen (TB84.184, C-F-103539), 15 m, with *Salix glauca*. **W-Greenland**: Kangerlussuaq, Kløftsøerne, 67.03°N, 50.80°W, 31 Jul 2016, T. Borgen (TB16.017G, C-F-103576), 300 m, with *Betula nana* and *Salix glauca*, in *Sphagnum* in scrubland. Kangerlussuaq, Lake Ferguson, Tasersuatsiaq, 66.97°N, 50.70°W, 22 Aug 2016, S.A. Elborne (SAE-2016.134-GR, C-F-106744), 100 m, with *Salix glauca* in copse. Kangerlussuaq, NE facing slopes along Lake Ferguson, 66.99°N, 50.61°W, 12 Aug 2016, H. Knudsen (HK16.091, C-F-104120), 300 m, in *Sphagnum* sp. Kangerlussuaq, slopes SW of Lake Ferguson, 66.96°N, 50.69°W, 29 Aug 2018, H. Knudsen (HK18.401, C-F-111120), 380 m, in tundra. **N-Greenland**: Zackenberg, 74.5°N, 21°W, 17 Aug 2006, T. Borgen (TB06.225, C-F-119772), 20 m, with *Salix arctica* in scrubland. Zackenberg, Sydkæret, 74.5°N, 21°W, 19 Aug 1999, T. Borgen (TB99.374, C-F-119758), 30 m, *with Salix arctica* in scrubland. Zackenberg, Sydkæret, 74.5°N, 21°W, 5 Aug 2006, T. Borgen (TB06.098, C-F-119773), 20 m, in fen.

#### Distribution.

Widely distributed and apparently common in Greenland, but never recorded from the East coast (only north east, see habitat). Described recently (Eberhardt et al. 2015a) from an alpine site (1970 m) in Slovakia and still only known from few countries. In Europe, it is recorded from northern Finland, from the Subarctic and Arctic zone and from the High Arctic zone on Svalbard. Outside Europe, it is known from low alpine and alpine sites in the Rocky Mountains and Canada (Beker et al. 2016, Cripps et al. 2019). Circumpolar, arctic-alpine to subarctic.

#### Habitat and ecology.

Eighteen collections, six recorded with *Salix glauca*, four with *S. arctica*, three with *S. arctophila*, two with *S. herbacea* and one recorded under Alnus alnobetulae ssp. crispa. The absence from the calcareous, well-investigated Jameson Land on the east coast may signal a preference for more acid soil types. In the low alpine Rocky Mountains, Cripps et al. (2019) found it a number of times with *Salix planifolia*, *S. arctica* and *S. glauca*.

### Hebeloma pubescens

Taxon classificationFungiAgaricalesHymenogastraceae

Beker & U. Eberh.; Beker, Eberhardt & Vesterholt, Fungi Europ. (Alassio) 14: 173, 2016.

ECA2AA6D-0C09-5951-96AA-A9227BEC5AE1

Fig. 19

#### Macroscopic description.

Cap 1.2–2.4 cm in diameter, convex to umbonate, sometimes papillate, margin smooth, tacky when moist, not hygrophanous, unicolored or variably bicolored, at center grayish brown to yellowish brown or umber, tomentose, usually covered, particularly at the margin, with soft hairs, at margin dark grayish buff, sometimes with remains of universal veil, partial veil present. Lamellae light brown, later slightly darker, emarginate, maximum depth 2.5–5 mm, number of lamellae {L} 25–32, droplets absent, white fimbriate edge weakly present. Stem 1.1–4.6 × 0.2–0.4 cm, whitish pale floccose in apex, downwards lengthily fibrillose, sordid brown or very pale ochraceous, cylindrical, stem Q (3.7–)4.8–8.3(–9.7), fibrillose. Context firm, stem interior stuffed, later hollow, flesh usually discoloring from base. Smell raphanoid, sometimes strongly. Taste mild. Spore deposit brownish olive.

**Figure 19.**
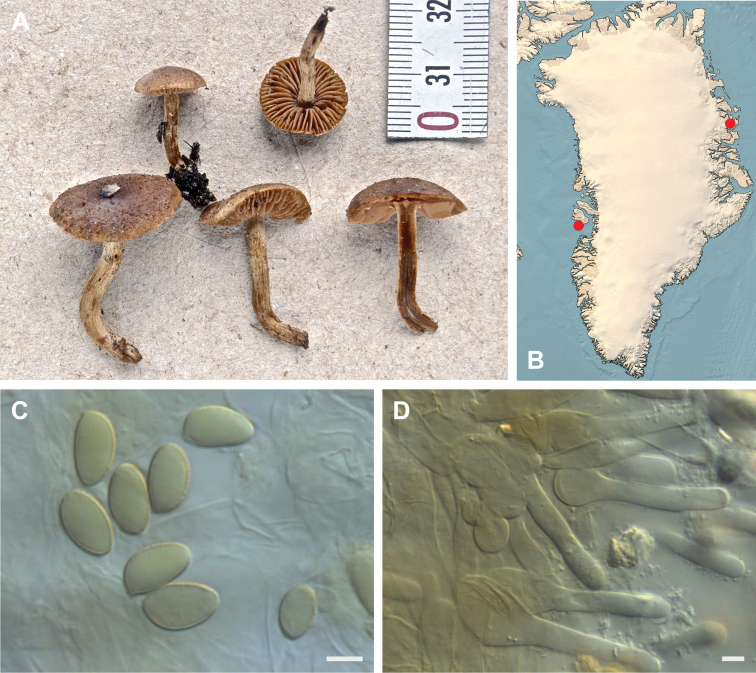
*Hebeloma pubescens***A** TB99.194, photograph T. Borgen **B** distribution of cited collections; **C** spores ×1600 and **D** cheilocystidia ×1000 of TB99.194 in Melzer’s reagent. Scale bars: 5 µm; microphotographs H.K.J. Beker.

#### Microscopic description.

Spores ellipsoid, sometimes ovoid, pale yellow to yellow brown, guttulate, sometimes weakly, on ave. 10.0–12.0 × 6.0–6.5 µm, ave. Q = 1.5–1.8, almost smooth or weakly ornamented (O1O2), perispore not loosening (P0), indextrinoid (D0). Basidia 28–33(-37) × 8–9 µm, ave. Q = 3.1–4.4, mostly four-spored. Cheilocystidia lageniform or ventricose, occasionally with characteristic basal or median wall thickening, septate, on ave. 38–45 × 4.5–6 (apex) × 4.5–6 (middle) × 8–10.5 (base) µm, ratios A/M = 1.02–1.10, A/B = 0.51–0.73, B/M = 1.52–2.10. Epicutis an ixocutis, 90–100 µm thick (measured from exsiccata), maximum hyphae width 6 µm, sometimes encrusted, trama elements beneath subcutis isodiametric up to 15 µm wide. Caulocystidia similar to cheilocystidia but less ventricose, up to 120 µm long.

#### Collections examined.

**W-Greenland**: Disko, Kangaarsuk, Fortune Bay, 69.25°N, 53.54°W, 3 Aug 1986, T. Borgen (TB86.128, C-F-5089), 0 m, with *Salix* sp. **N-Greenland**: Zackenberg, W of Kærelv, 74.5°N, 21°W, 13 Aug 1999, T. Borgen (TB99.305, C-F-119753), 50 m, with *Dryas* sp. in scrubland. Zackenberg, W of Zackenberg River, 74.5°N, 21°W, 5 Aug 1999, T. Borgen (TB99.194, C-F-119750), 50 m, with *Dryas* sp. and *Salix arctica* in scrubland.

#### Distribution.

Recently described from three records on Svalbard (Beker et al. 2016). The Greenland records are all north of 69° and, together with the type from 78°N, establish this as one of the few agarics, which appear only to occur in the High Arctic zone. The Greenland records are new to North America and the first outside Europe. High Arctic.

#### Habitat and ecology.

Three collections, two with *Dryas* and one with *Salix* sp. The collections from Zackenberg were on rich soil. Beker et al. (2016) described it from Svalbard, where the host was *Salix polaris*.

### Hebeloma spetsbergense

Taxon classificationFungiAgaricalesHymenogastraceae

Beker & U. Eberh.; Beker, Eberhardt & Vesterholt, Fungi Europ. (Alassio) 14: 180, 2016.

E94FE2D8-2B80-5ED3-B8F7-0BEAC3A3621E

Fig. 20

#### Macroscopic description.

Cap 1.0–2.2 cm in diameter, convex to umbonate, sometimes umbilicate, margin smooth, tacky when moist, occasionally hygrophanous, unicolored, sometimes bicolored, at center sepia to fuscous or dark brick, at margin pinkish buff to clay pink to cinnamon, sometimes quite thin margin, without remains of universal veil, partial veil present. Lamellae initially light clay, at maturity slightly more brownish, attachment emarginate, maximum depth 3–5 mm, number of lamellae {L} 18–35, droplets absent, white fimbriate edge sometimes present but weak. Stem (1.5–)1.9–3.1(–3.7) × 0.2–0.3(–0.5) cm, light watery brownish, darker towards base, cylindrical, stem Q (5–)8–12.7(–16.5), fibrillose, usually pruinose at apex. Context firm, stem interior stuffed, later hollow, stem flesh variably discoloring from base. Smell raphanoid sometimes strongly. Taste weakly raphanoid. Spore deposit sepia.

**Figure 20.**
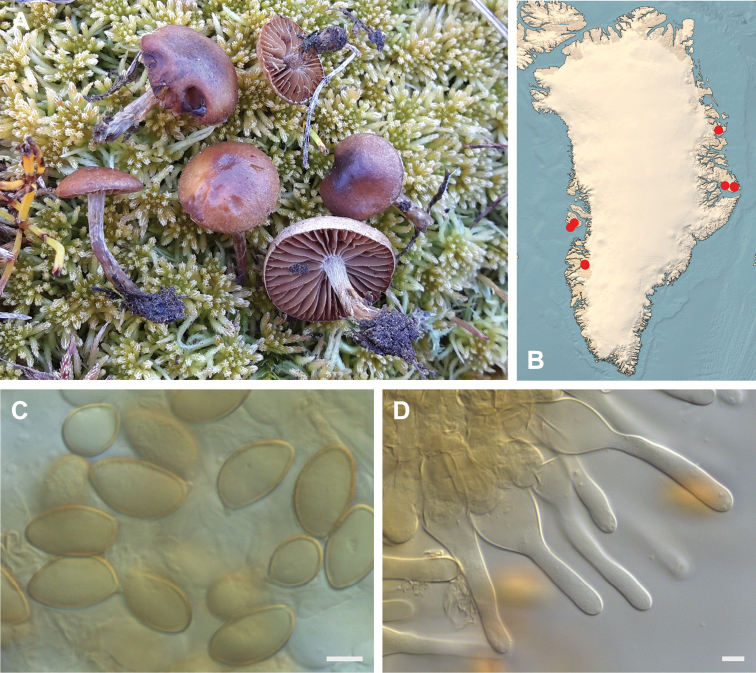
*Hebeloma spetsbergense***A** HK16.071, photograph H. Knudsen **B** distribution of cited collections **C** spores ×1600 and **D** cheilocystidia ×1000 of HK16.071 in Melzer’s reagent. Scale bars: 5 µm; microphotographs H.K.J. Beker.

#### Microscopic description.

Spores amygdaloid, occasionally limoniform, guttulation variable, on ave. 12.0–14.5 × 7.5–8.5 µm, ave. Q = 1.5–1.7, yellow brown to brown, almost smooth to weakly ornamented (O1O2), perispore not loosening (P0), rather strongly dextrinoid (D2D3). Basidia 27–37(-40) × 9–12 µm, ave. Q = 3.3–3.9, mostly four-spored. Cheilocystidia lageniform, ventricose, occasionally with characteristically median wall thickening, some septate, on ave. 45–60 × 4.5–6 (apex) × 4.5–5 (middle) × 8.5–12 (base) µm, occasionally cystidia with yellow contents, ratios A/M = 0.92–1.25, A/B = 0.43–0.61, B/M = 1.87–2.61. Epicutis an ixocutis, 30–35 µm thick (measured from exsiccata), maximum hyphae width 5 µm, without encrustations, shape of trama elements beneath subcutis sausage-shaped, up to 18 µm wide. Caulocystidia similar to cheilocystidia but generally larger.

#### Collections examined.

**W-Greenland**: Disko, Fortune Bay, 69.31°N, 53.88°W, 3 Aug 1986, T. Borgen (TB86.121, C-F-103478), 20 m, with *Salix glauca* in dunes. Qeqertarsuaq/Disko, Godhavn, 69.65°N, 53.32°W, 5 Aug 1986, S.A. Elborne (SAE-86.135-GR, C-F-119733), 0 m, with *Salix glauca* at lakeside. Kangerlussuaq, just west of Lake Ferguson, 66.99°N, 50.61°W, 6 Aug 2016, T. Borgen (TB16.056G, C-F-103579), 50 m, with *Salix glauca* and *Betula nana* in ditch. Kangerlussuaq, Kløftsøerne, 67°N, 50.71°W, 20 Aug 2016, S.A. Elborne (SAE-2016.102-GR, C-F-106740), 270 m, with *Betula nana* and *Bistorta vivipara* along lakeside. Kangerlussuaq, Kløftsøerne, 67.03°N, 50.69°W, 19 Aug 2016, H. Knudsen (HK s.n., C-F-108450), 500 m, with *Salix* sp. and *Betula* sp. Kangerlussuaq, NE facing slopes along Lake Ferguson, 66.99°N, 50.61°W, 12 Aug 2016, H. Knudsen (HK16.071, C-F-104100), 300 m. **N-Greenland**: Zackenberg, just N of Zackenberg Hut, 74.5°N, 21°W, 9 Aug 1999, T. Borgen (TB99.256, C-F-119757), 50 m, with *Salix arctica*, *Bistorta vivipara* and *Sphagnum* in scrubland. **E-Greenland**: Jameson Land, Constable Pynt, Draba Sibirica Elv, 10 km from coast, 71.03°N, 24.23°W, 23 Jul 1983, D. Boertmann (DB GR 83-62, C-F-119801), 50 m, with *Salix arctica* along riverside. Jameson Land, Nerlerit Inaat/Constable Pynt, delta of Gåseelv valley, 70.76°N, 22.65°W, 12 Aug 2017, T. Borgen (TB17C.113, C-F-106781), 40 m, with *Bistorta vivipara* in heathland. Jameson Land, Nerlerit Inaat/Constable Pynt, delta of Gåseelv valley, 70.76°N, 22.65°W, 9 Aug 2017, S.A. Elborne (SAE-2017.174-GR, C-F-106764), 40 m, with *Salix arctica* along riverside. Jameson Land, Nerlerit Inaat/Constable Pynt, Hareelv, 70.7°N, 22.68°W, 2 Aug 2017, S.A. Elborne (SAE-2017.043-GR, C-F-106760), 200 m, with *Salix arctica* in bog. Jameson Land, Nerlerit Inaat/Constable Pynt, near airstrip, 70.74°N, 22.64°W, 6 Aug 2017, T. Borgen (TB17C.050, C-F-106774), 50 m, with *Salix* sp. in fen.

#### Distribution.

Recently described from high arctic Svalbard (Beker et al. 2016). The eleven Greenland records indicate that it is quite common in the High Arctic zone in Greenland north of 67°N. Circumpolar, High Arctic.

#### Habitat and ecology.

Twelve collections, four recorded with *Salix arctica*, four with *S. glauca*, two with *Salix* spp., one with *Betula nana* and one with *Bistorta vivipara*. *Bistorta* is often present near collections of mycorrhizal fungi, but only when it was the only possibility was this host recorded. In the Rocky Mountains, Cripps et al. (2019) reported two collections growing with *S. glauca* and *S. arctica*. In Greenland, *H. spetsbergense* is growing both in acid and calcareous habitats, most often in humid localities like bogs, riversides and ditches. The five collections from Svalbard, including the type, are from localities on base-poor ground with *S. polaris* (Beker et al. 2016).

##### *Hebeloma* sect. Denudata (Fr.) Sacc.; Fl. Ital. Crypt. I 15: 691, 1916.

Veil absent. Cap uni- or bicolored. Lamellae often exuding small hyaline or colored drops. Cheilocystidia long, swollen in the apical part, constricted in the median part, basal part swollen or not. Spores amygdaloid to limoniform, ornamented, sometimes strongly.

###### *Hebeloma* subsect. *Crustuliniformia Quadr.*; Doc. mycol. 14: 30, 1985 (“1984”).

Cheilocystidia distinctly broadened at apex, base ± cylindrical, wall not thickened. Lamellae edge often with exuded drops.

### Hebeloma alpinum

Taxon classificationFungiAgaricalesHymenogastraceae

(J. Favre) Bruchet; Bull. mens. Soc. linn. Lyon 39(6(Suppl.)): 68, 1970.

F36078B2-E621-5B45-A107-5CAF27463BC0

Fig. 21

#### Macroscopic description.

Cap 1.7–7.3 cm, convex to umbonate or broadly umbonate; margin smooth, often involute, sometimes crenulate or serrate, tacky when moist, not hygrophanous, almost unicolored, occasionally bicolored, at center cream to pink buff to dark olive buff brown or yellowish brown to brownish olive or umber to sepia, clay buff or cinnamon, margin cream to pink buff or clay buff, sometimes very thin, universal veil absent, partial veil absent. Lamellae whitish, later light gray brown, then sordid brownish, number of lamellae {L} 40–72, emarginate, 0.3–0.9 cm broad, exuded drops usually visible with naked eye, but sometimes absent, with white fimbriate edge. Stem 1.5–5.0 × 0.4–1.2 cm, whitish fimbriate-pruinose in the entire length, later downwards slightly sordid ochraceous, ochre-yellowish or pale brown, base cylindric to clavate, rarely bulbous, occasionally tapering, pruinose to floccose particularly at apex, when fresh with droplets. Trama firm, stem with a stuffed interior, occasionally becoming hollow with age, not discoloring. Smell usually raphanoid, occasionally of cocoa or absent. Taste mild to weakly bitter. Spore deposit dark olive buff, brownish olive to clay buff.

**Figure 21.**
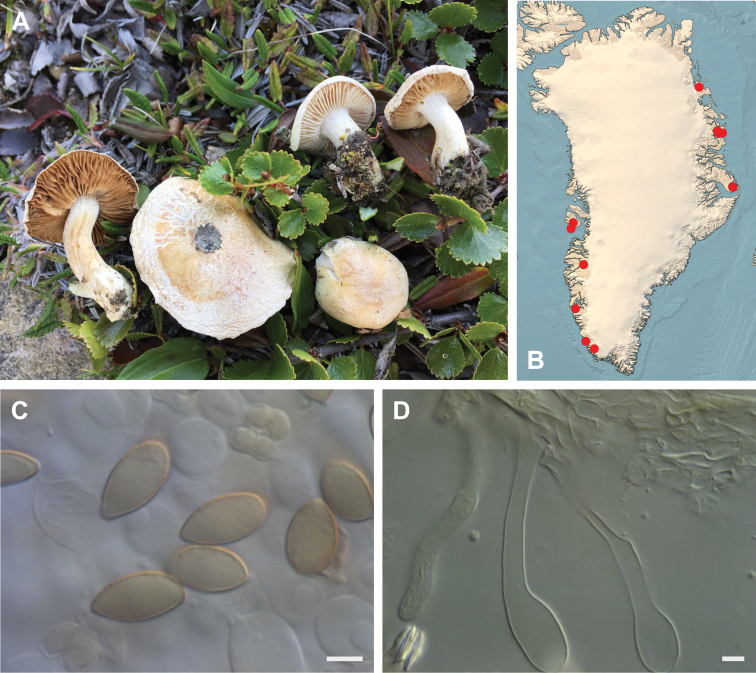
*Hebeloma alpinum***A** SAE-2017.006, photograph S.A. Elborne **B** distribution of cited collections **C** spores ×1600 and **D** cheilocystidia ×1000 of SAE-2017.006 in Melzer’s reagent. Scale bars: 5 µm; microphotographs H.K.J. Beker.

#### Microscopic description.

Spores amygdaloid to limoniform, on ave. 9.5–15 × 5.5–8.5 µm, ave. Q = 1.4–2.2, yellow brown to brown, sometimes guttulate, papillate, sometimes very strongly, almost smooth to weakly ornamented (O1O2 (03)), perispore not or somewhat loosening (P0P1), indextrinoid to weakly dextrinoid (D0D1D2). Basidia 28–40(–45) × 8–12 µm, Q = 3–4.3, mostly four-spored. Cheilocystidia mainly clavate-stipitate or spathulate-stipitate, occasionally slenderly clavate or clavate-lageniform, occasional apically thickening, rostrate, septate or sinuate, on ave. 40–71 × 7–10 (apex) × 3.5–5 (middle) × 3.5–6 (base) µm, ratios A/M = 1.6–2.7, A/B = 1.5–2.7, B/M = 0.9–1.2. Pileipellis an ixocutis, 60–160 µm thick (measured from exsiccata), hyphae 5–6 µm broad, some encrusted; trama elements beneath subcutis cylindrical, ellipsoid, thick sausage-shaped, up to 18 µm wide. Caulocystidia similar to cheilocystidia, up to 120 µm long and 11 µm wide.

#### Collections examined.

**S-Greenland**: Paamiut, 62.01°N, 49.4°W, 18 Aug 1984, T. Borgen (TB84.148, C-F-103537), 25 m, with *Salix herbacea* and *Salix glauca*. Sermiliarsuk, Sioralik, Aasivii, 65.53°N, 48.33°W, 30 Aug 1997, T. Borgen (TB97.153a, C-F-103507), 50 m, with *Betula pubescens* and *Salix glauca* in copse. Sermiliarsuk, Sioralik, Aasivii, 65.53°N, 48.33°W, 30 Aug 1997, T. Borgen (TB97.152, C-F-103506), 50 m, with *Dryas integrifolia* in heathland. **W-Greenland**: Disko, Qeqertarsuaq, 69.24°N, 53.54°W, 5 Aug 1986, T. Borgen (TB86.141, C-F-103565), 0 m. Disko, Skarvefjeld at Qeqertarsuaq, 69.65°N, 53.32°W, 2 Aug 1986, T. Borgen (TB86.115, C-F-103458), 400 m, with *Dryas integrifolia*. Kangerlussuaq, airport area, 67.02°N, 50.69°W, 1 Aug 1995, T. Borgen (TB95.004, C-F-103503), 50 m, with *Salix glauca* and *Betula nana* in copse. Kangerlussuaq, Hassells Fjeld, Kløftsøerne, 67.01°N, 50.71°W, 28 Aug 2018, H. Knudsen (HK18.390D, C-F-111119), 50 m, with *Salix glauca* in tundra. Kangerlussuaq, near hotel, 67.02°N, 50.7°W, 7 Aug 1986, T. Borgen (TB86.153, C-F-101230), 50 m, with *Salix glauca* and *Betula nana* in tundra. Kobbefjord, NuukBasic, 64.08°N, 51.23°W, 24 Aug 2018, H. Knudsen (HK18.308, C-F-111115), 5 m, in tundra. **N-Greenland**: Daneborg, 0.5 km E of Airstrip, 74.2°N, 20.1°W, 29 Jul 2006, T. Borgen (TB06.034, C-F-119763), 20 m, with *Dryas* sp. in heathland. V. Clausen Fjord, 77.52°N, 20.66°W, 13 Aug 1990, B. Fredskild (BF 90 loc. 6, C-F-5180), 0 m. Zackenberg, Aucellabjerg, 74.47°N, 21°W, 9 Aug 2006, T. Borgen (TB06.137, C-F-119766), 160 m, with *Dryas* sp. and *Salix arctica* in heathland. Zackenberg, Aucellabjerg, 74.47°N, 21°W, 11 Aug 1999, T. Borgen (TB99.283, C-F-119808), 100 m, with *Dryas* sp. in heathland. Zackenberg, few 100 m west of Zackenberg River, 74.47°N, 21°W, 3 Aug 1999, T. Borgen (TB99.199, C-F-104294), 50 m, with *Dryas* sp. in scrubland. Zackenberg, just S of Kamelen, 74.47°N, 21°W, 27 Jul 1999, T. Borgen (TB99.115, C-F-119806), 50 m, with *Dryas* sp. and *Salix arctica* at riverside. Zackenberg, just S of Teltdammen, 74.47°N, 21°W, 20 Jul 1999, T. Borgen (TB99.027, C-F-119742), 40 m, with *Dryas* sp. in scrubland. Zackenberg, just S of Teltdammen, 74.3°N, 21°W, 20 Jul 1999, T. Borgen (TB99.023, C-F-119744), 40 m, with *Dryas* sp. and *Salix* sp. in scrubland. Zackenberg, just W of Kærelv, 74.47°N, 21°W, 30 Jul 1999, T. Borgen (TB99.159, C-F-119807), 30 m, with *Salix arctica* and *Bistorta vivipara* on solifluction lobe. **E-Greenland**: Jameson Land, Nerlerit Inaat/Constable Pynt, 70.74°N, 22.65°W, 31 Jul 2017, S.A. Elborne (SAE-2017.008-GR, C-F-106758), 10 m, with *Dryas* sp. and *Salix* sp. in tundra. Jameson Land, Nerlerit Inaat/Constable Pynt, 70.74°N, 22.67°W, 31 Jul 2017, S.A. Elborne (SAE-2017.006-GR, C-F-106757), 60 m, with *Betula nana* and *Dryas* sp. in tundra. Jameson Land, Nerlerit Inaat/Constable Pynt, around the airport, 70.74°N, 22.64°W, 31 Jul 2017, H. Knudsen (HK17.001, C-F-104889), 50 m. Jameson Land, Nerlerit Inaat/Constable Pynt, around the airport, 70.74°N, 22.64°W, 31 Jul 2017, H. Knudsen (HK17.005, C-F-104893), 50 m. Jameson Land, Nerlerit Inaat/Constable Pynt, around the airport, 70.74°N, 22.64°W, 31 Jul 2017, H. Knudsen (HK17.006, C-F-104894), 50 m. Jameson Land, Nerlerit Inaat/Constable Pynt, around the airport, 70.74°N, 22.64°W, 31 Jul 2017, H. Knudsen (HK17.007, C-F-104895), 50 m. Jameson Land, Nerlerit Inaat/Constable Pynt, delta of Gåseelv valley, 70.76°N, 22.65°W, 8 Aug 2017, T. Borgen (TB17C.089, C-F-106779) ,40 m, with *Bistorta vivipara* in fenland. Jameson Land, Nerlerit Inaat/Constable Pynt, delta of Gåseelv valley, 70.76°N, 22.65°W, 6 Aug 2017, H. Knudsen (HK17.123, C-F-105024), 40 m. Jameson Land, Nerlerit Inaat/Constable Pynt, delta of Gåseelv valley, 70.76°N, 22.65°W, 7 Aug 2017, H. Knudsen (HK17.148, C-F-105050), 40 m. Jameson Land, Nerlerit Inaat/Constable Pynt, Gåseelv, Harris Fjeld, 70.75°N, 22.68°W, 3 Aug 2017, H. Knudsen (HK17.062, C-F-104951), 95 m. Jameson Land, Nerlerit Inaat/Constable Pynt, Hareelv, 70.7°N, 22.68°W, 2 Aug 2017, H. Knudsen (HK17.049, C-F-104938), 200 m. Jameson Land, Nerlerit Inaat/Constable Pynt, Hareelv, 70.7°N, 22.68°W, 2 Aug 2017, H. Knudsen (HK17.054, C-F-104943), 200 m. Jameson Land, Nerlerit Inaat/Constable Pynt, Hareelv, 70.7°N, 22.68°W, 10 Aug 2017, S.A. Elborne (SAE-2017.188-GR, C-F-106766), 100 m, with *Salix arctica* in bog. Jameson Land, Nerlerit Inaat/Constable Pynt, Mt. Harris, 70.75°N, 22.68°W, 6 Aug 2017, T. Borgen (TB17C.053, C-F-106775), 100 m, with *Dryas* sp. and *Bistorta vivipara* in heathland. Jameson Land, Nerlerit Inaat/Constable Pynt, north of Primulaelv, 70.75°N, 22.66°W, 1 Aug 2017, S.A. Elborne (SAE-2017.014-GR, C-F-106759), 10 m, with *Dryas* sp. Jameson Land, Nerlerit Inaat/Constable Pynt, Primulaelv, 70.74°N, 22.67°W, 13 Aug 2017, T. Borgen (TB17C.134, C-F-106784), 180 m, with *Bistorta vivipara*. Jameson Land, Nerlerit Inaat/Constable Pynt, Primulaelv, 70.74°N, 22.67°W, 13 Aug 2017, H. Knudsen (HK17.278, C-F-105185), 180 m. Jameson Land, Nerlerit Inaat/Constable Pynt, Primulaelv, 70.74°N, 22.67°W, 1 Aug 2017, H. Knudsen (HK17.023, C-F-104912), 180 m, in tundra.

#### Distribution.

*Hebeloma alpinum* is one of the five most often recorded *Hebeloma* species in Greenland, with 36 records (almost 10%) of those considered here. This is in good accordance with Beker et al. (2016), who pointed out that this is “the most common *Hebeloma* species we have collected in arctic/alpine areas”. It occurs all over Greenland, from Sermiliarsuk in the south (61.6°N) to Zackenberg (74.5°N) and V. Clausen Fjord (77.5°N) in the north. In Constable Pynt in East Greenland in 2017 it was the most common of all mushrooms. In Europe, it is known from the major mountain ranges (the Alps, the Pyrenees, the Tatras, the Carpathians, Black Mountains in Montenegro), from supraboreal Iceland (Beker et al. 2016). It has been reported in North America from the Rocky Mountains (Montana, Cripps et al. 2019). Arctic-alpine, boreal and subarctic.

#### Habitat and ecology.

The 36 collections are all from calcareous localities. The preferred host is *Dryas integrifolia* but possibly also *D. octopetala*. When these two occur together as in NE Greenland, they often hybridize, and can be difficult to identify. Only a few collections are reported with other hosts like *Salix arctica*, *S. herbacea*, *Betula pubescens*, *B. nana* and even *Bistorta vivipara*. The only record from Rocky Mountains was associated with *S. arctica* and *S. glauca*. Beker et al. 2016 also have records with *S. polaris*, *S. retusa* and *S. reticulata*.

#### Notes.

The shape of the cheilocystidia clearly points to subsect. Crustuliniformia. Within this subsection, it is recognized among the other species by its habitat in arctic-alpine localities, the number of lamellae being usually between 40 and 60 and the size of the spores, on average > 11 μm long and > 6 μm wide. The squat and robust stature is a good character in the field.

*Hebeloma bellotianum* Berk. is a closely related or synonymous species from Bellot Island in the Canadian Arctic (Eberhardt et al. 2015b). Unfortunately, attempts to sequence the type have been unsuccessful. It has spores on ave. 14.7 × 7.1 µm whereas the largest average measurements of 72 collections of *H. alpinum* is 13.7 × 7.7 µm.

### Hebeloma arcticum

Taxon classificationFungiAgaricalesHymenogastraceae

Beker & U. Eberh. sp. nov.

7E504E7B-A54A-5C5F-A870-A13BF970B161

838874

Figs 22 , 23

#### Type.

Greenland. Sisimiut: at the bridge on the road to the airport (approx. 66.944317N, 53.670444W, alt. approx. 0 m a.s.l.), 14 Aug. 2016, H. Knudsen (HK16.119) (***Holotype***: C-F-104149; Database Record: HJB17673; Genbank: ITS = MW445587, *Tef1*a = MW452584 and *RPB2* = MW452593).

**Figure 22.**
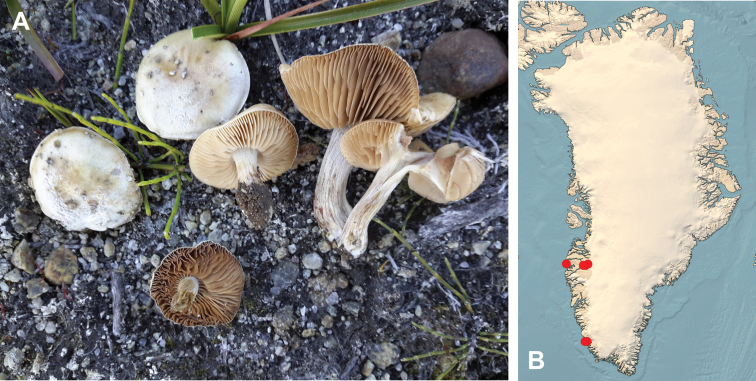
*Hebeloma arcticum***A** HK16.119 (holotype), photograph H. Knudsen **B** distribution of cited collections.

#### Diagnosis.

Favoring arctic type habitats with many cheilocystidia clavate-stipitate and rather strongly dextrinoid but indistinctly ornamented spores.

#### Etymology.

*arcticus* (adj. Latin), meaning the arctic, to emphasize the habitat within which this mushroom has been discovered.

#### Macroscopic description.

Cap (1.7–) 2.4–2.9 (–3.8) cm, plano-convex, with or without suggestion of obtuse umbo, with decurved margin then applanate, only weakly viscid even after heavy rainfall, dull, almost glabrous, marginal zone whitish pale due to a fine adpressed tomentum, often eroded, usually bicolored, center ochre, paler towards the margin becoming very pale brownish, hardly hygrophanous but a few hygrophanous dots. Lamellae emarginate with tooth, ventricose, number of lamellae {L} 36–44, up to 4 mm wide, initially pale, darker (clay buff) at maturity with whitish fimbriate edge and usually some watery droplets or droplets visible with a x10 lens. Stem (2.8–) 4.1–4.4 (–6.0) × 0.5–0.7 (–0.9) cm, to 0.6–0.9 cm at base, whitish fibrillose to pruinose, cylindrical, narrowly fistulose, downwards pale yellowish to pale brownish when rubbed. Context firm, thick particularly in center, whitish pale, watery when moist, in age slightly brownish downwards in stipe. Smell weakly herbaceous-raphanoid. Taste mild. Spore print at most clay buff (Munsell 10YR6/4; in TB 90.071).

**Figure 23.**
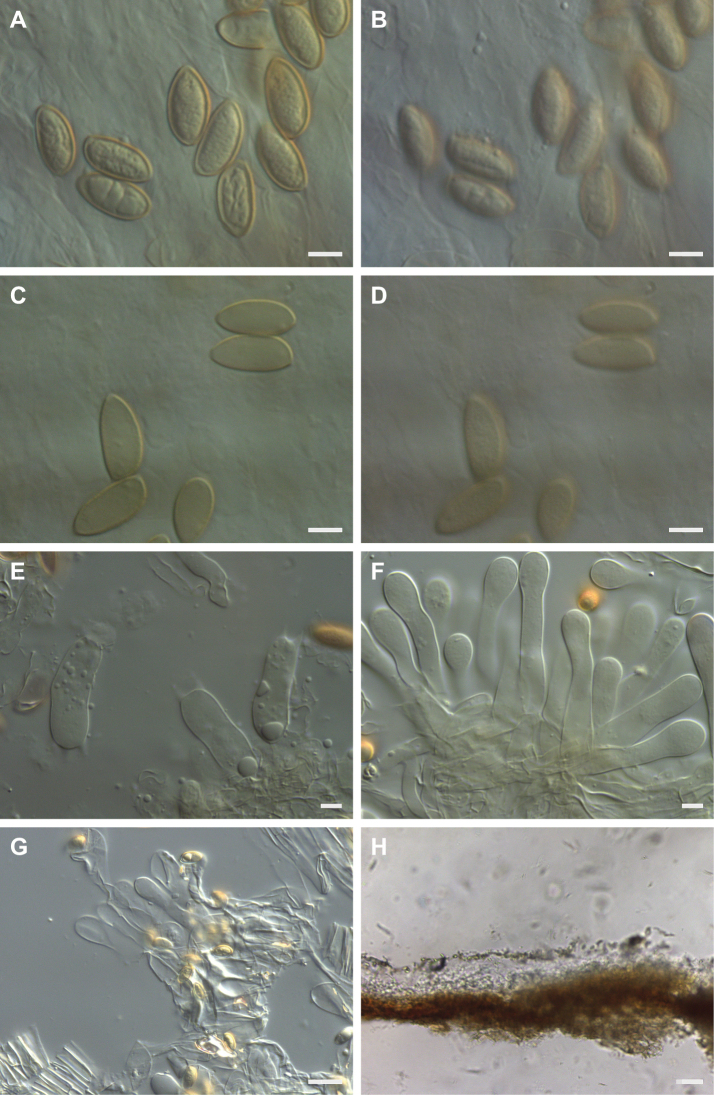
*Hebeloma arcticum* HK16.119 (holotype) **A** spores ×1600 and **B** spore ornamentation ×1600 in Melzer’s reagent **C** spores and **D** spore ornamentation ×1600 in KOH **E** basidia cheilocystidia and **F** cheilocystidia ×1000 in Melzer’s reagent **G** caulocystidia ×500 in KOH **H** cutis ×125 in KOH. Scale bars: 5 µm (**A–F**); 10 µm (**G**); 50 µm (**H**); microphotographs H.K.J. Beker.

#### Microscopic description.

Spores amygdaloid, ellipsoid or ovoid, on ave. (across eight collections) 10.8–12.0 × 6.4–6.9 µm, ave. Q = 1.65–1.83, (for the holotype, measuring 102 spores, 5% to 95% percentile range 10.4–13.2 × 5.9–7.2 µm with median 11.9 × 6.5 µm and ave. 11.9 × 6.5 µm and S.D. for length 0.84 µm and for width 0.39 µm, and Q value 5% to 95% percentile range 1.57–2.04, with median 1.83 and ave. 1.83 with S. D. 0.14), yellow brown, guttulate, not papillate, almost smooth (O1), perispore not loosening (P0), distinctly to strongly dextrinoid (D2D3). Basidia (20) 22–37 × 6–10 μm; ave. Q 3.0–3.7, (for the holotype 25–37 × 7–9, ave. Q 3.6), mostly four-spored. Cheilocystidia mainly clavate-stipitate, sometimes clavate-ventricose, more rarely capitate-stipitate, occasional apical or median wall thickening, sometimes septate (occasionally clamped), on ave. 36–57 × 6–9 (apex) × 4–5 (middle) × 4–6.5 (base) µm, ratios A/M = 1.5–2.3, A/B = 1.2–2.0, B/M = 1.0–1.4, (for the holotype, width near apex, 5% to 95% percentile range 6.5–8.3 µm, with median 7.4 µm and ave. 7.4 µm with S.D. 0.63 µm and over all 45 × 7.4 (apex) × 5 (middle) × 6.5 (base) µm). Pileipellis an ixocutis, up to 90 µm thick (measured from exsiccata), hyphae up to 6 µm broad, none encrusted; trama elements beneath subcutis isodiametric to ellipsoid or thick sausage-shaped, up to 17.5 µm wide. Caulocystidia similar to cheilocystidia, up to 90 µm long.

#### Other collections examined.

**S-Greenland**: Paamiut, 62.01°N, 49.4°W, 31 Aug 1986, T. Borgen (TB86.277A, C-F-103571), 10 m, with *S. arctophila*, 62.01°N, 49.4°W, 4 Sep 1990, T. Borgen (TB90.083, C-F-103555), 10 m, with *Salix glauca* and *Bistorta vivipara* in fenland. Paamiut, 62.01°N, 49.4°W, 1 Sep 1990, T. Borgen (TB90.071, C-F-103483), 10 m, with *Salix glauca* and *Bistorta vivipara*. Paamiut, near cemetery, 61.9941°N, 49.6666°W, 21 Aug 2008, T. Borgen (TB08.153, C-F-106751), 15 m, with *Salix glauca*. **W-Greenland**: Kangerlussuaq, Ringsødalen, Ringsøen, 66.9853°N, 50.9464°W, 9 Aug 2016, T. Borgen (TB16.095, C-F-103584), 180 m, with *Salix glauca*. Kangerlussuaq, Ringsødalen, Ringsøen, 66.9853°N, 50.9464°W, 9 Aug 2016, H. Knudsen (HK16.044, C-F-104080), 180 m, with *Salix glauca*. Kangerlussuaq, Russels Glacier, 67.0977°N, 50.2318°W, 12 Aug 2000, S.A. Elborne (SAE-2000.021-GR, C-F-108472), 220 m, with *Salix glauca*.

#### Distribution.

This is a new species known from a number of records in the Low Arctic zone in southern and western Greenland. The fact that it was collected several times independently may indicate that it is quite common in these areas.

#### Habitat and ecology.

Eight collections, seven with *Salix glauca* one with *S. arctophila*. Most collections are from mineral-poor ground.

#### Notes.

The mainly clavate-stipitate cheilocystidia indicate that this species belongs in H. sect. Denudata and there in H. subsect. Crustuliniformia.Few species of the subsection have almost smooth spores or spores that are rather strongly dextrinoid. The combination of almost smooth and rather strongly dextrinoid spores is unique in this subsection. Molecular results support its placement within this subsection. Within H. sect. Denudata, based on the spore characters, *H. arcticum* is readily identifiable. To be noted is also the unusual shape of the spores; while many spores are amygdaloid, many others appear more ellipsoid or cylindrical, again uncommon for this section of *Hebeloma*. As Figs 5A-B show, *H. arcticum* is well distinct from related taxa and is in a supported clade (Fig. 5B) with other members of H. subsect. Crustuliniformia, but is not a member of the *H. alpinum*-complex. The clade of the species is supported by 100% bootstrap in the analysis of three loci (Fig. 5B), but based on current knowledge, ITS alone is sufficient to recognize the taxon; in the ITS tree (not shown) calculated prior to concatenation of the data, *H. arcticum* received 84% bootstrap support.

### Hebeloma aurantioumbrinum

Taxon classificationFungiAgaricalesHymenogastraceae

Beker, Vesterh. & U. Eberh.; Eberhardt, Beker & Vesterholt, Persoonia 35: 116, 2015.

E8BC8183-2DC2-596A-A34E-A5BACF682393

Fig. 24

#### Macroscopic description.

Cap 0.8–3.6 cm in diameter, umbonate or campanulate, sometimes slightly tomentose or adpressed subtomentose, at high magnification, margin smooth, tacky when moist, not generally hygrophanous, but often with hygrophanous spots, uniformly colored or variably bicolored, at center yellow brown to cinnamon to umber, but with orange elements, at margin pinkish buff to clay buff, usually very thin, without traces of any veil. Lamellae initially whitish then becoming darker and brown with maturity, emarginate, maximum depth 2 mm, number of lamellae {L} 26–42, droplets present and visible, white fimbriate edge present. Stem 1.0–4.0 × 0.15–0.5 cm, whitish lengthily fibrillose on whitish-pale ground, then sordid buff or yellowish, cylindrical, occasionally clavate, stem Q (4.7–)5.5–7, pruinose, particularly at apex. Context firm, stem interior stuffed, flesh not discoloring from base. Smell raphanoid, sometimes weakly. Taste mild or slightly bitter. Spore deposit clay buff.

**Figure 24.**
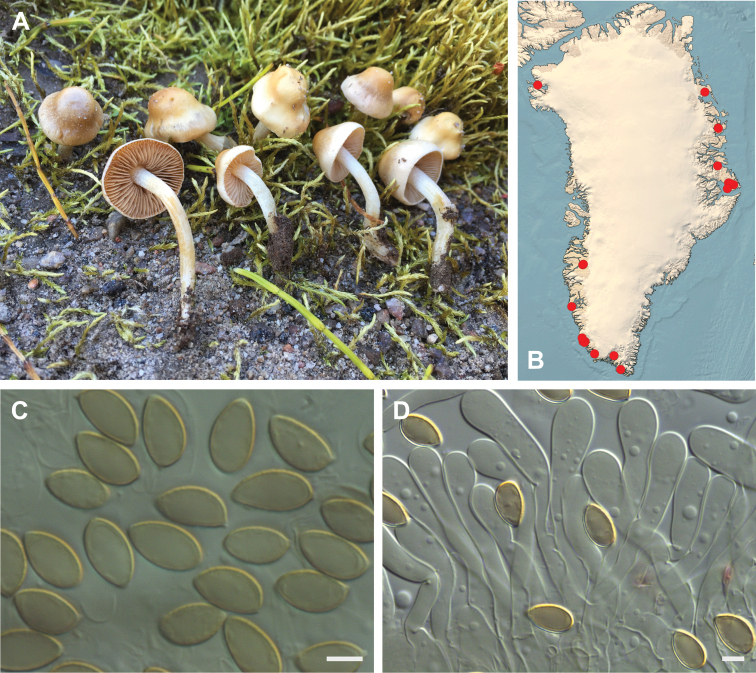
*Hebeloma aurantioumbrinum***A** SAE-2016.146, photograph S.A. Elborne **B** distribution of cited collections **C** spores ×1600 and **D** cheilocystidia ×1000 of SAE-2016.146 in Melzer’s reagent. Scale bars: 5 µm; microphotographs H.K.J. Beker.

#### Microscopic description.

Spores amygdaloid, not or weakly papillate, on ave. 10.0–12.0 × 6.0–7.0 µm, ave. Q = 1.6–1.9, pale brown to yellow-brown to brown, guttulate, almost smooth to weakly ornamented (O1O2 (O3)), perispore not or somewhat loosening (P0P1), indistinctly to weakly dextrinoid (D1D2). Basidia 28–38(-43) × 7–9(–11) µm, ave. Q = 3.2–4.4, mostly four-spored. Cheilocystidia clavate-stipitate, occasionally spathulate-stipitate or clavate-lageniform, occasionally characteristically with a median wall thickening, geniculate or septate, on ave. 45–61 × 7–8.5 (apex) × 4–5 (middle) × 4.5–5.5 (base) µm, with yellow contents, ratios A/M = 1.61–1.95, A/B = 1.62–1.98, B/M = 0.97–1.18. Epicutis an ixocutis, up to 70 µm thick (measured from exsiccata), maximum hyphae width 6 µm, sometimes encrusted, trama elements beneath subcutis ellipsoid up to 6 µm wide. Caulocystidia similar to cheilocystidia, up to 75 µm long.

#### Collections examined.

**S-Greenland**: Kangilineq/Kvaneøen, 61.95°N, 49.47°W, 25 Aug 1990, T. Borgen (TB90.057, C-F-103482), 40 m. Kangilinnguit, bottom of Laksebund, 61.25°N, 48.08°W, 21 Aug 2018, H. Knudsen (HK18.296, C-F-111114), 100 m, in tundra. North of Tasiusaq, across the fjord from Narsarsuaq, 61.17°N, 45.61°W, 31 Jul 1993, E. Rald (ER 93.091, C-F-104321), 150 m, in *Sphagnum* at the lakeside. Nugarssuk, 60.26°N, 44.76°W, 9 Aug 1993, E. Rald (ER 93.262, C-F-104315), 0 m. Paamiut, 62.01°N, 49.4°W, 23 Aug 1985, T. Borgen (TB85.239, C-F-103459), 90 m, with *Salix herbacea* and *Salix arctophila* in bog. Paamiut, 62.01°N, 49.4°W, 5 Aug 1993, T. Borgen (TB93.070, C-F-103502), 10 m. Paamiut, 62.01°N, 49.4°W, 1 Sep 1990, T. Borgen (TB90.072, C-F-103484), 10 m, with *Salix glauca* and *Bistorta vivipara*. Paamiut, 62.01°N, 49.4°W, 17 Aug 1990, T. Borgen (TB90.041, C-F-103481), 10 m, with *Salix glauca*. Paamiut, 62.01°N, 49.4°W, 19 Aug 1985, T. Borgen (TB85.217, C-F-103526), 10 m, with *Salix herbacea* in snowbed. Paamiut, 62.01°N, 49.4°W, 31 Aug 1986, T. Borgen (TB86.277B, C-F-137117), 10 m, with *Salix* sp. Paamiut, 62.01°N, 49.4°W, 19 Aug 1984, T. Borgen (TB84.150, C-F-119792), 15 m, with *Salix arctophila* and *Bistorta vivipara* in fenland. Paamiut, 62.01°N, 49.4°W, 15 Aug 1984, T. Borgen (TB84.132, C-F-103461), 15 m, with *Salix herbacea* in snowbed. Paamiut, 62.01°N, 49.67°W, 12 Aug 1984, T. Borgen (TB84.112, C-F-119737), 20 m, with *Salix* sp. Paamiut, 62.01°N, 49.4°W, 16 Aug 1984, T. Borgen (TB84.135, C-F-103462), 10 m, with *Salix arctophila*, *Bistorta vivipara* in fenland. Paamiut, 62°N, 49.67°W, 26 Jul 1985, D. Boertmann (DB 85-17, C-F-119784), 25 m, with *Salix* sp. in bog. Paamiut, 62°N, 49.67°W, 26 Jul 1985, D. Boertmann (DB 85-28, C-F-119785), 25 m, with *Salix* sp. in bog. Paamiut, Avigaat, 62.23°N, 49.83°W, 22 Sep 1990, T. Borgen (TB90.133, C-F-103485), 60 m, with *Salix herbacea* and *Bistorta vivipara*. Paamiut, Navigation School area, 62.01°N, 49.4°W, 17 Aug 1990, T. Borgen (TB90.039, C-F-103546), 10 m, with *Salix herbacea* and *Bistorta vivipara*. Paamiut, Navigation School area, 62.01°N, 49.4°W, 13 Aug 1990, T. Borgen (TB90.029, C-F-103545), 10 m, with *Salix herbacea*. Paamiut, Navigation School area, 62.01°N, 49.4°W, 12 Aug 1990, T. Borgen (TB90.012, C-F-103547), 10 m, with *Salix herbacea*. Paamiut, Navigation School area, 62.01°N, 49.4°W, 12 Aug 1990, T. Borgen (TB90.011, C-F-103548), 10 m, with *Salix herbacea*. Paamiut, Telesletten, 62.01°N, 49.4°W, 23 Aug 1986, T. Borgen (TB86.251, C-F-103570), 10 m. **W-Greenland**: Kangerlussuaq, Lake Ferguson, Tasersuatsiaq, 66.96°N, 50.70°W, 22 Aug 2016, S.A. Elborne (SAE-2016.146-GR, C-F-106745), 350 m, with *Salix glauca* in streambed. Kangerlussuaq, NE facing slopes along Lake Ferguson, 66.99°N, 50.61°W, 12 Aug 2016, H. Knudsen (HK16.089, C-F-104118), 300 m. Nuuk, 64.19°N, 51.67°W, 14 Aug 1987, S.A. Elborne (SAE-1987.113-GR, C-F-119789), 300 m, with *Salix herbacea* in snowbed. Nuuk, airport area, 64.19°N, 51.67°W, 5 Aug 1987, H. Knudsen (HK87.004, C-F-119790), 100 m. Nuuk, Lille Malene, 64.19°N, 51.67°W, 17 Aug 1987, H. Knudsen (HK87.218, C-F-119788), 100 m. **N-Greenland**: Qaanaaq, 77.47°N, 69.18°W, 8 Aug 1988, S.A. Elborne (SAE-1988.149-GR, C-F-1461), 50 m. Danmarkshavn, Mørkefjord Station, 76.93°N, 20.32°W, 7 Aug 1984, B. Lauritsen (BL s.n., C-F-3637), 10 m. Danmarkshavn, Mørkefjord Station, 76.93°N, 20.32°W, 26 Jul 1982, B. Lauritsen (BL s. n., C-F-119797), 10 m. Zackenberg, Gadekæret, 74.5°N, 21°W, 21 Jul 1999, T. Borgen (TB99.044, C-F-119751), 40 m, with *Salix arctica* and *Bistorta vivipara* in scrubland. Zackenberg, Teltdammen, 74.5°N, 21°W, 5 Aug 2006, T. Borgen (TB06.091, C-F-119741), 30 m, with *Salix arctica* in scrubland. Zackenberg, Teltdammen, 74.5°N, 21°W, 5 Aug 2006, T. Borgen (TB06.091, C-F-119781), 30 m, with *Salix arctica* in scrubland. Zackenberg, Ulvehøj, 74.47°N, 21°W, 11 Aug 2006, T. Borgen (TB06.150, C-F-119768), 40 m, with *Salix arctica* in snowbed. Zackenberg, Østkæret, 74.47°N, 21°W, 22 Aug 2006, T. Borgen (TB06.259, C-F-119771), 40 m, with *Salix arctica* in fenland. **E-Greenland**: Jameson Land, Constable Pynt, beginning of Lollandselv, 70.92°N, 23.18°W, 31 Jul 1989, S.A. Elborne (SAE-89.430, C-F-2424), 500 m, with *Salix arctica* on riverside. Jameson Land, Constable Pynt, camp at ‘Vindelv’, river wnw of pt.330, 71°N, 23.47°W, 30 Jul 1989, H. Knudsen (HK89.366, C-F-2309), 230 m, with *Salix arctica*. Jameson Land, Constable Pynt, delta of Jyllandselv, 70.68°N, 24.06°W, 28 Jul 1988, D. Boertmann (DB GR 88-22, C-F-119787), 0 m. Jameson Land, Constable Pynt, Gåseelv, 70.77°N, 22.7°W, 18 Jul 1989, S.A. Elborne (SAE-89.121, C-F-2327), 80 m, with *Salix arctica* on lakeside. Lower east Skeldal, Kong Oscars Fjord, 72.27°N, 24.28°W, 17 Aug 1962, T.T. Elkington (s.n., C-F-6992), 150 m, with *Cassiope* in tundra and heathland. Lower east Skeldal, Kong Oscars Fjord, 72.27°N, 24.28°W, 17 Aug 1962, T.T. Elkington (s.n., C-F-6993), 150 m, with *Cassiope*, tundra and heathland.

#### Distribution.

*Hebeloma aurantioumbrinum* is one of the five most commonly recorded *Hebeloma* in Greenland with 10.5% of the records considered here. It is common and widely distributed in arctic areas in Europe (Beker et al. 2016). From alpine areas, it is known from North America (Cripps et al. 2019), but not from the well-investigated alpine mountains in Europe (Beker et al. 2016). Circumpolar, arctic-alpine.

#### Habitat and ecology.

Forty collections with a number of hosts recorded, including: *Salix herbacea* (11), *S. arctica* (5), *S. glauca* (5), *S. arctophila* (3), *Salix* sp. (5), *Bistorta vivipara* (8, but never as the only possible associate). The localities are also scattered between wet and dry places, but favoring wet areas with *S. herbacea*, without having a preference for specific soil conditions. Beker et al. (2016) also found a preference for *Salix*, including *S. polaris*.

### Hebeloma geminatum

Taxon classificationFungiAgaricalesHymenogastraceae

Beker, Vesterh. & U. Eberh.; Eberhardt, Beker & Vesterholt, Persoonia 35: 122, 2015.

1CC309F6-A81A-5D5C-AAA6-0E06DE3D285A

Fig. 25

#### Macroscopic description.

Cap 2.0–12.0 cm in diameter, usually convex, sometimes broadly umbonate or applanate, margin usually smooth, occasionally involute, crenulate or upturned with age, tacky when moist, not hygrophanous usually uniformly colored, rarely bicolored, at center usually cream or whitish, sometimes a little yellowish, at margin paler, cream to whitish, without any traces of veil. Lamellae initially very pale brown, later clay-brown, adnate to emarginate, maximum depth 4–8 mm, number of lamellae {L} 62–100, with visible droplets and white fimbriate edge. Stem 1.5–11.0 × 0.7–1.5 {median} × 0.7–1.6 {base} cm, initially whitish, later pale brownish, initially pruinose, apex with watery droplets, cylindrical to clavate, stem Q (2.1–)2.5–9.2(–10.6), floccose. Context firm, stem interior stuffed, becoming hollow with age, flesh usually not discoloring from base. Smell raphanoid. Taste mild, raphanoid. Spore deposit brownish olive to grayish brown.

**Figure 25.**
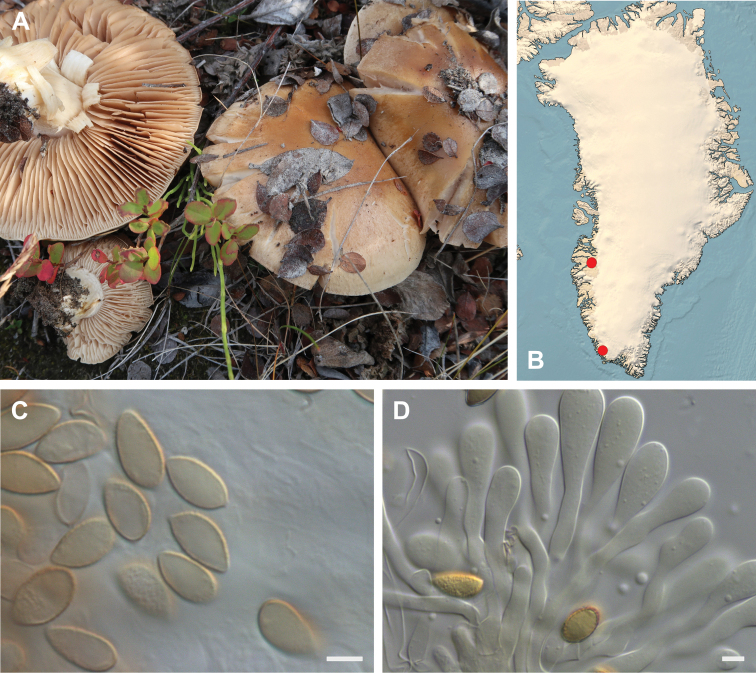
*Hebeloma geminatum***A** HK18.379B, photograph H. Knudsen **B** distribution of cited collections **C** spores ×1600 and **D** cheilocystidia ×1000 of HK18.379B in Melzer’s reagent. Scale bars: 5 µm; microphotographs H.K.J. Beker.

#### Microscopic description.

Spores amygdaloid, on ave. 10.0–11.0 × 5.5–6.5 µm, ave. Q = 1.7–1.9, grayish yellow to yellow-brown to brown, guttulate, weakly to distinctly ornamented (O2O3), perispore somewhat loosening ((P0) P1 (P2)), indextrinoid or indistinctly dextrinoid (D0D1). Basidia 25–35 × 7–9 µm, ave. Q = 3.3–4.3, mostly four-spored. Cheilocystidia clavate-stipitate or spathulate-stipitate and occasionally capitate-stipitate or clavate-lageniform, occasionally characteristically bifurcate, with a median wall thickening, septate or sinuate, on ave. 50–72 × 8–10.5 (apex) × 4–4.5 (middle) × 3.5–5 (base) µm, ratios A/M = 1.76–2.57, A/B = 1.68–2.85, B/M = 0.81–1.19. Epicutis an ixocutis, 110–200 µm thick (measured from exsiccata), maximum hyphae width 5–8 µm, variably encrusted, trama elements beneath subcutis cylindrical, ellipsoid, sausage-shaped up to 16 µm wide. Caulocystidia similar to cheilocystidia, up to 70 µm long and 12 µm wide.

#### Collections examined.

**S-Greenland**: Sermiliarsuk, Sioralik, Aasivii, 65.53°N, 48.33°W, 30 Aug 1997, T. Borgen (TB97.154a, C-F-103508), with *Betula pubescens*, 50 m. **W-Greenland**: Kangerlussuaq, c. 2 km W of the Airport, Mt. Hassel, 67.06°N, 50.68°W, 10 Aug 2000, A-M. Larsen, T. Borgen (TB00.065, C-F-103514), 50 m, with *Salix glauca* in copse. Kangerlussuaq, Hassells Fjeld, Kløftsøerne, 67.01°N, 50.71°W, 27 Aug 2018, H. Knudsen (HK18.379B, C-F-111117), 300 m, with *Salix glauca*, *Betula nana* in heathland.

#### Distribution.

Only known from two localities in southwestern Greenland. Generally distributed over much of Europe, but lacking in the Mediterranean region. Northernmost European localities are from Norway at just above 70°N. This species has already been recorded in North America (https://mycoportal.org/portal/collections/list.php, accessed 2 Dec 2020) but how common it is, is not yet known. Temperate and boreal, with a few records from the Low Arctic zone.

#### Habitat and ecology.

Only three collections with *Salix glauca*, *Betula nana* and *B. pubescens*, but when recorded on 27 Aug. 2018, on the south-exposed side of Mt. Hassell, it was numerous and, remarkably, the only agaric present. From Europe, there are a number of hosts recorded, including many deciduous and coniferous trees (Beker et al. 2016).

### Hebeloma helodes

Taxon classificationFungiAgaricalesHymenogastraceae

J. Favre; Beitr. Kryptfl. Schweiz 10(3): 214, 1948.

8B5AB0F0-D57F-573C-B2A9-7B15834F3D24

Fig. 26

#### Macroscopic description.

Cap 1.3–3.8 cm in diameter, convex or sometimes weakly umbonate becoming umbilicate with age, margin usually involute at least when young, tacky when moist, not hygrophanous, mostly uniformly colored or variably bicolored, at center light ochraceous to yellowish to yellowish-brown or pale reddish brown and at margin whitish to pale cream, sometimes with remains of universal veil. Lamellae initially whitish later persistently cream, attachment emarginate, occasionally adnate, maximum depth 3–4.5 mm, number of lamellae {L} 33–54, droplets present and visible at least with × 10 lens, white fimbriate edge present. Stem 1.5–6.5 × 2.0–7.0 {median} × (2–)2.9–6.5(–7) {base} cm, whitish tomentose flocculose in the entire length, cylindrical to clavate, stem Q (3.3–)4.7–20, floccose. Context firm, stem interior stuffed, later becoming hollow, stem flesh not discoloring from base but with some weak discoloration with age. Smell raphanoid. Taste mild, raphanoid. Spore deposit dark olive buff to brownish olive.

**Figure 26.**
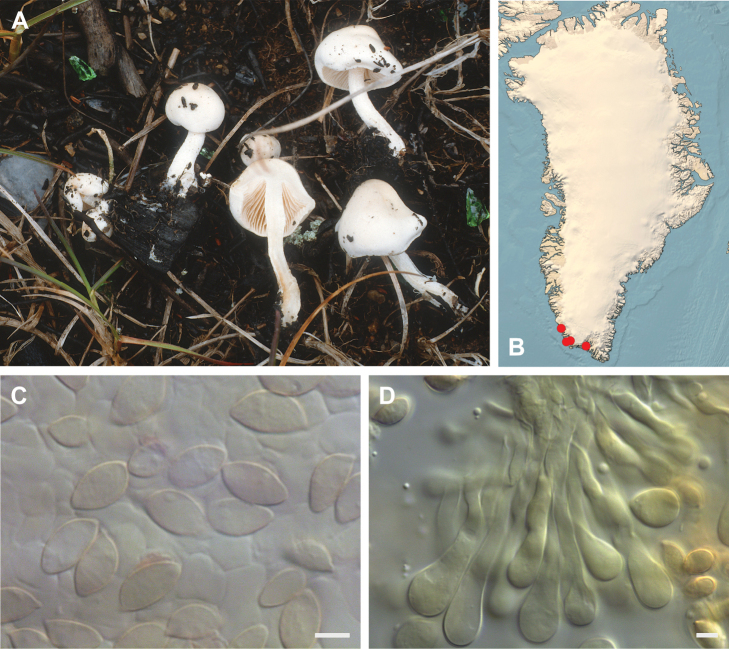
*Hebeloma helodes***A** TB88.114, photograph T. Borgen **B** distribution of cited collections **C** spores ×1600 and **D** cheilocystidia ×1000 of TB88.114 in Melzer’s reagent. Scale bars: 5 µm; microphotographs H.K.J. Beker.

#### Microscopic description.

Spores amygdaloid, on ave. 9.0–11.0 × 5.0–6.0 µm, ave. Q = 1.6–2.0, yellow to pale brown, usually guttulate, weakly to distinctly ornamented (O2O3), perispore somewhat to distinctly loosening ((P0) P1P2), indextrinoid to indistinctly dextrinoid, rarely distinctly dextrinoid (D0D1 (D2)). Basidia 22–27(–30) × 7–9 µm, ave. Q = 2.8–4.2, mostly four-spored. Cheilocystidia clavate-stipitate to capitate stipitate, occasionally more clavate-lageniform, often with characteristic apical or less frequently median wall thickening, occasionally septate, on ave. 44–63 × 8.5–11.5 (apex) × 4–5 (middle) × 3.5–5.5 (base) µm, ratios A/M = 1.90–2.86, A/B = 2.02–3.38, B/M = 0.77–1.17. Epicutis an ixocutis, 100–135 µm thick (measured from exsiccata), maximum hyphae width 5–6 µm, sometimes encrusted, trama elements beneath subcutis sausage-shaped up to 15 µm wide. Caulocystidia similar to cheilocystidia, but short, up to 11 µm wide at apex.

#### Collections examined.

**S-Greenland**: Kangilinnguit, 61.14°N, 48.6°W, 10 Aug 1985, T. Borgen (TB85.072, C-F-103460), 25 m, with *Alnus alnobetulae* in copse. Kangilinnguit, 61.14°N, 48.6°W, 11 Aug 1985, T. Borgen (TB85.090, C-F-103476), 25 m, with *Alnus alnobetulae* in copse. Kangilinnguit, 61.23°N, 48.10°W, 10 Aug 1985, T. Borgen (TB85.065, C-F-103525), 25 m, with *Salix glauca* in heathland. Narsaq, 60.91°N, 46.05°W, 3 Aug 1993, E. Rald (ER 93.153, C-F-104317), 20 m, with *Salix glauca* in scrubland. Paamiut, 62.01°N, 49.4°W, 29 Aug 1988, T. Borgen (TB88.114, C-F-4003), 50 m, in scrubland.

#### Distribution.

Only found in a few localities in southern Greenland. Generally distributed in warm and cold temperate Europe with a few records from subarctic Norway and Finland and missing in southern Europe. The Greenland records fit well with the European records, the records from southern Greenland (Paamiut, 62.01) being the northernmost hitherto found.

#### Habitat and ecology.

Five collections from heath- and scrubland recorded with *Salix glauca* and *Alnus alnobetulae*. Beker et al. (2016) suspect association with various deciduous tree families.

### Hebeloma louiseae

Taxon classificationFungiAgaricalesHymenogastraceae

Beker, Vesterh. & U. Eberh.; Eberhardt, Beker & Vesterholt, Persoonia 35: 127, 2015.

AE6CF12A-791A-52E4-97B2-E89F47BA3A53

Fig. 27

#### Macroscopic description.

Cap 0.7–2.2 cm in diameter, convex to broadly umbonate, margin smooth to crenulate, tacky when moist, not hygrophanous, uniformly colored or indistinctly bicolored, at center clay-buff to dark olive buff, margin very thin, grayish pink or central color extending to margin, with no traces of veil. Lamellae initially pale, later sordid clay-brown, emarginate, maximum depth 2 mm, number of lamellae {L} 28–40, droplets often absent but occasionally visible even with the naked eye, white fimbriate edge present. Stem 0.8–2.5 × 0.2–0.3 {median} × 0.1–0.3 {base} cm, initially pale, later sordid brownish, cylindrical to clavate, occasionally tapering, stem Q (4–)4.6–7.6(–9.2), pruinose to slightly fibrillose. Context firm, interior stuffed, flesh discoloring weakly from base. Smell absent or raphanoid. Taste not recorded. Spore deposit grayish brown.

**Figure 27.**
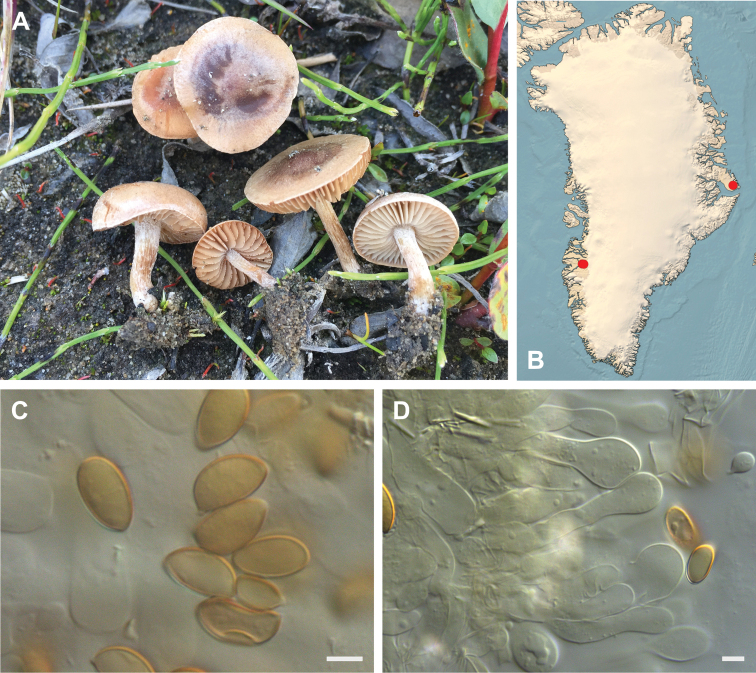
*Hebeloma louiseae***A** SAE-2017.125, photograph S.A. Elborne **B** distribution of cited collections **C** spores ×1600 and **D** cheilocystidia ×1000 of HK17.128A.146 in Melzer’s reagent. Scale bars: 5 µm; microphotographs H.K.J. Beker.

#### Microscopic description.

Spores amygdaloid, limoniform, weakly to strongly papillate, on ave. 12.0–13.0 × 7.0–7.5 µm, ave. Q = 1.6–1.8, yellow brown to brown, guttulate, almost smooth to weakly ornamented (O1O2), perispore not loosening (P0), indextrinoid to indistinctly dextrinoid (D0D1 (D2)). Basidia 24–56 × 6–12 µm, ave. Q = 3.2–4.6, mostly four-spored. Cheilocystidia clavate-stipitate, occasionally spathulate-stipitate or capitate-stipitate or clavate-lageniform, occasionally with characteristic apical wall thickening, occasionally bifurcate, septate or sinuate, on ave. 42–59 × 6–11 (apex) × 4.0–5.5 (middle) × 3.5–6.0 (base) µm, ratios A/M = 1.48–2.51, A/B = 1.99–2.92, B/M = 0.81–1.24. Epicutis an ixocutis, up to 100 µm thick (measured from exsiccata), maximum hyphae width 6 µm, sometimes encrusted, trama elements beneath subcutis cylindrical, sausage-shaped. Caulocystidia similar to cheilocystidia, up to 75 µm long.

#### Collections examined.

**W-Greenland**: Kangerlussuaq, c. 2 km W of the Airport, 67.06°N, 50.68°W, 10 Aug 2000, A-M. Larsen, T. Borgen (TB00.061, C-F-103518), 50 m, with *Salix glauca* in ditch. Kangerlussuaq, Sandflugtsdalen, 67.06°N, 50.42°W, 25 Aug 2016, S.A. Elborne (SAE-2016.197-GR, C-F-106747), 110 m, with *Salix glauca* in dune slack. **E-Greenland**: Jameson Land, Nerlerit Inaat/Constable Pynt, delta of Gåseelv valley, 70.76°N, 22.65°W, 6 Aug 2017, H. Knudsen (HK17.128, C-F-105029), 40 m. Jameson Land, Nerlerit Inaat/Constable Pynt, delta of Gåseelv valley, 70.76°N, 22.65°W, 6 Aug 2017, S.A. Elborne (SAE-2017.125A-GR, C-F-106763), 40 m, with *Salix arctica* at riverside. Jameson Land, Nerlerit Inaat/Constable Pynt, delta of Gåseelv valley, 70.77°N, 22.69°W, 6 Aug 2017, S.A. Elborne (SAE-2017.125B-GR, C-F-106763), 15 m, with *Salix arctica* at riverside.

#### Distribution.

Recently described from Svalbard (Beker et al. 2016). The five Greenland collections all occurred north of 67°N and thus provide evidence that *H. louiseae* is a species of the High Arctic zone. It has not yet been recorded in alpine regions. The Greenland collections are the first from North America and the first outside Europe.

#### Habitat and ecology.

Five collections, with *Salix glauca* and *S. arctica* in calcareous soil and seemingly moist localities. From Svalbard it was recorded with *S. polaris* (Beker et al. 2016).

#### Notes.

*Hebeloma louiseae* is easily recognized among the arctic *Hebeloma* species by the small basidiomes, the relatively few lamellae and the large basidia, although not present in every collection.

### Hebeloma minus

Taxon classificationFungiAgaricalesHymenogastraceae

Bruchet; Bull. mens. Soc. linn. Lyon 39(6(Suppl.)): 126, 1970.

80F3B6E1-C218-5213-803B-B57C77C24EA4

Fig. 28

#### Macroscopic description.

Cap 0.9–3.1 cm in diameter, convex to umbonate, margin smooth, sometimes involute, tacky when moist, sometimes hygrophanous especially when frosted, almost uniformly colored, occasionally more bicolored, at center dark pinkish buff to dark olive buff or clay-buff to brownish olive, grayish brown or umber, at margin pinkish to grayish buff or clay-buff, occasionally pruinose (givré), without any traces of veil. Lamellae clay buff, emarginate to adnate, maximum depth 3.5–5 mm, number of lamellae {L} 30–34, droplets often visible, occasionally absent or visible with × 10 lens, white fimbriate edge present. Stem 1.0–4.0 × 0.1–0.8 {median} × 0.1–1.0 {base} cm, stem Q (4–)4.8–14(–20), fairly strongly whitish fibrillose-flocculose, cylindrical to clavate, rarely bulbous, fibrillose, pruinose, floccose, particularly noticeable at apex. Context firm, stem interior stuffed, hollow with age, flesh discoloring from base at most very weakly. Smell raphanoid. Taste mild, occasionally weakly bitter or raphanoid. Spore deposit brownish olive.

**Figure 28.**
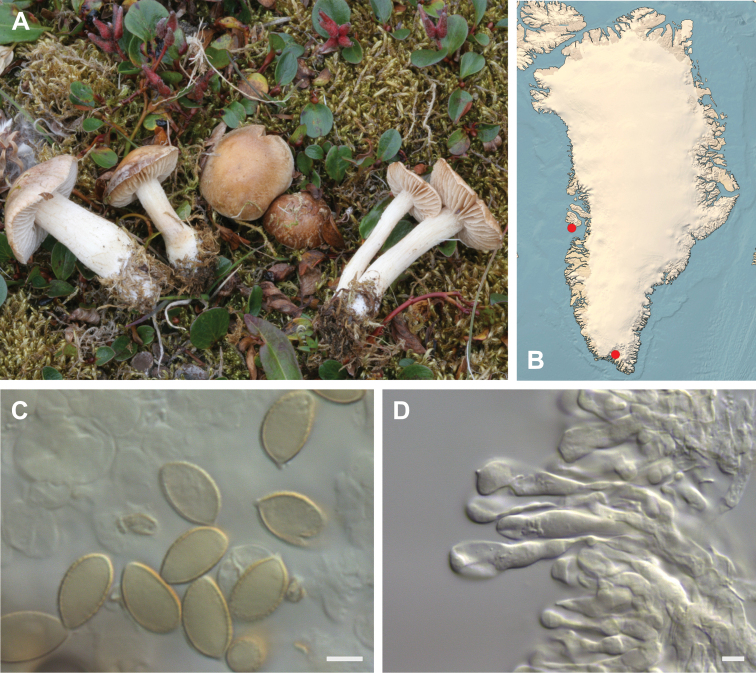
*Hebeloma minus***A** HJB11945 (from Svalbard), photo H.K.J. Beker, reproduced by kind permission from Beker et al. (2016)**B** distribution of cited collections **C** spores ×1600 and **D** cheilocystidia ×1000 of TB86.085 in Melzer’s reagent. Scale bars: 5 µm; microphotographs H.K.J. Beker.

#### Microscopic description.

Spores amygdaloid, occasionally limoniform, papillate, on ave. 11.0–13.0 × 6–7.5 µm, ave. Q = 1.6–1.9, yellow to brown, usually guttulate, weakly to distinctly ornamented (O2O3), perispore not or somewhat loosening (P0P1 (P2)), indistinctly to weakly dextrinoid (D1D2). Basidia 27–40 × 7–11 µm, ave. Q = 2.8–4.1, mostly four-spored. Cheilocystidia capitate-stipitate, clavate-stipitate or occasionally clavate-lageniform, occasionally with characteristic apical or median wall thickening, sometimes geniculate or septate, on ave. 40–60 × 8–11.0 (apex) × 3.5–5 (middle) × 3–6 (base) µm, ratios A/M = 1.87–2.67, A/B = 1.81–3.02, B/M = 0.84–1.24. Epicutis an ixocutis, 40–100 µm thick (measured from exsiccata), maximum hyphae width 6–6.5 µm, some encrusted, trama elements beneath subcutis cylindrical, sausage-shaped, up to 30 µm wide. Caulocystidia similar to cheilocystidia but larger, up to 100 µm long.

#### Collections examined.

**S-Greenland**: Qassiarsuk, Tasiusaq, 61.15°N, 45.52°W, 21 Jul 1984, T. Læssøe (TL 84.041, C-F-119793), 25 m. **W-Greenland**: Qeqertarsuaq/Disko, Godhavn, Østerlien, 69.25°N, 53.54°W, 30 Jul 1986, T. Borgen (TB86.085, C-F-104302), 40 m, with *Salix glauca* in copse.

#### Distribution.

Only two collections, from southwestern Greenland. Although described 50 years ago, *H. minus* is still only known from a few collections in Iceland and Svalbard, and from the French, Italian and Swiss Alps at 2200–2700 m. The Icelandic localities are from the upper boreal zone, but close to the oroboreal zone, which is equivalent to the alpine zone (Rivas-Martinez et al. 2004). The Svalbard localities are in the High Arctic, whereas the two Greenland collections are both from the Low Arctic zone. This is a truly arctic-alpine species. It is new to North America and these are the first records outside Europe.

#### Habitat and ecology.

Two collections, one recorded with *Salix glauca*. Beker et al. (2016) list *S. herbacea*, *S. reticulata*, *S. retusa* and *Dryas octopetala* as hosts from arctic and alpine sites in Europe.

##### Hebeloma subsect. Clepsydroida Beker & U. Eberh.; Fungal Biol. 120: 82, 2015 (“2016”).

Cheilocystidia distinctly broadened at apex, base ± swollen, wall often thickened at the middle. Lamellae edge often with exuded drops, but sometimes dried away.

### Hebeloma ingratum

Taxon classificationFungiAgaricalesHymenogastraceae

Bruchet; Bull. mens. Soc. linn. Lyon 39: 125, 1970.

B4C52857-150F-57CB-974D-95A327F098D2

Fig. 29

#### Macroscopic description.

Cap 1.8–7.5 cm in diameter, convex to umbonate, margin often involute when young, sometimes serrate or crenulate, tacky when moist, occasionally hygrophanous, usually bicolored but sometimes unicolored, at center usually brown tones from ochraceous to cinnamon, at margin cream, occasionally 3–4 mm from margin a circle of water spots can be visible, without any traces of veil. Lamellae pale clay brown, adnate to emarginate, maximum depth 3–5 mm, number of lamellae {L} 50–80, droplets absent or visible with × 10 lens, occasionally with naked eye, white fimbriate edge usually present, sometimes very strongly. Stem 1.5–6.8 × 0.4–1.2 {median} × 0.5–1.3 {base} cm, stem Q (2.5–)4.4–11.1(–11.5), white lengthily fibrillose and white flocculose, most distinct in the upper half, cylindrical to clavate, occasionally subbulbous to bulbous. Context firm, interior stuffed, later hollow, rarely with superior wick, flesh not discoloring from base or sometimes weakly. Smell raphanoid. Taste raphanoid, usually mild, but sometimes bitter. Spore deposit dark olive buff to brownish olive to yellowish brown or umber.

**Figure 29.**
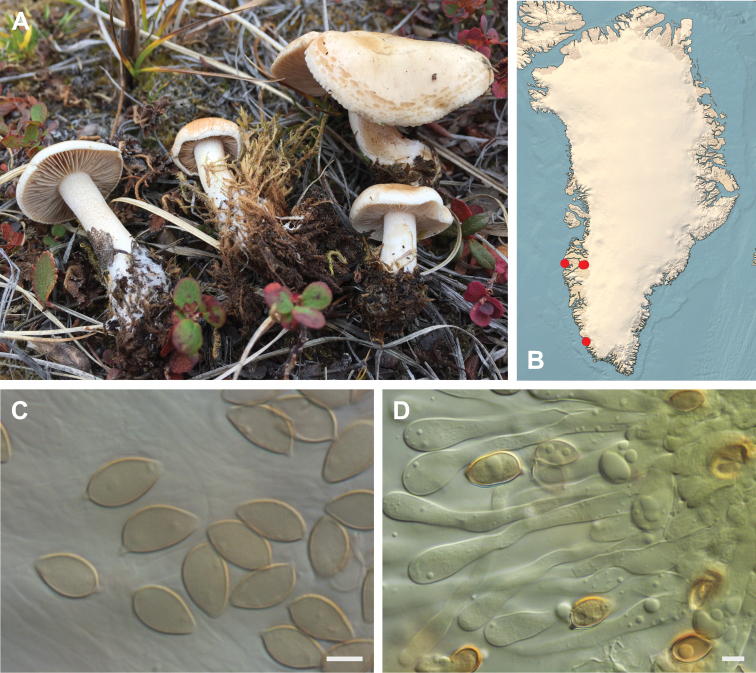
*Hebeloma ingratum***A** SAE-2016.208, photograph S.A. Elborne **B** distribution of cited collections **C** spores ×1600 and **D** cheilocystidia ×1000 of SAE-2016.208 in Melzer’s reagent. Scale bars: 5 µm; microphotographs H.K.J. Beker.

#### Microscopic description.

Spores amygdaloid, sometimes limoniform, variably papillate from absent to very strongly, on ave. 10.0–11.0 × 5.5–6.5 µm, ave. Q = 1.7–1.9, yellow to yellow brown, usually guttulate, weakly to distinctly ornamented (O2O3), perispore somewhat to distinctly loosening ((P0) P1P2), indistinctly to weakly dextrinoid (D1D2). Basidia (20–)24–30(–33) × 6–10 µm, ave. Q = 3.2–3.9, mostly four-spored. Cheilocystidia clavate-lageniform, occasionally clavate-stipitate or ventricose, occasionally with characteristic apical or median wall thickening, sometimes geniculate or septate, on ave. 42–55 × 5–7 (apex) × 3.5–4.5 (middle) × 5–6.5 (base) µm, ratios A/M = 1.4–1.74, A/B = 0.9–1.28, B/M = 1.4–1.64. Epicutis and ixocutis, 75–300 µm thick (measured from exsiccata), maximum hyphae width 5–6.5 µm, sometimes encrusted, trama elements beneath subcutis oblong to sausage-shaped up to 15 µm wide. Caulocystidia up to 150 µm long, often cylindrical or slenderly clavate at the apex.

#### Collections examined.

**S-Greenland**: Paamiut, Taartoq/Mørke Fiord, 62.01°N, 49.26°W, 4 Sep 1993, T. Borgen (TB93.205, C-F-103501), 20 m, with *Betula glandulosa* and *Salix glauca* in heathland. **W-Greenland**: Kangerlussuaq, fjord shore south of town, 66.98°N, 50.69°W, 26 Aug 2016, S.A. Elborne (SAE-2016.208-GR, C-F-106748), 20 m, with *Salix glauca* and *Betula nana* along streamside. Kangerlussuaq, Sandflugtsdalen, 67.02°N, 50.42°W, 21 Aug 1987, H. Knudsen (HK87.262, C-F-119732), 200 m, with *Betula nana* and *Salix glauca*. Sisimiut, 4 km E of the village, 66.94°N, 53.59°W, 18 Aug 2000, E. Ohenoja (EO18.8.00.36, OULU F050503), 70 m, in heathland.

#### Distribution.

Only known from two well-studied areas in low arctic southern and western Greenland, north to 67°. The general European distribution is in the Temperate zone, missing in southern Europe and with one record in the Hemiboreal zone in Finland. The Greenland records are the northernmost known and the first from North America and the first outside Europe.

#### Habitat and ecology.

Four collections from heath- and scrubland. Hosts are uncertain, both *Betula nana* and *B. glandulosa* as well as *Salix glauca* were present. No preference for a specific ecology. Beker et al. (2016) also list *Betula* and *Salix*, but also *Fagus*, *Quercus* and *Populus* as possible hosts.

### Hebeloma vaccinum

Taxon classificationFungiAgaricalesHymenogastraceae

Romagn.; Bull. trimest. Soc. mycol. Fr. 81: 333, 1965.

96B69855-37BA-5066-AFB6-91391BD7B8E3

Fig. 30

#### Macroscopic description.

Cap 0.9–6.1 cm in diameter, convex, later umbonate, margin usually involute when young, sometimes crenulate or scalloped, tacky when moist, not hygrophanous, usually bicolored, but sometimes almost unicolored, at center from clay-buff to yellowish brown and dark olive buff to dark brick or rust brown, at margin cream to gray-buff to clay-buff, pale and very thin, without any traces of veil. Lamellae light, then sordid gray-brown, adnexed to emarginate, maximum depth 2–6 mm, number of lamellae {L} 32–60, droplets visible with naked eye or sometimes with × 10 lens, white fimbriate edge present. Stem 0.9–5.5 × 0.3–1.3 {median} × 0.3–1.3 {base} cm, stem Q (2.2–)2.7–9(–13.3), white flocculose on pale brown ground, downwards watery gray-brown, later watery ochre-brown, not darker at base, base cylindrical to clavate or bulbous, sometimes with encrusted sand, pruinose to floccose, particularly at apex. Context firm, stem interior stuffed, later hollow, sometimes with superior wick, flesh not discoloring from base. Smell raphanoid, sometimes of cocoa. Taste sometimes raphanoid, sometimes bitter. Spore color brownish olive to sepia.

**Figure 30.**
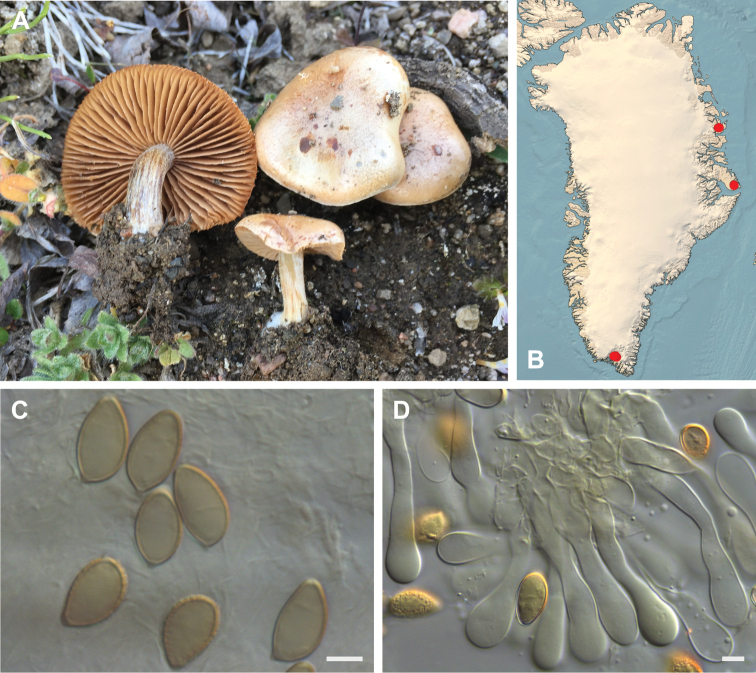
*Hebeloma vaccinum***A** SAE-2017.194, photograph S.A. Elborne **B** distribution of cited collections **C** spores ×1600 and **D** cheilocystidia ×1000 of SAE-2017.194 in Melzer’s reagent. Scale bars: 5 µm; microphotographs H.K.J. Beker.

#### Microscopic description.

Spores mainly amygdaloid, sometimes fusoid or limoniform, papillate, on ave. 12.0–14.5 × 6.5–8.0 µm, ave. Q = 1.6–2.0, yellow-brown to brown, sometimes guttulate, distinctly to fairly strongly ornamented ((O2) O3O4), perispore somewhat to distinctly loosening ((P0) P1P2), weakly to rather strongly dextrinoid (D2D3). Basidia 27–39 (–42) × 7–12 µm, ave. Q = 3–4.5, mostly four-spored. Cheilocystidia clavate-lageniform, capitate-lageniform, sometimes clavate-stipitate or capitate-stipitate or ventricose, occasionally characteristically with apical or median wall thickening, sometimes geniculate, septate or sinuate, on ave. 41–64 × 6–8 (apex) × 3–5 (middle) × 4.5–8 (base) µm, ratios A/M = 1.43–2.31, A/B = 0.84–1.53, B/M = 1.28–1.92. Epicutis and ixocutis, 40–125 µm thick (measured from exsiccata), maximum hyphae width 5–6.5 µm, sometimes encrusted, trama elements beneath subcutis sausage-shaped, occasionally spherical up to 16 µm wide. Caulocystidia similar to cheilocystidia, up to 90 µm long, often with many septa.

#### Collections examined.

**S-Greenland**: Narsarsuaq, 61.08°N, 45.26°W, 7 Aug 1985, T. Borgen (TB85.045, C-F-103477), 150 m, with *Dryas integrifolia* in heathland. Narsarsuaq, Hospitalsdalen, 61.17°N, 45.41°W, 1 Aug 1992, E. Rald (ER 92.038, C-F-104308), 60 m, with *Salix glauca* and *Betula glandulosa*. Narsarsuaq, 61.17°N, 45.41°W, 17 Aug 2015, H. Knudsen (HK15.069, C-F-8222), 60 m, with *Salix glauca* and *Betula pubescens* at pathside. Qassiarsuk, Tasiusaq, 61.15°N, 45.52°W, 19 Aug 1992, E. Rald (ER 92.323, C-F-104311), 25 m. Tasiusaq, 61.14°N, 45.63°W, 28 Jul 1993, E. Rald (ER 93.022, C-F-104320), 20 m, with *Salix glauca* in fenland. **N-Greenland**: Zackenberg, Aucellabjerg, 74.5°N, 21°W, 20. Aug. 2006, T. Borgen (TB06.246, C-F-119780), 300 m, with *Salix arctica* and *Bistorta vivipara* in scrubland. Zackenberg, just E of the station, 74.5°N, 21°W, 26 Jul 1999, T. Borgen (TB99.109, C-F-119755), 40 m, with *Dryas* sp. and *Salix* sp. in scrubland. Zackenberg, just S of the Field Station, 74.48°N, 20.76°W, 19 Jul 1999, T. Borgen (TB99.008, C-F-119804), 30 m, with *Dryas* sp. and *Bistorta vivipara*. **E-Greenland**: Jameson Land, Nerlerit Inaat/Constable Pynt, delta of Gåseelv valley, 70.76°N, 22.65°W, 8 Aug 2017, T. Borgen (TB17C.073, C-F-106778), 40 m, in heathland. Jameson Land, Nerlerit Inaat/Constable Pynt, Hareelv, 70.71°N, 22.69°W, 10 Aug 2017, S.A. Elborne (SAE-2017.194-GR, C-F-106767), 100 m, with *Salix arctica* in tundra. Jameson Land, Nerlerit Inaat/Constable Pynt, Primulaelv, 70.74°N, 22.67°W, 1 Aug 2017, H. Knudsen (HK17.022B, C-F-104911), 180 m. Jameson Land, Nerlerit Inaat/Constable Pynt, Primulaelv, 70.74°N, 22.67°W, 1 Aug 2017, H. Knudsen (HK17.021, C-F-104909), 180 m. Jameson Land, Nerlerit Inaat/Constable Pynt, Primulaelv, 70.74°N, 22.67°W, 1 Aug 2017, H. Knudsen (HK17.022A, C-F-104910), 180 m.

#### Distribution.

Widely distributed in Greenland and apparently common in the localities where it was found; missing in western Greenland. It is widely distributed in the Temperate zone in Europe (Beker et al. 2016) with a few records in subarctic areas in Sweden (Härjedalen) and the Oroboreal zone in Iceland (Egilsstadir, 65°N). The Greenland collections from Zackenberg (74.5°N) are the northernmost recorded, to date. *Hebeloma vaccinum* was recently recorded from the alpine Rocky Mountains (Cripps et al. 2019), but these Greenland records are the first from arctic North America.

#### Habitat and ecology.

Thirteen collections, all from calcareous or mineral rich areas. Hosts are *Dryas*, *Salix glauca* and *S. arctica*. Beker et al. (2016) concluded that the Salicaceae (*Salix* and *Populus*) are the host family for this species, with *S. repens* L. as the most often recorded host in Europe, but also *S. lanata* L.

##### *Hebeloma subsect. Hiemalia Quadr.*; Doc. mycol. 14: 30, 1985 (“1984”).

Cheilocystidia distinctly broadened at apex, base ± swollen, wall often thickened at the middle. Spores with a majority that are weakly ornamented (O2) and the perispore which is not consistently or distinctly loosening (rarely P2 and never P3) and the pileus color which always has brown or buff tones at least in the center.

### Hebeloma hiemale

Taxon classificationFungiAgaricalesHymenogastraceae

Bres.; Fung. trident. 2(11–13): 52, 1892.

42925A21-35B5-5EE1-8CD6-51C955081953

Fig. 31

#### Macroscopic description.

Cap 1.1–8.0 cm in diameter, convex to umbonate, often broadly, margin smooth, sometimes involute, undulate in older specimens, tacky when moist, occasionally spotted, rarely hygrophanous, almost uniformly colored or variably bicolored, at center warm buff to grayish buff or dark pinkish buff through honey or ochraceous and dark olive buff to umber and clay pink or orange brown, at margin pale cream to cream to honey or pale pinkish buff, without any traces of veil. Lamellae when young very pale but becoming darker with maturity, emarginate to adnate, maximum depth 3–7 mm, number of lamellae {L} 35–71, droplets usually visible with naked eye or × 10 lens, occasionally absent, white fimbriate edge present. Stem (1.3–)1.5–10.75(–12) × (0.3–)0.35–1.2 {median} × (0.3–)0.55–1.4(–1.5) {base} cm, stem Q 2.5–17.2(–17.4), whitish to pale brownish at middle, hardly discoloring, gradually sordid brown in the mid portion, cylindrical to clavate, rarely subbulbous, usually pruinose particularly at apex, rarely with mycelial chords. Context firm, stem interior stuffed, becoming hollow with age and often with a superior wick, flesh often discoloring from base, particularly after handling. Smell raphanoid, sometimes with hints of cocoa, rarely absent. Taste mild to weakly bitter. Spore color dark olive buff or grayish brown through brownish olive to umber.

**Figure 31.**
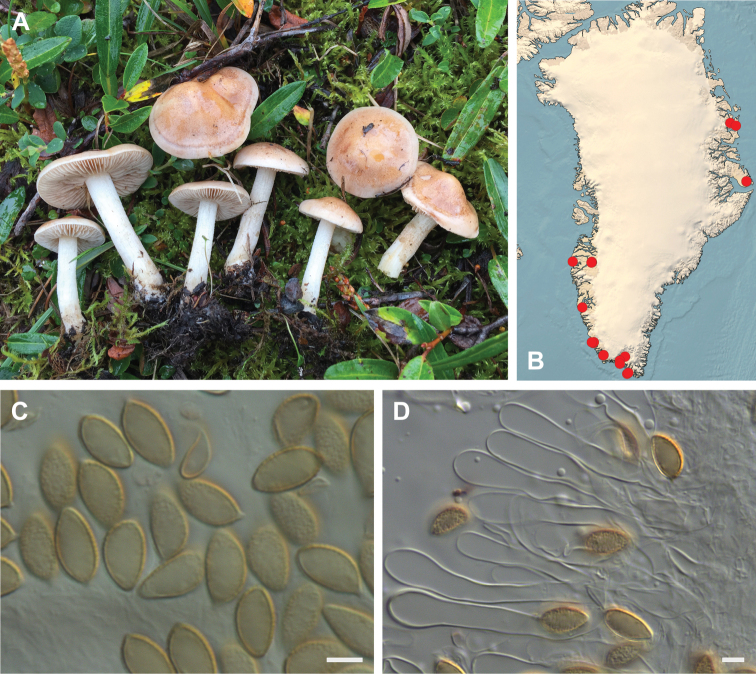
*Hebeloma hiemale***A** SAE-2018.225, photograph S.A. Elborne **B** distribution of cited collections **C** spores ×1600 and **D** cheilocystidia ×1000 of SAE-2018.225 in Melzer’s reagent. Scale bars: 5 µm; microphotographs H.K.J. Beker.

#### Microscopic description.

Spores amygdaloid, limoniform, variably papillate, on ave. 10.0–12.5 × 5.0–7.5 µm, ave. Q = 1.6–2.1, yellow to yellow brown, usually guttulate, weakly, occasionally distinctly ornamented ((O1) O2 (O3)), perispore not or somewhat loosening (P0P1 (P2)), indistinctly to weakly dextrinoid ((D0) D1D2). Basidia 23–39(-43) × 7–10 µm, ave. Q = 3.2–4.3, mostly four-spored, often stipitate. Cheilocystidia usually clavate-lageniform, but occasionally slenderly clavate, clavate-stipitate, cylindrical or ventricose, rarely spathulate-lageniform or tapering, occasionally characteristically with apical or median wall thickening, sometimes geniculate or septate, rarely sinuate or rostrate, on ave. 40–65 × 5.5–9 (apex) × 3.5–5 (middle) × 4.5–7.5 (base) µm, ratios A/M = 1.46–2.48, A/B = 0.97–1.8, B/M = 1.26–1.77. Epicutis an ixocutis, 60–200 µm thick (measured from exsiccata), maximum hyphae width 4–6 µm, sometimes encrusted, trama elements beneath subcutis cylindrical, ellipsoidal, sausage-shaped up to 17 µm wide. Caulocystidia similar to cheilocystidia, up to 75 µm long, often septate, septa sometimes clamped, many cylindrical.

#### Collections examined.

**S-Greenland**, Kangilinnguit, 61.21°N, 48.12°W, 20 Aug 2018, H. Knudsen (HK18.269, C-F-111112), 125 m, in tundra. Kangilinnguit, Arsuk Fjord, 61.23°N, 48.07°W, 19 Aug 2018, S.A. Elborne (SAE-2018.225-GR, C-F-112771), 100 m, with *Salix glauca* and *Bistorta vivipara* in heathland. Kangilinnguit, at Grønnedal Hut, 61.23°N, 48.08°W, 15 Aug 1985, T. Borgen (TB85.182, C-F-103466), 400 m, with *Salix herbacea* and *Harrimanella hypnoides* in snowbed. Nanortalik, 60.08°N, 45.14°W, 12 Aug 1991, T. Borgen (TB91.136, C-F-103465), 50 m, with *Salix* sp. Nanortalik municipality, Qinngua valley, 60.14°N, 45°W, 9 Aug 1991, T. Borgen (TB91.112, C-F-119782), 230 m, with *Salix glauca* in scrubland. Narsaq, 60.91°N, 46.05°W, 13 Aug 1993, E. Rald (ER 93.330, C-F-104550), 20 m, with *Salix glauca* and *Betula glandulosa* in heathland. Narsaq, 60.91°N, 46.05°W, 3 Aug 1993, E. Rald (ER 93.152, C-F-104318), 20 m. Narsarsuaq, Hospitalsdalen, 61.1715°N, 45.41°W, 10 Aug 1984, T. Læssøe (TL 84.608, C-F-119794), 60 m. Paamiut, 62.01°N, 49.4°W, 8 Aug 1981, T. Borgen (TB81.112, C-F-103552), 10 m, with *Salix glauca*. Paamiut, 62.01°N, 49.4°W, 27 Aug 1993, T. Borgen (TB93.159, C-F-103496), 20 m, with *Salix herbacea*. Paamiut, 62.01°N, 49.4°W, 31 Aug 1995, T. Borgen (TB95.114, C-F-103504), 30 m, with *Salix glauca*, *Betula glandulosa* in heathland. Paamiut, 62.01°N, 49.4°W, 6 Sep 1990, T. Borgen (TB90.087, C-F-103540), 10 m, with *Salix glauca*. Paamiut, 62.01°N, 49.4°W, 6 Sep 1990, T. Borgen (TB90.084a, C-F-103549), 10 m, with *Salix glauca*. Paamiut, 62.01°N, 49.4°W, 8 Sep 1990, T. Borgen (TB90.104a, C-F-103550), 10 m, with *Salix glauca* and *Salix herbacea*. Paamiut, 61.99°N, 49.66°W, 22 Aug 2008, T. Borgen (TB08.157, C-F-106752), 15 m, with *Salix glauca* and *Bistorta vivipara*. Paamiut, 62.01°N, 49.4°W, 31 Aug 1993, T. Borgen (TB93.183, C-F-103497), 50 m, with *Salix glauca* in heathland. Paamiut, 62.01°N, 49.4°W, 8 Sep 1993, T. Borgen (TB93.210, C-F-103499), 10 m. Paamiut, 62.01°N, 49.4°W, 1 Sep 1993, T. Borgen (TB93.187, C-F-103498), 10 m, with *Salix herbacea*. Paamiut, 62.01°N, 49.4°W, 28 Aug 1993, T. Borgen (TB93.155, C-F-103500), 10 m, with *Salix herbacea* in snowbed. Paamiut, Kangilineq /Kvaneøen, 61.95°N, 49.47°W, 25 Aug 1985, T. Borgen (TB85.250, C-F-119786), 20 m, with *Salix arctophila* in fenland. Paamiut, Navigation School area, 62.01°N, 49.4°W, 13 Aug 1990, T. Borgen (TB90.032, C-F-103543), 10 m, with *Salix herbacea* in snowbed. Paamiut, Navigation School area, 62.01°N, 49.4°W, 13 Aug 1990, T. Borgen (TB90.019, C-F-103544), 10 m, with *Salix herbacea* and *Bistorta vivipara*. Paamiut, Taartoq/Mørke Fiord, 61.99°N, 49.66°W, 29 Aug 1998, T. Borgen (TB98.201, C-F-104292), 25 m, with *Salix glauca*. Qaqortoq, 60.72°N, 46.04°W, 13 Aug 1993, E. Rald (ER 93.302, C-F-104551), 30 m, with *Salix glauca*. **W-Greenland**: Kangerlussuaq, 67.01°N, 50.72°W, 11 Aug 1986, T. Borgen (TB86.203, C-F-103568), 50 m. Kangerlussuaq, near a glacier, 67.03°N, 50.64°W, 12 Aug 2000, E. Ohenoja (EO12.8.00.1, OULU F050224), 229 m. Kangerlussuaq, c. 2 km W of the Airport, Mt. Hassel, 67.012°N, 50.856°W, 10 Aug 2000, A-M. Larsen, T. Borgen (TB00.069, C-F-103515), 50 m, with *Salix glauca* in copse. Kangerluarsunnguaq, Kobbefjord, end of fiord, 64.14°N, 51.35°W, 26 Aug 2018, S.A. Elborne (SAE-2018.357-GR, C-F-112904), 100 m, with *Salix glauca* in copse. NW below Nasaasaaq, E-valley, E of Sisimiut, 66.93°N, 53.61°W, 18 Aug 2000, E. Horak (ZT8901, ZT8901), 50 m, with *Salix glauca*. **N-Greenland**: Daneborg, 0.5 km E of Airstrip, 74.2°N, 20.1°W, 29 Jul 2006, T. Borgen (TB06.033, C-F-119770), 20 m, with *Dryas* in heathland. Daneborg, slope NW of The Weather Station, 74.2°N, 20.1°W, 31 Jul 2006, T. Borgen (TB06.061, C-F-119769), 20 m, with *Dryas* sp. in heathland. Zackenberg, 74.5°N, 21°W, 3 Aug 2006, T. Borgen (TB06.081, C-F-119777), 50 m, with *Dryas integrifolia* and *Dryas octopetala* in scrubland. Zackenberg, 74.5°N, 21°W, 7 Aug 2006, T. Borgen (TB06.120, C-F-119765), 50 m, with *Dryas* in heathland. Zackenberg, 74.5°N, 21°W, 2 Aug 2006, T. Borgen (TB06.067, C-F-119743), 50 m, with *Dryas* sp. and *Salix arctica* in scrubland. Zackenberg, Aucellabjerg, 74.5°N, 21°W, 27 Jul 1999, T. Borgen (TB99.118, C-F-119756), 150 m, with *Dryas* sp. and *Salix arctica* in scrubland. Zackenberg, Aucellabjerg, 74.5°N, 21°W, 20 Aug 2006, T. Borgen (TB06.250, C-F-119764), 400 m, with *Dryas* sp., *Bistorta vivipara* and grassland. Zackenberg, Aucellabjerg, 74.5°N, 21°W, 9 Aug 2006, T. Borgen (TB06.128, C-F-119767), 300 m, with *Dryas* sp. in heathland. Zackenberg, Aucellabjerg, 74.5°N, 21°W, 11 Aug 1999, T. Borgen (TB99.280, C-F-119802), 150 m, tundra. Zackenberg, between West River and Solkæret, 74.5°N, 21°W, 9 Aug 1999, T. Borgen (TB99.258, C-F-119745), 30 m, with *Dryas* sp. and *Salix arctica* in scrubland. Zackenberg, just W of Kærelv, 74.5°N, 21°W, 30 Jul 1999, T. Borgen (TB99.160, C-F-119809), 30 m, with *Salix arctica* and *Bistorta vivipara* in solifluction lobe in snowbed. Zackenberg, shortly E of Kærelv, 74.5°N, 21°W, 13 Aug 1999, T. Borgen (TB99.304, C-F-119803), 50 m, with *Dryas* sp. in heathland. Zackenberg, Ulvehøj, 74.5°N, 21°W, 29 Jul 1999, T. Borgen (TB99.146, C-F-104296), 40 m, with *Salix arctica* in scrubland. **E-Greenland**: Jameson Land, Nerlerit Inaat/Constable Pynt, delta of Gåseelv valley, 70.76°N, 22.65°W, 8 Aug 2017, T. Borgen (TB17C.078, C-F-106777), 40 m, with *Salix glauca* in copse. Jameson Land, Nerlerit Inaat/Constable Pynt, Primulaelv, 70.74°N, 22.67°W, 7 Aug 2017, T. Borgen (TB17C.072, C-F-106776), 180 m, with *Bistorta vivipara* in heathland.

#### Distribution.

One of the five most commonly recorded *Hebeloma* species in Greenland, with 11.6% of the records. Common and widespread in Europe; also recorded from other parts of North America (https://mycoportal.org/portal/collections/list.php, accessed 2 Dec 2020). To our knowledge not recorded from alpine sites in Europe (Beker et al. 2016).

#### Habitat and ecology.

Forty-four collections, mainly with *Salix glauca* (15), *Dryas* (10) and *S. herbacea* (6). Minor hosts are *S. arctophila* and *S. arctica*, and two collections were difficult to separate from the roots of *Bistorta vivipara*. Found in all types of habitats from scrubland, grassland and heathland to snowbeds. In the Rocky Mountains, the hosts are *S. arctica*, *S. glauca*, *S. planifolia*, *S. reticulata*, *Betula nana* and *Dryas* (Cripps et al. 2019). For lowland Europe, there is a long list of deciduous trees as probable hosts (Beker et al. 2016).

##### *Hebeloma* sect. *Velutipes Vesterh.*; Ann. Micol. A. G. M. T. 1: 60, 2004.

Stem velute, usually pruinose at least at apex, bulbous. Cheilocystidia gently clavate towards the apex, occasionally ventricose, spores rather strongly to very strongly dextrinoid.

### Hebeloma leucosarx

Taxon classificationFungiAgaricalesHymenogastraceae

P.D. Orton; Trans. Br. mycol. Soc. 43(2): 244, 1960.

512E1B68-4C86-5075-B31E-A43592DD1EA5

Fig. 32

#### Macroscopic description.

Cap 1.8–9.0 cm in diameter, convex, later umbonate, sometimes turned upwards with age, margin often involute when young, later smooth or eroded or wavy, tacky when moist, rarely spotted, sometimes hygrophanous, usually bicolored, often with thin margin but may be unicolored when young, at center dark pinkish buff to ochraceous or dark olive buff or yellowish brown to clay buff or cinnamon to umber or brick, at margin cream to honey or pinkish buff to ochraceous or dark olive buff or clay-pink, without any remains of veil. Lamellae light gray brown to vinaceous buff, adnate to emarginate, maximum depth 2.5–9 mm, number of lamellae {L} 50–70, droplets usually visible but sometimes absent, white fimbriate edge present. Stem (3.0–)3.7–11.0 × 0.3–1.4 {median} × 0.8–2.0 {base} cm, stem Q (6–)6.8–14(–17.5), whitish, often clavate to bulbous, sometimes cylindrical, pruinose to floccose, particularly at apex, sometimes more velutinate, sometimes with mycelial chords. Context firm, stem interior hollow, sometimes with superior wick, flesh discoloring from base. Smell raphanoid, sometimes with hint of cacao. Taste raphanoid, sometimes weakly bitter. Spore deposit brownish olive to umber.

**Figure 32.**
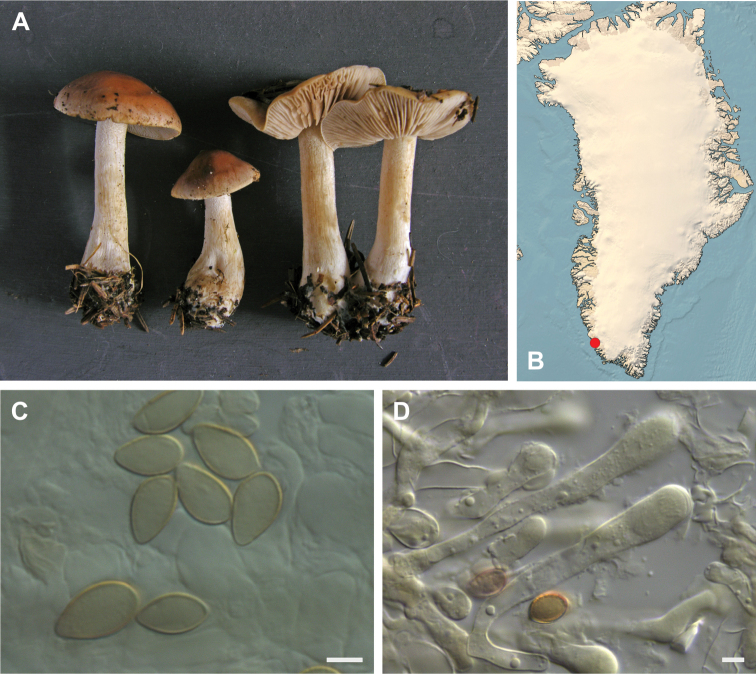
*Hebeloma leucosarx***A** JV06-757 (from Denmark), photo J. Vesterholt, reproduced by kind permission from Beker et al. (2016)**B** distribution of cited collections **C** spores ×1600 and **D** cheilocystidia ×1000 of TB81.211 in Melzer’s reagent. Scale bars: 5 µm; microphotographs H.K.J. Beker.

#### Microscopic description.

Spores amygdaloid, occasionally limoniform, not or weakly papillate, on ave. 9.5–12.0 × 5.5–7.0 µm, ave. Q = 1.6–2.0 yellow brown to brown, guttulate, almost smooth to weakly ornamented but occasionally distinctly ornamented (O1O2O3), perispore not or somewhat loosening (P0P1), weakly to strongly dextrinoid, reaction often very slow (D2D3 (D4)). Basidia 24–33(–35) × 7–8(–10) µm, ave. Q = 3–4.1, mostly four-spored. Cheilocystidia slenderly clavate, occasionally clavate-stipitate or ventricose, occasionally with characteristic apical wall thickening, occasionally bifurcate, geniculate or septate, on ave. 41–67 × 6.5–8.5 (apex) × 4–5.5 (middle) × 4.5–6.5 (base) µm, ratios A/M = 1.42–1.72, A/B = 1.15–1.68, B/M = 0 .94–1.33. Epicutis an ixocutis, 80–200 thick (measured from exsiccata), maximum hyphae width 5 µm, sometimes encrusted, trama elements beneath subcutis ellipsoid to sausage-shaped, occasionally polygonal up to 20 µm wide. Caulocystidia similar to cheilocystidia, up to 200 µm long, often septate and markedly lageniform.

#### Collections examined.

**S-Greenland**: Paamiut, head of Eqaluit, median part, 62.03°N, 49.25°W, 15 Aug 1998, T. Borgen (TB98.119, C-F-103513), 300 m, with *Betula glandulosa* and *Salix glauca* in heathland. Paamiut, Taartoq/Mørke Fiord, 62.01°N, 49.26°W, 29 Aug 1981, T. Borgen (TB81.211, C-F-103551), ca. 100 m, with *Betula glandulosa* in heathland.

#### Distribution.

Only two records, both from the same area. The general distribution of the species is temperate, to the middle boreal zone. The Greenland records are both from low arctic areas. *Hebeloma leucosarx* is missing from lowland regions of southern Europe (Beker et al. 2016; Grilli et al. 2020).

#### Habitat and ecology.

Among the 28 species of *Hebeloma* found in Greenland, *H. leucosarx* is the only species that may primarily be associating with *Betula* rather than *Salix*, based on the observations of Beker et al. (2016) from Europe. According to their monograph, in Europe, the main hosts are conifers and *Betula* (Beker et al. 2016); for the above records, the host is most likely *B. glandulosa*, although in one record *S. glauca* is mentioned as present.

### Hebeloma subconcolor

Taxon classificationFungiAgaricalesHymenogastraceae

Bruchet; Bull. mens. Soc. linn. Lyon 39 (6 (Suppl.)): 127, 1970.

CA7F0D3A-3A6D-5035-951E-FADB57B34F9C

Fig. 33

#### Macroscopic description.

Cap 0.8–2.1 cm, convex to umbonate, sometimes broadly, margin smooth, sometimes involute, tacky when moist, usually almost unicolored, at center clay buff to gray brown to dark olive buff to sepia, sometimes pruinose particularly when young, margin sometimes paler, even cream, veil absent. Lamellae when young whitish, later distinctly gray, adnate to emarginate, 3–4 mm broad, number of lamellae {L} 20–32, droplets usually visible with naked eye, but occasionally only with × 10 lens or absent, edge white fimbriate. Stem 1.5–4.5 × 0.3–0.6 cm, {median} × 0.35–0.75 {base} mm, velute, usually pruinose at apex, cylindrical, base clavate or sometimes bulbous. Context firm, in stem stuffed, later hollow, discoloring brownish from base. Smell raphanoid, sometimes strongly. Taste bitter, raphanoid. Spore deposit clay buff.

**Figure 33.**
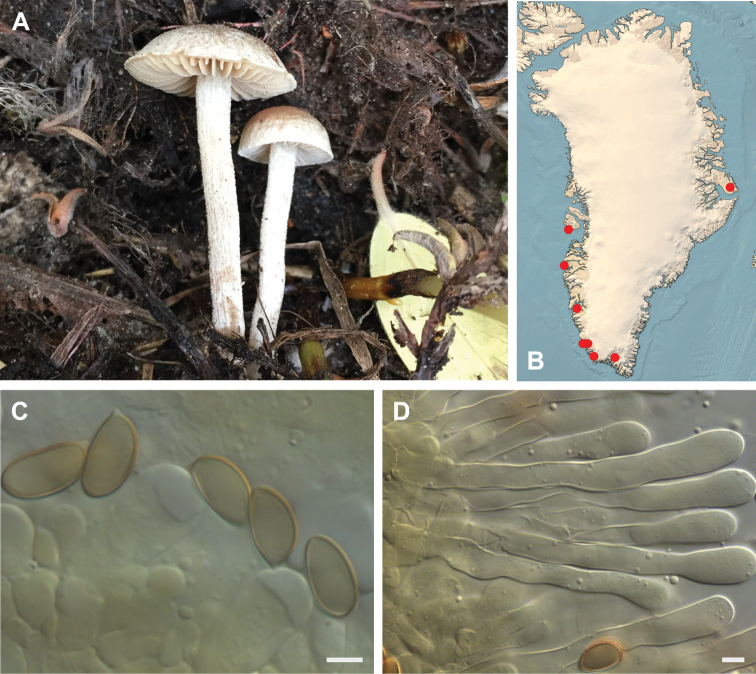
*Hebeloma subconcolor***A** SAE-2016.090, photograph S.A. Elborne **B** distribution of cited collections **C** spores ×1600 and **D** cheilocystidia ×1000 of SAE-2016.090 in Melzer’s reagent. Scale bars: 5 µm; microphotographs H.K.J. Beker.

#### Microscopic description.

Spores amygdaloid, limoniform, sometimes weakly papillate, on ave. 10.5–12.5 × 6.5–7.0 µm, ave. Q 1.6–1.85, usually guttulate, pale, yellow brown to brown, almost smooth to very weakly ornamented (O1O2), perispore not or somewhat loosening (P0P1), weakly to rather strongly dextrinoid (D2D3). Basidia 26–33(–36) × 8–9 µm, Q = 3.4–3.9, mostly four-spored. Cheilocystidia slenderly clavate, sometimes cylindrical, clavate-lageniform or ventricose, occasionally with a characteristic apical wall thickening, occasionally bifurcate, geniculate or septate, on ave. 47–69 × 6.5–9 (apex) × 5–6.5 (middle) × 5–7.5 (base) µm, ratios A/M = 1.36–1.71, A/B = 1.22–1.86, B/M = 0 .92–1.26. Epicutis an ixocutis, 60–75 µm (measured from dried specimens), maximum hyphae width 5.5–6 µm, sometimes encrusted, shape of trama elements beneath subcutis ellipsoid, isodiametric, sausage-shaped up to 20 µm wide. Caulocystidia similar to cheilocystidia, up to 120 µm long and 11 µm wide, multi-septate.

#### Collections examined.

**S-Greenland**: Kangilinnguit-Ivittuut, 61.21°N, 48.12°W, 18 Aug 2018, H. Knudsen (HK18.232, C-F-111111), 125 m, in tundra. Narsarsuaq, 61.17°N, 45.40°W, 17 Aug 2015, H. Knudsen (HK15.089, C-F-8242), 60 m, with *Salix glauca*. Nuuk, Qooqqut, 64.26°N, 50.92°W, 15 Aug 1987, T. Borgen (TB87.117, C-F-4002), ca. 30 m, with *Salix glauca* in ditch. Paamiut, 61.99°N, 49.66°W, 4 Aug 1993, E. Rald (ER 93.168, C-F-104313), 25 m. Paamiut, 62.01°N, 49.4°W, 14 Aug 1990, T. Borgen (TB90.033, C-F-104299), 25 m. Paamiut, N of the Navigation School area, 62.02°N, 49°W, 3 Aug 1990, T. Borgen (TB90.018, C-F-119761), ca. 40 m, with *Bistorta vivipara* and *Salix herbacea*. **W-Greenland**: Disko, Fortune Bay, 69.31°N, 53.88°W, 3 Aug 1986, T. Borgen (TB86.122, C-F-103587), 20 m. Sisimiut, south of town, 66.95°N, 53.66°W, 18 Aug 2016, S.A. Elborne (SAE-2016.090-GR, C-F-106739), 20 m, with *Salix glauca* in copse. **E-Greenland**: Jameson Land, Constable Pynt, Ugleelv, 70.88°N, 22.85°W, 24 Jul 1989, H. Knudsen (HK89.302, C-F-2195), 100 m.

#### Distribution.

*Hebeloma subconcolor* is a truly arctic-alpine species with nine records from low and high arctic areas in Greenland. It was recently reported from two collections from alpine North America (Colorado, Cripps et al. 2019), but the records here are the first from arctic North America. Described from the European Alps by Bruchet 50 years ago, it is still only known from few other locations and it must be considered a rather rare species.

#### Habitat and ecology.

Nine collections of *H. subconcolor* are verified, but only sparse info is given on hosts and ecology. *Salix glauca*, *S. herbacea* and *Bistorta vivipara* are mentioned as possible hosts. Most localities are on acid soil in agreement with the conclusion of Beker et al. (2016), who, in Europe, had *S. herbacea* listed as possible host for each cited collection of the species.

### Hebeloma velutipes

Taxon classificationFungiAgaricalesHymenogastraceae

Bruchet; Bull. Mens. Soc. Linn. Lyon 39(6(Suppl.)): 127, 1970.

B920593C-040A-51A2-8427-F03F1F834CAC

Fig. 34

#### Macroscopic description.

Cap 1.6–8.2 cm in diameter, convex to umbonate, margin often involute when young, sometimes crenulate, occasionally upturned and wavy with age, tacky when moist, occasionally spotted, not hygrophanous, unicolored or variably bicolored, at center whitish to cream or buff to ochraceous or more rarely dark olive buff or yellowish brown or brownish olive, at margin white to cream or buff, without remains of veil. Lamellae clay brown, adnate to emarginate, occasionally with decurrent tooth, maximum depth 2–9 mm, number of lamellae {L} 50–78, droplets visible, occasionally only visible with × 10 lens, rarely absent, white fimbriate edge present, sometimes very distinct. Stem 0.5–10.4 × 0.3–1.6 {median} × 0.4–2.7 {base} cm, stem Q (0.6–)2.5–12.1(–14.4), whitish, base usually clavate to bulbous, sometimes cylindrical, usually velutinate, often pruinose or floccose at least on the upper half. Context firm, stem interior stuffed, later hollow, often with superior wick, occasionally with basal wick, flesh generally not discoloring from base. Smell usually raphanoid, sometimes earthy. Taste usually bitter and raphanoid. Spore deposit brownish olive to umber.

**Figure 34.**
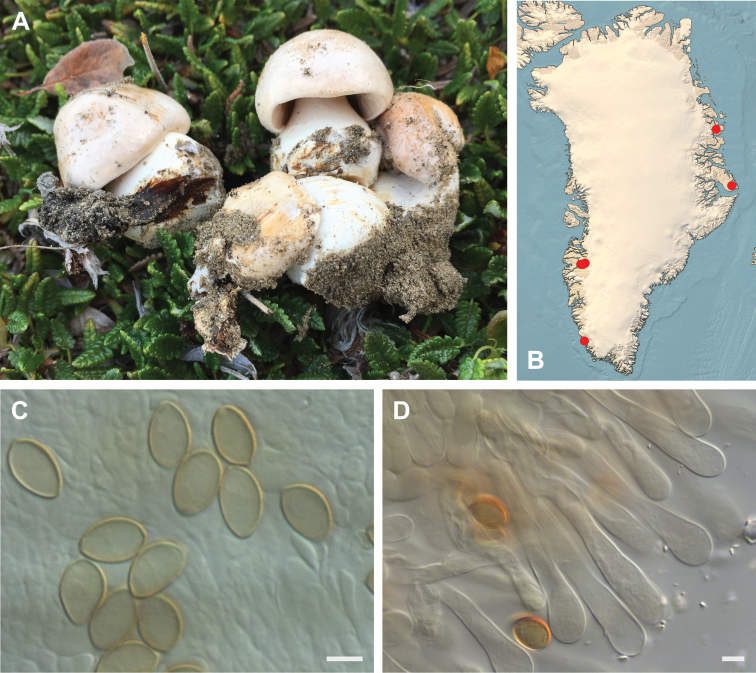
*Hebeloma velutipes***A** SAE-2017.110, photograph S.A. Elborne **B** distribution of cited collections **C** spores ×1600 and **D** cheilocystidia ×1000 of SAE-2017.110 in Melzer’s reagent. Scale bars: 5 µm; microphotographs H.K.J. Beker.

#### Microscopic description.

Spores amygdaloid, occasionally limoniform, variably papillate, but usually at most weakly, on ave. 9–13 × 5.5–7.5 µm, Q = 1.5–1.9, yellow through yellow brown to brown, usually guttulate, almost smooth to very weakly ornamented (O1O2 (O3)), perispore not or somewhat loosening (P0P1), rather strongly dextrinoid ((D2) D3 (D4)). Basidia 24–38(–43) × 6–10 µm, ave. Q = (3–)3.5–4.9, mostly four-spored. Cheilocystidia slenderly clavate, some clavate-lageniform, cylindrical or ventricose, more rarely clavate-stipitate, occasionally characteristically bifurcate, geniculate or septate (sometimes clamped), on ave. 43–73 × 6.5–9 (apex) × 4–6 (middle) × 4–7 (base) µm, ratios A/M = 1.31–1.73, A/B = 1.07–1.73, B/M = 0.86–1.34. Epicutis an ixocutis, 80–200 µm thick (measured from exsiccata), maximum hyphae width 4–8 µm, sometimes encrusted, trama elements beneath subcutis cylindrical, ellipsoid, isodiametric, sausage-shaped up to 12 µm wide. Caulocystidia similar to cheilocystidia, but more irregular, up to 200 µm long.

#### Collections examined.

**S-Greenland**: Paamiut, 62.01°N, 49.4°W, 19 Aug 1998, T. Borgen (TB98.158, C-F-103512), 75 m, with *Salix glauca* in tundra. **W-Greenland**: Kangerlussuaq near the Ice cap, 67.10°N, 50.23°W, 12 Aug 2000, A-M. Larsen, T. Borgen (TB00.073, C-F-103519), 220 m, with *Salix glauca* in copse. Kangerlussuaq, airport area, 67.04°N, 50.41°W, 10 Aug 1986, T. Borgen (TB86.179, C-F-103557), 30 m, with *Salix glauca* and *Betula nana*. Kangerlussuaq, Ringsødalen, Kellyville, 66.99°N, 50.95°W, 14 Aug 2000, S.A. Elborne (SAE-2000.041-GR, C-F-108492), 180 m, at lakeside. Kangerlussuaq, Sandflugtsdalen, c. 15 km E of of the airport, 67.07°N, 50.46°W, 8 Aug 2016, T. Borgen (TB16.087, C-F-103582), 200 m, with *Salix glauca* and *Sphagnum* in scrubland. Kangerlussuaq, Store Saltsø, 66.99°N, 50.59°W, 15 Aug 2000, S.A. Elborne (SAE-2000.051-GR, C-F-108502), 260 m, with *Betula nana* in heathland. **N-Greenland**: Zackenberg, Aucellabjerg, at Kærelv, 74.5°N, 21°W, 14 Aug 1999, T. Borgen (TB99.336, C-F-119749), 100 m, with *Dryas* sp. and *Salix arctica* in scrubland. Zackenberg, W of Kærelv, 74.5°N, 21°W, 13 Aug 1999, T. Borgen (TB99.309, C-F-119754), 40 m, with *Dryas* sp. in scrubland. **E-Greenland**: Jameson Land, Nerlerit Inaat/Constable Pynt, delta of Gåseelv valley, 70.76°N, 22.65°W, 9 Aug 2017, H. Knudsen (HK17.186, C-F-105090), 40 m. Jameson Land, Nerlerit Inaat/Constable Pynt, delta of Gåseelv valley, 70.76°N, 22.66°W, 6 Aug 2017, S.A. Elborne (SAE-2017.110-GR, C-F-106762), 65 m, with *Dryas* sp.

#### Distribution.

*Hebeloma velutipes* is one of the most common *Hebeloma* species in Europe and widely distributed all over Europe (Beker et al. 2016). In Greenland, it is also widespread, but relatively uncommon. From alpine Europe it is known from the Pyrenees, the Alps and Lower Tatra, and from arctic Europe from Svalbard and Iceland (Beker et al. 2016). Outside Europe and Greenland, it is known from alpine sites in the Rocky Mountains (Colorado, Montana, Cripps et al. 2019).

#### Habitat and ecology.

Ten collections, all but one (Paamiut) from calcareous localities. *Salix glauca* and *Dryas* are main hosts, one record is with *Betula nana*. In the Rocky Mountains, *H. velutipes* is also recorded with *Dryas octopetala*, *Salix glauca* and *S. reticulata* (Cripps et al. 2019). In Europe numerous hosts are recorded, see Beker et al. (2016).

##### Hebeloma sect. Naviculospora

### Hebeloma islandicum

Taxon classificationFungiAgaricalesHymenogastraceae

Beker & U. Eberh.; Beker, Eberhardt & Vesterholt, Fungi Europ. (Alassio) 14: 414, 2016.

793409B2-235C-5E1A-9E4F-1FC7FF32FB3E

Fig. 35

#### Macroscopic description.

Cap 2.0–4.0 cm in diameter, convex to umbonate, margin involute when young, smooth, tacky when moist, not hygrophanous, uniformly colored or bicolored, at center dark olive buff to yellowish brown, at margin cream, innately fibrillose, sometimes with remnants of universal veil. Lamellae initially pale clay, in age often brownish, emarginate, maximum depth 5 mm, number of lamellae {L} 40–50, droplets visible with naked eye, with white fimbriate edge. Stem 1.7–2.5 × 0.4–0.6 {median} × 0.5–0.7 {base} cm, stem Q 3.4–5.5, whitish pale, finely flocculose-tomentose in the entire length with clavate base. Context firm, stem interior stuffed, later hollow, flesh usually discoloring from base. Smell raphanoid. Taste mild or slightly bitter. Spore deposit not recorded.

**Figure 35.**
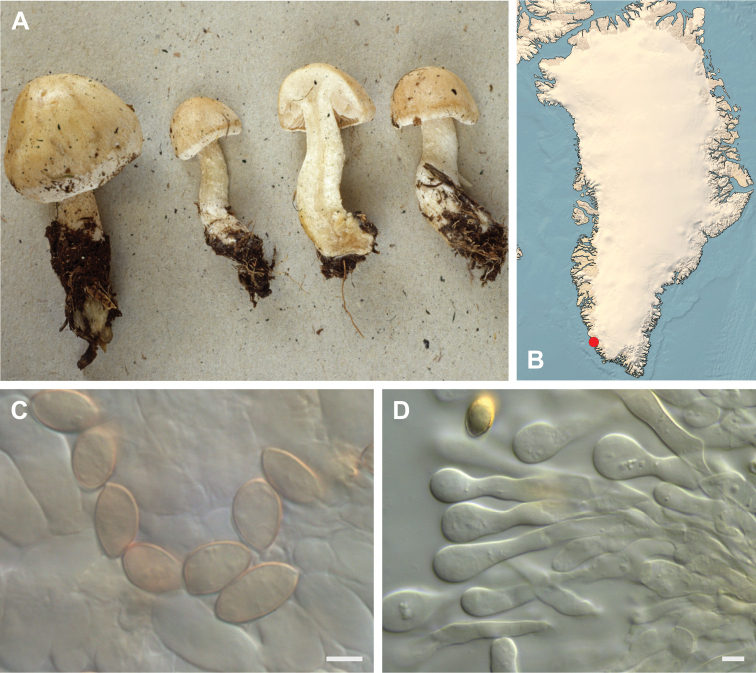
*Hebeloma islandicum***A** TB19.81, photograph T. Borgen **B** distribution of cited collections; **C** spores ×1600 and **D** cheilocystidia ×1000 of TB86.291 in Melzer’s reagent. Scale bars: 5 µm; microphotographs H.K.J. Beker.

#### Microscopic description.

Spores amygdaloid, often limoniform, papillate, on ave. 11.0–12.5 × 6.5–7.0 µm, ave. Q = 1.7–1.8, yellow to yellow brown, guttulate, at most weakly ornamented ((O1) O2), perispore not or somewhat loosening (P0P1), weakly to rather strongly dextrinoid (D2D3). Basidia 28–40 × 6–9 µm, ave. Q = 3.7–4.9, mostly four-spored. Cheilocystidia irregular, a mixture of clavate-stipitate, clavate-lageniform, ventricose and slenderly clavate, with occasional characters, geniculate, septate (sometimes clamped) or rostrate, on ave. 44–55 × 6.5–8.5 (apex) × 4–5 (middle) × 4–8 (base) µm, ratios A/M = 1.63–1.98, A/B = 1.04–1.85, B/M = 1.05–1.73. Epicutis an ixocutis, up to 120 µm thick (measured from exsiccata), maximum hyphae width 6 µm, some encrusted, trama elements beneath subcutis ellipsoid, cylindrical, thick sausage-shaped up to 20 µm wide. Caulocystidia irregular like cheilocystidia up to 140 µm long.

#### Collections examined.

**S-Greenland**: Paamiut, N of town, 62.01°N, 49.4°W, 5 Sep 1986, T. Borgen (TB86.291, C-F-103573), 10 m, with *Salix herbacea* in snowbed.

#### Distribution.

Only one record, from southern Greenland. Until recently only known from the type from the north-western, arctic part of Iceland. As discussed above, two more collections have been discovered from herbarium exsiccata at O; both, collected in Norway at above 60°N at altitudes of more than 1000 m (Eberhardt et al. in press). The above record from Greenland is from an arctic area and extends the distribution to North America. It is seemingly a very rare species. Although T.B. investigated the area around Paamiut regularly for 20 years, he only collected it once.

#### Habitat and ecology.

All four known collections appear to have been associated with *Salix herbacea* in a wet snowbed.

#### Notes.

*Hebeloma islandicum* was the only species recorded during the course of this study, which was not belonging to *H.* sects *Denudata*, *Hebeloma* or *Velutipes*. This species was described in Beker et al. (2016) on the basis of a single, but distinctive, collection from Iceland. The authors placed it within H. sect. Naviculospora. However, the authors acknowledged that it did not sit comfortably within any of their accepted sections. Within this section, this is the only species known to associate with *Salix*. Since its publication, two further collections have been discovered in Norway (Eberhardt et al. in press).

Molecularly, this species is unambiguous. The ITS of the collection presented here is almost identical with the ITS of the type (693/694 pos. identical, all positions are matching (one G/T in the type sequence matched by a clean G). Most similar, but always less than 98% similar, are *H. naviculosporum* and *H. nanum* (both H. sect. Naviculospora).

## Discussion

This is the first monograph of *Hebeloma* in Greenland. With 381 analyzed collections (378 with ITS sequences) and 28 species, this is also the study encompassing the largest number of collections of this genus in Greenland. The sample includes one species new to science, *H. arcticum*, and collections of two species, *H. islandicum* and *H. louiseae*, that have previously been reported only from Iceland and Norway or Svalbard, respectively. Because of the inconsistencies in the application of names and interpretation of species that prevailed for a long time, we here ignore earlier works.

Much of Greenland is not easily accessible and the attention that different collection sites received is directly linked to their geographic setting, the available infrastructure and, last but not least, the biography of the main collectors. Thus, even the main collecting sites are not directly comparable: Paamiut, where T.B. lived for 20 years and Narsarsuaq, the airport which was the main access to Greenland for all collectors, were more heavily and frequently forayed than Zackenberg where T.B. collected for two seasons, and Jameson Land that was only visited in 1989 and 2017. It appears likely that this is the reason why Paamiut has the highest number of species and may be why three species (*H. clavulipes*, *H. islandicum* and *H. leucosarx*) were collected only there. While it cannot be claimed that this sample of collections is a truly random set, it is the case that the collectors did try to collect samples of every *Hebeloma* they noticed, and they did visit a number of different sites across Greenland, over some 40 years. So, it is likely to be a fairly representative sample of the sites visited at the time they were visited.

This paper follows earlier publications (Eberhardt et al. 2015a, b, 2016; Beker et al. 2016; Grilli et al. 2016; Cripps et al. 2019) in the delimitation of species. Thus, morphology (sometimes in combination with ecology) takes the lead in the delimitation of species in the absence of a clear molecular signal. This is done under the assumption that, if the species are truly distinct, then more in-depth molecular studies will reveal differences in future. If this were not the outcome, it would be much easier lumping species later than revisiting material later in order to separate taxa.

Greenland is rich in species that are difficult to delimit from one another and particularly rich with regard to members of H. sect. Hebeloma: we have not yet found a locus (or set of loci) that unambiguously separates between all species. Here, as in the species complexes around *H. alpinum* and *H. velutipes*, we do not expect the evolution to be treelike, thus network analyses are a viable option. The sequence data we have is from dikarya (earlier attempts to ‘phase’ dikarya failed in many cases), and intragenomic variation occurs regularly in many species. Intragenomic variation is difficult to process in networks and sequence “variants” represented by a single circle may in fact differ by ambiguous positions or through the presences of indels. The numbers of collections roughly doubled from the datasets used by Cripps et al. (2019) that were used as starting point for these analyses. Largely, the results obtained here support the results of Cripps and co-workers and, in most cases, the intraspecific variation or the lack of interspecific variation within Greenland matches that of the sequences from other parts of the northern hemisphere.

Notable exceptions are *H. alpinicola*, which, with Greenland samples included, and the number of collections increased, appears less clearly distinct from *H. dunense* than it did in the earlier study. *Hebeloma helodes* and *H. aurantioumbrinum* are also less distinct in their ITS in Greenland than they appeared in Cripps et al. (2019).

New additions such as *H. arcticum*, *H. ingratum* and *H. louiseae* are all clearly distinct from other species. For *H. arcticum* and *H. louiseae* this is also confirmed in the tree analysis. *Hebeloma ingratum* has close relatives (*H. fragilipes* and *H. pseudofragilipes*) that are not considered here. Apparent geographical structure in some of the networks, i.e. *H. marginatulum*, *H. hiemale* or *H. velutipes*, may include information with regard to the recolonization of Greenland after the last glaciation, but numbers are too low to draw any conclusions.

Beker et al. (2016) reported 25 species of *Hebeloma* that occur in arctic-alpine habitats of Europe, including Svalbard, out of 84 species for the whole of Europe. Five of the 25 species (*H. aanenii*, *H. laterinum*, *H. pallidolabiatum*, *H. perexiguum* and *H. salicicola*) have not yet been recorded in Greenland. With regard to *H. pallidolabiatum* and *H. perexiguum*, these species are, to date, only known from Svalbard and appear to be very rare. They may be endemic to Svalbard or simply not yet discovered in other areas of the Polar Regions. Three species, *H. arcticum*, *H. colvinii*, *H. excedens*, are currently only recorded from North America, including Greenland. *Hebeloma alpinicola* is described from North America, but was recently also found in Europe (Grilli et al. 2020). Also, a further four species, included here within the arctic funga of Greenland, are known from Europe but have never yet been recorded in arctic or alpine habitats. These are: *H. clavulipes*, *H. helodes*, *H. hygrophilum* and *H. leucosarx*. This means, that we now have 33 species of *Hebeloma* that have been recorded in arctic or alpine habitats.

Species of *Hebeloma* present in arctic and alpine habitats were classified by Beker et al. (2016) as “specialists” and “opportunists”. Specialists were defined as species that are widespread in such habitats, whereas opportunists are species that are more common in boreal habitats but are occasionally found in arctic and alpine habitats. Table 3 shows the classification into these two categories for the species confirmed for Greenland.

It is often impossible to remove ambiguous host information from collection metadata. Only in a few cases is a single host recorded as present. Further, if the collector is not aware of a potential host association, the person is likely not to record the presence of such hosts. A number of potential host genera and species have been named in different parts of this paper. While *Salix* appears to be the most common ectomycorrhizal host, there are cases, as noted above, when *Dryas*, rather than *Salix*, may be the symbiont; this appears to be the case, particularly in dry and calcareous localities and appears especially true for *H. alpinum* and *H. hiemale*, for which we have found *Dryas* to be the only or the closest possible symbiont on several occasions. Less frequently, we found *Dryas* as the likely symbiont for *H. dunense*, *H. marginatulum*, *H. mesophaeum*, *H. pubescens*, *H. vaccinum*, and *H. velutipes*. A few collections were made under Alnus alnobetulae ssp. crispa, but, in every case, other possible associates were almost certainly present. Also, a number of collections were made where *Bistorta* was the most likely symbiont.

We have no reason to assume that host associations of *Hebeloma* species are fundamentally different in Greenland from other arctic or alpine areas of the world. Sequences from root samples of *B. vivipara* from Svalbard suggested an association with members of *H.* sects *Denudata* and *Hebeloma* (Brevik et al. 2010, Beker et al. 2018). While all 25 arctic or alpine species discussed in Beker et al. (2016) appeared able to associate with *Salix*, that is not clearly true for the additional species recorded from Greenland. In particular, according to Beker et al., *H. leucosarx* appears to associate most commonly with *Betula* or conifers. However, this may be an exception, and, for the most part, sections of *Hebeloma* that do not include species associating with *Salix*, are lacking from Greenland. Thus, compared to Europe, the Greenland funga does not include any species of H. sects *Duracinus*, *Myxocybe*, *Naviculospora* (other than *H. islandicum*, which does not sit comfortably within that section), *Porphyrospora*, *Pseudoamarescens*, *Scabripora*, *Sinapizantia*, *Syrjense* or *Theobromina*. The only section that includes species associating with *Salix* (Beker et al. 2016) that has not been collected in Greenland, or other arctic areas, as far as we are aware, is H. sect. Sacchariolentia.

*Hebeloma* species associate with all important host groups in Greenland (*Bistorta*, *Dryas* and *Salix*) and MOTUs (molecular operational taxonomic units) assigned to the genus have been retrieved consistently in metagenomics studies of arctic or alpine habitats including these hosts, often among the more often retrieved ectomycorrhizal fungi (e.g. Bjorbækmo et al. 2010; Timling et al. 2012; Mundra et al. 2015; Morgado et al. 2016 and many others). It is often not possible to assign such MOTUs to species with any degree of certainty (Eberhardt et al. 2018), but it does appear that *Hebeloma* is one of the important players in the ectomycorrhizal communities of arctic habitats.

## Supplementary Material

XML Treatment for Hebeloma

XML Treatment for Hebeloma alpinicola

XML Treatment for Hebeloma clavulipes

XML Treatment for Hebeloma colvinii

XML Treatment for Hebeloma dunense

XML Treatment for Hebeloma excedens

XML Treatment for Hebeloma fuscatum

XML Treatment for Hebeloma grandisporum

XML Treatment for Hebeloma hygrophilum

XML Treatment for Hebeloma marginatulum

XML Treatment for Hebeloma mesophaeum

XML Treatment for Hebeloma nigellum

XML Treatment for Hebeloma oreophilum

XML Treatment for Hebeloma pubescens

XML Treatment for Hebeloma spetsbergense

XML Treatment for Hebeloma alpinum

XML Treatment for Hebeloma arcticum

XML Treatment for Hebeloma aurantioumbrinum

XML Treatment for Hebeloma geminatum

XML Treatment for Hebeloma helodes

XML Treatment for Hebeloma louiseae

XML Treatment for Hebeloma minus

XML Treatment for Hebeloma ingratum

XML Treatment for Hebeloma vaccinum

XML Treatment for Hebeloma hiemale

XML Treatment for Hebeloma leucosarx

XML Treatment for Hebeloma subconcolor

XML Treatment for Hebeloma velutipes

XML Treatment for Hebeloma islandicum
